# Development and validation of a decision support tool for the diagnosis of acute heart failure: systematic review, meta-analysis, and modelling study

**DOI:** 10.1136/bmj-2021-068424

**Published:** 2022-06-13

**Authors:** Kuan Ken Lee, Dimitrios Doudesis, Mohamed Anwar, Federica Astengo, Camille Chenevier-Gobeaux, Yann-Erick Claessens, Desiree Wussler, Nikola Kozhuharov, Ivo Strebel, Zaid Sabti, Christopher deFilippi, Stephen Seliger, Gordon Moe, Carlos Fernando, Antoni Bayes-Genis, Roland R J van Kimmenade, Yigal Pinto, Hanna K Gaggin, Jan C Wiemer, Martin Möckel, Joost H W Rutten, Anton H van den Meiracker, Luna Gargani, Nicola R Pugliese, Christopher Pemberton, Irwani Ibrahim, Alfons Gegenhuber, Thomas Mueller, Michael Neumaier, Michael Behnes, Ibrahim Akin, Michele Bombelli, Guido Grassi, Peiman Nazerian, Giovanni Albano, Philipp Bahrmann, David E Newby, Alan G Japp, Athanasios Tsanas, Anoop S V Shah, A Mark Richards, John J V McMurray, Christian Mueller, James L Januzzi, Nicholas L Mills, CoDE-HF investigators, Adam Singer, Judd Hollander, Humberto Villacorta, Evandro Tinoco Mesquita, Joel Coste, Patrick Jourdain, Kimiaki Komukai, Michihiro Yoshimura, Olivier Hanon, Jean-Sébastien Vidal, Peter Cameron, Louisa Lam, Ben Freedman, Tommy Chung, Sean P Collins, Christopher John Lindsell

**Affiliations:** 1 British Heart Foundation Centre for Cardiovascular Science, University of Edinburgh, Edinburgh, UK; 2 Usher Institute, University of Edinburgh, Edinburgh, UK; 3 Department of Biochemistry, Cochin Hospital, Assistance Publique-Hopitaux de Paris, Paris, France; 4 Department of Emergency Medicine, Princess Grace Hospital Center, Monaco, Principality of Monaco; 5 Cardiovascular Research Institute of Basel, Department of Cardiology, University Hospital Basel, Basel, Switzerland; 6 Department of Internal Medicine, University Hospital Basel, University of Basel, Switzerland; 7 Liverpool Heart and Chest Hospital, Liverpool, UK; 8 Inova Heart and Vascular Institute, Falls Church, VA, USA; 9 Division of Nephrology, University of Maryland School of Medicine, Baltimore, MD, USA; 10 University of Toronto, St Michael’s Hospital, Toronto, ON, Canada; 11 Heart Institute, Hospital Universitari Germans Trias i Pujol, Badalona, CIBERCV, Spain; 12 Department of Cardiology, Radboud University Medical Center, Nijmegen, Netherlands; 13 University of Amsterdam, Amsterdam, Netherlands; 14 Harvard Medical School, Boston, MA, USA; 15 Division of Cardiology, Massachusetts General Hospital, Boston, MA, USA; 16 BRAHMS, Thermo Fisher Scientific, Hennigsdorf, Germany; 17 Department of Emergency and Acute Medicine with Chest Pain Units, Charité – Universitätsmedizin Berlin, Campus Mitte and Virchow, Berlin, Germany; 18 Department of Internal Medicine, Radboud University Medical Center, Nijmegen, Netherlands; 19 Department of Internal Medicine, Division of Pharmacology and Vascular Medicine, Erasmus Medical Center, Rotterdam, Netherlands; 20 Department of Surgical, Medical and Molecular Pathology and Critical Care Medicine, University of Pisa, Pisa, Italy; 21 Department of Clinical and Experimental Medicine, University of Pisa, Pisa, Italy; 22 Christchurch Heart Institute, University of Otago, Christchurch, New Zealand; 23 Emergency Medicine Department, National University Hospital, Singapore; 24 Department of Internal Medicine, Krankenhaus Bad Ischl, Bad Ischl, Austria; 25 Department of Laboratory Medicine, Hospital Voecklabruck, Voecklabruck, Austria; 26 Institute for Clinical Chemistry, University Medical Centre Mannheim, Faculty of Medicine Mannheim, University of Heidelberg, Mannheim, Germany; 27 First Department of Medicine, University Medical Centre Mannheim, Faculty of Medicine Mannheim, University of Heidelberg, Mannheim, Germany; 28 University of Milan Bicocca, ASST-Brianza, Pio XI Hospital of Desio, Internal Medicine, Desio, Italy; 29 Clinica Medica, University Milan Bicocca, Milan, Italy; 30 Department of Emergency Medicine, Azienda Ospedaliero-Universitaria Careggi, Florence, Italy; 31 Department of Internal Medicine III, Division of Cardiology, University Hospital of Heidelberg, Ruprecht‐Karls University Heidelberg, Heidelberg, Germany; 32 London School of Hygiene and Tropical Medicine, London, UK; 33 Cardiovascular Research Institute, National University Heart Centre Singapore, Singapore; 34 British Heart Foundation Cardiovascular Research Centre, University of Glasgow, Glasgow, UK; * Contributed equally

## Abstract

**Objectives:**

To evaluate the diagnostic performance of N-terminal pro-B-type natriuretic peptide (NT-proBNP) thresholds for acute heart failure and to develop and validate a decision support tool that combines NT-proBNP concentrations with clinical characteristics.

**Design:**

Individual patient level data meta-analysis and modelling study.

**Setting:**

Fourteen studies from 13 countries, including randomised controlled trials and prospective observational studies.

**Participants:**

Individual patient level data for 10 369 patients with suspected acute heart failure were pooled for the meta-analysis to evaluate NT-proBNP thresholds. A decision support tool (Collaboration for the Diagnosis and Evaluation of Heart Failure (CoDE-HF)) that combines NT-proBNP with clinical variables to report the probability of acute heart failure for an individual patient was developed and validated.

**Main outcome measure:**

Adjudicated diagnosis of acute heart failure.

**Results:**

Overall, 43.9% (4549/10 369) of patients had an adjudicated diagnosis of acute heart failure (73.3% (2286/3119) and 29.0% (1802/6208) in those with and without previous heart failure, respectively). The negative predictive value of the guideline recommended rule-out threshold of 300 pg/mL was 94.6% (95% confidence interval 91.9% to 96.4%); despite use of age specific rule-in thresholds, the positive predictive value varied at 61.0% (55.3% to 66.4%), 73.5% (62.3% to 82.3%), and 80.2% (70.9% to 87.1%), in patients aged <50 years, 50-75 years, and >75 years, respectively. Performance varied in most subgroups, particularly patients with obesity, renal impairment, or previous heart failure. CoDE-HF was well calibrated, with excellent discrimination in patients with and without previous heart failure (area under the receiver operator curve 0.846 (0.830 to 0.862) and 0.925 (0.919 to 0.932) and Brier scores of 0.130 and 0.099, respectively). In patients without previous heart failure, the diagnostic performance was consistent across all subgroups, with 40.3% (2502/6208) identified at low probability (negative predictive value of 98.6%, 97.8% to 99.1%) and 28.0% (1737/6208) at high probability (positive predictive value of 75.0%, 65.7% to 82.5%) of having acute heart failure.

**Conclusions:**

In an international, collaborative evaluation of the diagnostic performance of NT-proBNP, guideline recommended thresholds to diagnose acute heart failure varied substantially in important patient subgroups. The CoDE-HF decision support tool incorporating NT-proBNP as a continuous measure and other clinical variables provides a more consistent, accurate, and individualised approach.

**Study registration:**

PROSPERO CRD42019159407.

**Figure:**
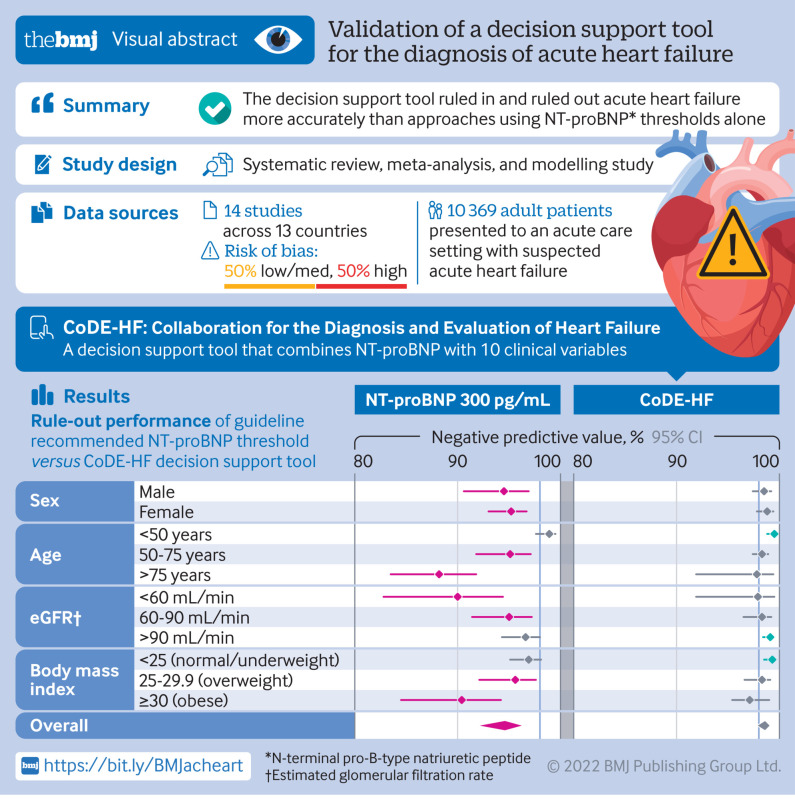


## Introduction

Nearly 1 million people are living with heart failure in the UK, and the prevalence is projected to rise by approximately 50% over the next 25 years owing to the ageing population.^1^ Decompensated acute heart failure accounts for 5% of all unplanned hospital admissions.^2^ The accurate and timely diagnosis of acute heart failure can be challenging, and both national and international guidelines recommend natriuretic peptide testing to aid in the diagnosis.^3^ ^4^ ^5^ ^6^ ^7^ ^8^ Despite these recommendations, N-terminal pro-B-type natriuretic peptide (NT-proBNP) testing has not been universally implemented, in part owing to concerns about its clinical utility in a real world setting. Studies investigating the diagnostic performance of NT-proBNP have mainly been conducted in relatively small, selected patient cohorts, limiting the generalisability of study findings across clinically important subgroups, such as older patients and those with renal disease or obesity, characteristics that are becoming increasingly prevalent in patients with heart failure.^9^ ^10^ ^11^ Statistical modelling approaches that incorporate patients’ characteristics to provide a more individualised assessment may have more consistent diagnostic performance across patient subgroups.^12^

Although many models have been developed to predict prognosis in patients with heart failure, very few have been developed to aid in the diagnosis of acute heart failure.^13^ ^14^ ^15^ ^16^ ^17^ ^18^ ^19^ Previous attempts have many strengths but have incorporated subjective variables, such as the clinician’s estimation of pre-test probability or the patient’s description of symptoms. Furthermore, they have incorporated NT-proBNP as a binary variable, which does not take into account the dynamic and non-linear interaction between NT-proBNP and other clinical variables. Previous attempts at developing and validating diagnostic scores have also included a limited number of patients from a single healthcare setting, which has precluded the assessment of performance within subgroups and limited external generalisability.

In this collaborative international analysis, we evaluated the diagnostic performance of guideline recommended NT-proBNP thresholds for acute heart failure across patient subgroups. We subsequently developed and validated a decision support tool for patients with suspected acute heart failure that uses statistical modelling to combine NT-proBNP concentrations with clinical characteristics.

## Methods

### Study population

We did a systematic review to identify studies that evaluated the diagnostic performance of NT-proBNP in patients with suspected acute heart failure. We updated a previous review by Roberts et al^1^ with studies published up to 18 August 2021 by searching titles and abstracts on Embase, Medline, and the Cochrane Central Register of Controlled Trials with the key words “heart failure” and “natriuretic peptide” (supplementary text 1). Studies were eligible if they met the following pre-specified inclusion criteria: enrolled patients aged ≥18 years with suspected acute heart failure in an acute care setting, measured NT-proBNP in blood samples obtained during the patients’ initial assessment on the day of the hospital attendance, and adjudicated diagnosis of acute heart failure by using an acceptable reference standard. Two investigators (KKL and MA) independently screened all studies identified in the systematic literature search, and a third (NLM) adjudicated conflicts by using a pre-specified protocol (PROSPERO register: CRD42019159407).

We contacted the corresponding authors of all eligible cohorts to request anonymised individual patient level data on NT-proBNP concentrations, adjudicated diagnosis of acute heart failure, demographics (age, sex, ethnicity), past medical history (heart failure, ischaemic heart disease, diabetes, hypertension, hyperlipidaemia, smoking, asthma, chronic obstructive pulmonary disease, chronic kidney disease), physiological variables during the initial assessment (heart rate and blood pressure), and clinical haematology and biochemistry profiles. We checked accuracy, definitions of variables, and completeness with all corresponding authors before harmonisation. All studies were conducted in accordance with the Declaration of Helsinki and with ethical approval to permit sharing of individual patient level data to conduct this meta-analysis. Two investigators (KKL and MA) independently assessed risk of bias for each study by using the Quality Assessment of Diagnostic Accuracy Studies version 2 (QUADAS-2) tool,^20^ with conflicts resolved by a third (NLM).

### NT-proBNP threshold analysis

We derived meta-estimates with 95% confidence intervals of the sensitivity, specificity, negative predictive value, and positive predictive value of the guideline recommended NT-proBNP rule-out threshold (300 pg/mL)^5^ ^8^ and age specific rule-in thresholds (450, 900, and 1800 pg/mL for patients aged <50, 50-75, and >75 years, respectively)^7^ for acute heart failure by using a two stage approach, with estimates calculated separately within each study and then pooled across studies in a binomial-normal random effects model using the DerSimonian and Laird method.^21^ We further evaluated the performance of these thresholds in pre-specified subgroups stratified by age, sex, ethnicity, body mass index, renal function, anaemia, and the presence of comorbidities (previous heart failure, hypertension, hyperlipidaemia, diabetes mellitus, atrial fibrillation, chronic obstructive pulmonary disease). Using the same approach, we subsequently evaluated the diagnostic performance of NT-proBNP concentrations across a range of concentrations to determine a rule-out threshold that would identify the highest proportion of patients as having a low probability of a negative predictive value ≥98% and a rule-in threshold that would identify the highest proportion of patients as having a high probability of a positive predictive value ≥75%.

### Model development and validation

We developed and validated a decision support tool (Collaboration for the Diagnosis and Evaluation of Heart Failure (CoDE-HF); https://decision-support.shinyapps.io/code-hf/) by using statistical modelling to compute a value (0-100) that corresponds to an individual patient’s probability of acute heart failure. Owing to significant differences in comorbidities and the prevalence of acute heart failure, we developed and validated models for patients with and without previous heart failure separately. We used NT-proBNP concentrations as a continuous measure and selected simple objective clinical variables that are known to be associated with acute heart failure, which were found to have the highest relative importance in our model training phase (age, estimated glomerular filtration rate, haemoglobin, body mass index, heart rate, blood pressure, peripheral oedema, chronic obstructive pulmonary disease, and ischaemic heart disease) (supplementary text 2).

We evaluated four different statistical models in the development of CoDE-HF: generalised linear mixed model, naïve Bayes, random forest, and extreme gradient boosting (XGBoost) (supplementary text 2).^22^ ^23^ ^24^ To account for missing data across studies (supplementary figure A), we multiply imputed 10 datasets by using joint modelling multiple imputation with random study specific covariance matrices fitted with a Markov chain Monte Carlo algorithm.^25^ We did multiple imputation for all variables included in the model except NT-proBNP. We ran 10 iterations of 10-fold cross validation for each model and used the median score across the iterations and imputed datasets as the CoDE-HF score for each patient. We subsequently identified the score that would classify the highest proportion of patients as having a high or low probability of acute heart failure with optimal performance to rule in (75% positive predictive value and 90% specificity) and rule out (98% negative predictive value and 90% sensitivity) acute heart failure.

We assessed the performance of each model across a range of diagnostic metrics (area under the receiver operator curve, Brier score, proportion of patients achieving the optimal high and low probability criteria, and positive and negative predictive value across patient subgroups). The Brier score is a measure of both discrimination and calibration and is calculated by taking the mean squared difference between predicted probabilities and the observed outcome.^26^ We selected the best performing model for the CoDE-HF decision support tool. We used a decision curve analysis and internal-external cross validation to evaluate the performance of CoDE-HF. In brief, this approach iteratively leaves out one study at a time for external validation and uses the remaining studies for model development.^27^ We did not do imputation in the external validation dataset, so external validation was not done for studies in which most of the variables were completely missing (supplementary figure A). We used R version 4.1.2 for all analyses.

### Patient and public involvement

Members of a patient and public panel were involved in the interpretation of results. There are plans to disseminate the results of the research to relevant patient communities.

## Results

### Study population

We contacted investigators from 30 eligible studies, of which 19 responded. Fourteen studies (12 prospective cohort studies and two randomised controlled trials) provided individual patient level data on 10 369 patients with suspected acute heart failure (mean age 69.3 years; 53.3% male) from 13 countries (table 1; supplementary figure B; supplementary tables A and B).^15^ ^28^ ^29^ ^30^ ^31^ ^32^ ^33^ ^34^ ^35^ ^36^ ^37^ ^38^ ^39^ ^40^ All studies were conducted in the emergency department except one that included patients admitted to the cardiology and pulmonology department (median number of patients in each study was 488 (interquartile range 322-1053)). Overall, 43.9% (4549/10 369) of patients had an adjudicated diagnosis of acute heart failure (median prevalence across studies 46% (31-54%)). Patients with a previous history of heart failure had a higher prevalence of acute heart failure than those without (73.3% (2286/3119) versus 29.0% (1802/6208)) (supplementary table C).

**Table 1:** Baseline characteristics of patients stratified by diagnosis of acute heart failure. Values are numbers (percentages) unless stated otherwise

Characteristics	Overall (n=10 369)	Patients with acute heart failure (n=4549)	Patients without acute heart failure (n=5820)
Male sex	5531 (53.3)	2568 (56.5)	2963 (50.9)
Mean (SD) age, years	69.3 (16.3)	75.0 (12.6)	64.9 (17.5)
Age group, years:			
<50	1377 (13.3)	222 (4.9)	1155 (19.8)
50-75	4370 (42.1)	1674 (36.8)	2696 (46.3)
>75	4622 (44.6)	2653 (58.3)	1969 (33.8)
Ethnicity:	(n=5700)	(n=2532)	(n=3168)
Black	845 (14.8)	316 (12.5)	529 (16.7)
White	4112 (72.1)	2028 (80.1)	2084 (65.8)
Other	743 (13.0)	188 (7.4)	555 (17.5)
Medical history:			
Previous heart failure (n=9327)	3119 (33.4)	2286 (55.9)	833 (15.9)
Ischaemic heart disease (n=9136)	2953 (32.3)	1871 (46.8)	1082 (21.0)
Diabetes mellitus (n=8967)	2398 (26.7)	1382 (34.8)	1016 (20.3)
Hypertension (n=8548)	5071 (59.3)	2603 (71.0)	2468 (50.5)
Hyperlipidaemia (n=5501)	2269 (41.2)	1160 (50.8)	1109 (34.5)
Current smoker or ex-smoker (n=5946)	2458 (41.3)	918 (37.8)	1540 (43.8)
Asthma (n=4153)	770 (18.5)	98 (6.9)	672 (24.6)
Chronic obstructive pulmonary disease (n=7249)	2117 (29.2)	670 (22.9)	1447 (33.5)
Atrial fibrillation (n=3588)	1701 (20.9)	1243 (33.3)	458 (10.4)
Chronic kidney disease (n=6441)	1215 (18.9)	877 (33.6)	338 (8.8)
Mean (SD) body mass index	27.7 (7.2)	27.7 (6.8)	27.7 (7.6)
Body mass index category:	(n=7852)	(n=3528)	(n=4324)
<25	3062 (39.0)	1349 (38.2)	1713 (39.6)
25-29	2473 (31.5)	1172 (33.2)	1301 (30.1)
≥30	2317 (29.5)	1007 (28.5)	1310 (30.3)
Physiological parameters:			
Mean (SD) heart rate, bpm	91.7 (23.7)	91.5 (25.9)	91.9 (21.9)
Mean (SD) systolic blood pressure, mm Hg	140.0 (27.9)	140.2 (30.0)	139.9 (26.4)
Mean (SD) diastolic blood pressure, mm Hg	79.7 (17.0)	80.3 (18.3)	79.3 (15.9)
Clinical haematology and biochemistry:			
Mean (SD) haemoglobin, g/dL	13.1 (2.1)	12.7 (2.1)	13.4 (2.0)
Mean (SD) eGFR, mL/min/1.73 m^2^	68.2 (31.3)	56.8 (27.1)	77.2 (31.6)
Median (IQR) NT-proBNP, pg/mL	1182 (191-4737)	4362 (1883-9883)	279 (70-1054)

eGFR=estimated glomerular filtration rate; IQR=interquartile range; NT-proBNP=N-terminal pro-B-type natriuretic peptide; SD=standard deviation.

### Guideline recommended and age specific NT-proBNP thresholds

**Fig 1:**
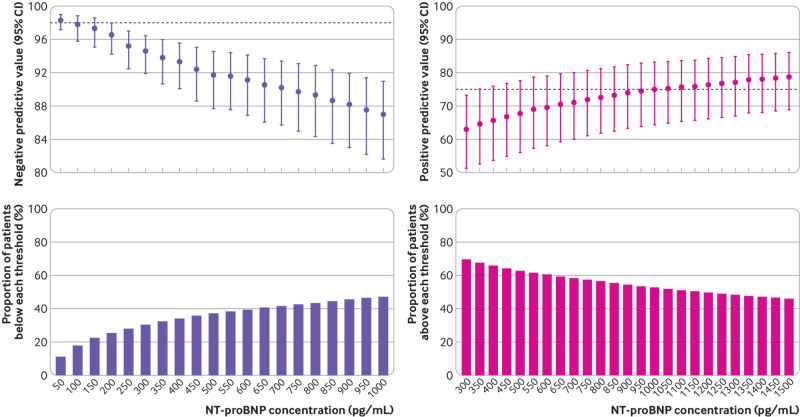
N-terminal pro-B-type natriuretic peptide (NT-proBNP) thresholds for acute heart failure. Top left: negative predictive values of NT-proBNP concentrations to rule out diagnosis of acute heart failure. Bottom left: cumulative proportion of patients presenting with suspected acute heart failure with NT-proBNP concentrations below each threshold. Top right: positive predictive values of NT-proBNP concentrations to rule in diagnosis of acute heart failure. Bottom right: cumulative proportion of patients presenting with suspected acute heart failure with NT-proBNP concentrations above each threshold

**Fig 2:**
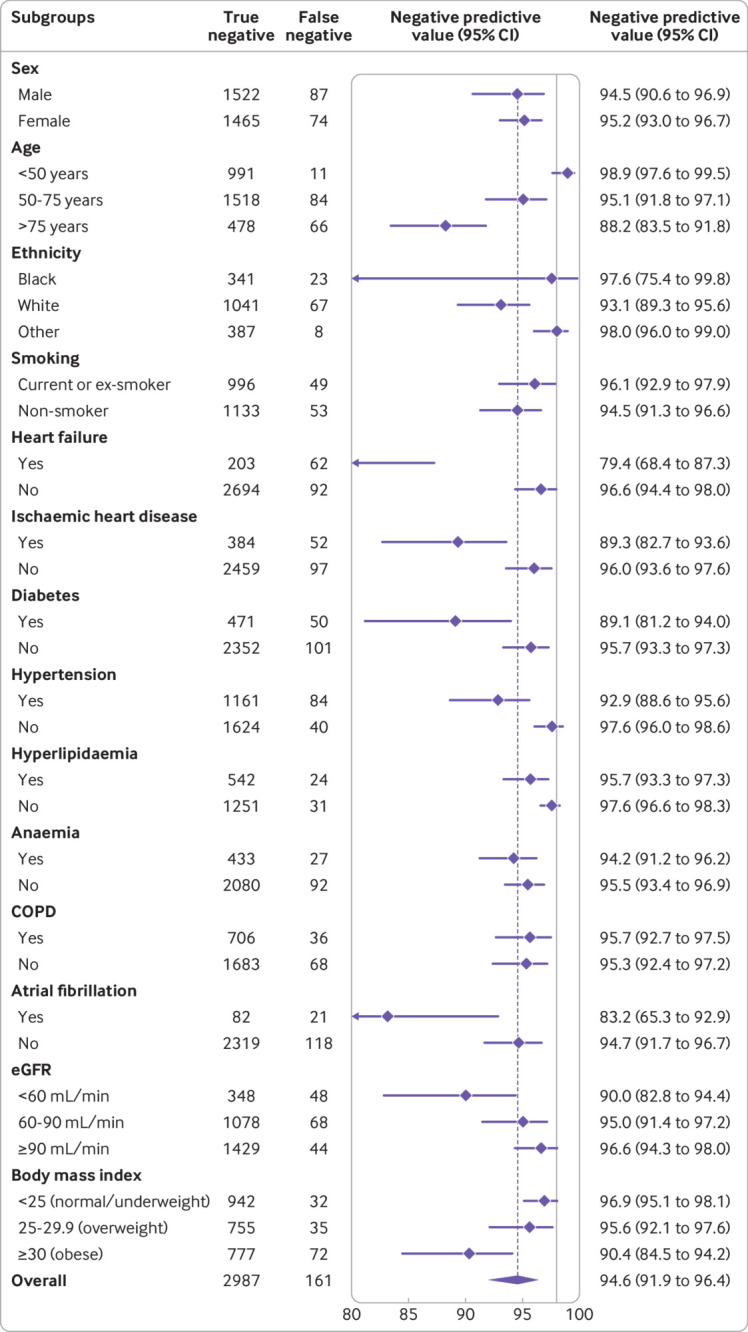
Diagnostic performance of guideline recommended N-terminal pro-B-type natriuretic peptide thresholds across patient subgroups: negative predictive value of threshold of 300 pg/mL. COPD=chronic obstructive pulmonary disease; eGFR=estimated glomerular filtration rate

**Fig 3:**
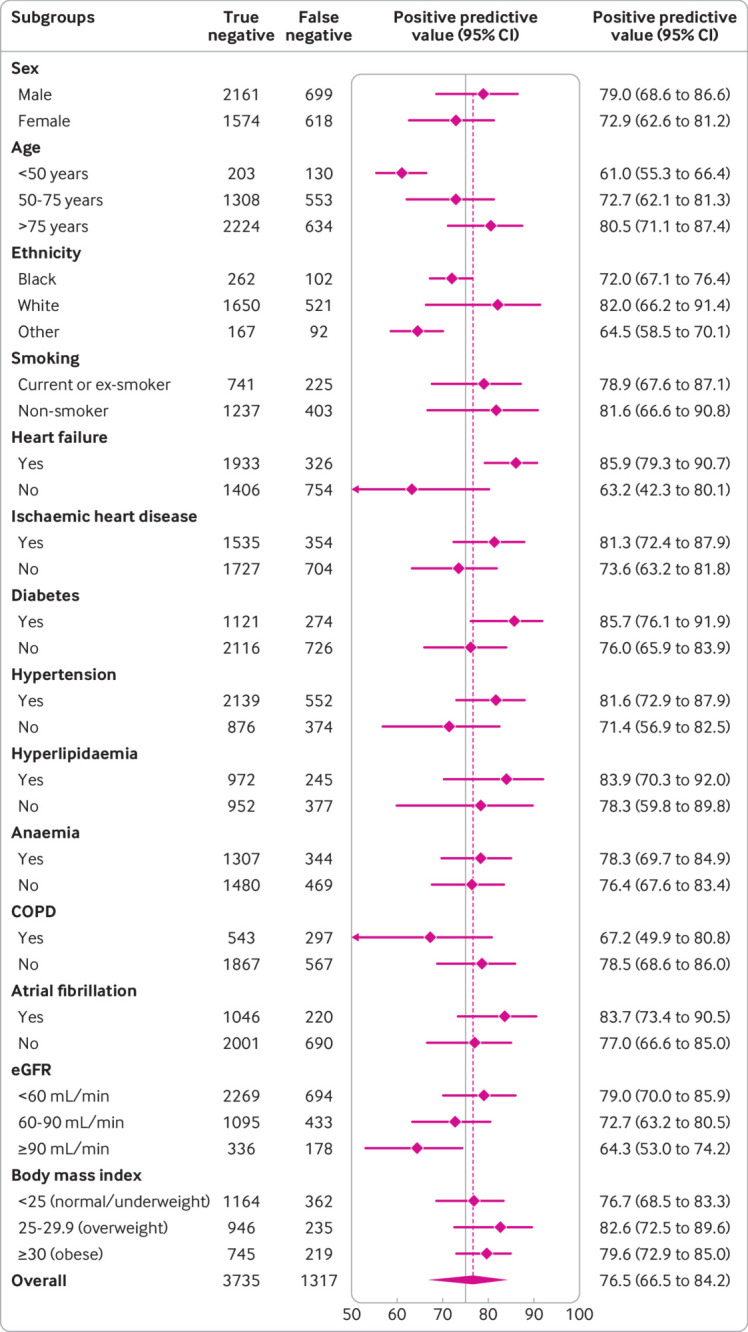
Diagnostic performance of guideline recommended NT-proBNP thresholds across patient subgroups: positive predictive value of age specific thresholds across patient subgroups (450, 900, and 1800 pg/mL for <50, 50-75, and >75 years, respectively). COPD=chronic obstructive pulmonary disease; eGFR=estimated glomerular filtration rate

Pooled meta-estimates of negative predictive value, sensitivity, positive predictive value, and specificity of NT-proBNP for the overall population at the guideline recommended rule-out threshold of 300 pg/mL were 94.6% (95% confidence interval 91.9% to 96.4%), 96.8% (94.6% to 98.1%), 62.9% (51.3% to 73.3%), and 49.3% (35.4% to 63.4%), respectively (fig 1; supplementary table D). Overall, 30.4% (3148/10 369) of patients had NT-proBNP concentrations below 300 pg/mL. However, marked heterogeneity existed across patient subgroups and studies (fig 2; fig 3; supplementary figures C and D). Negative predictive value was lower in patients ≥75 years (88.2%, 83.5% to 91.8%) and in those with previous heart failure (79.4%, 68.4% to 87.3%) and obesity (90.4%, 84.5% to 94.2%).

Pooled meta-estimates of the positive predictive value for age specific NT-proBNP rule-in thresholds of 450, 900 and 1800 pg/mL were 61.0% (55.3% to 66.4%), 73.5% (62.3% to 82.3%), and 80.2% (70.9% to 87.1%), respectively (table 2). Corresponding specificities were 87.8% (79.5% to 93.0%), 81.1% (72.6% to 87.5%), and 73.1% (65.2% to 79.8%). Overall, 48.7% (5052/10 369) of patients with suspected acute heart failure had NT-proBNP above these age specific thresholds. Positive predictive values of the age specific rule-in thresholds were higher than the uniform 300 pg/mL threshold in subgroups, although heterogeneity by age group, renal function, and prevalence of acute heart failure was present (supplementary figures E-I).

**Table 2:** Diagnostic performance of age specific thresholds of N-terminal pro-B-type natriuretic peptide (NT-proBNP) for acute heart failure

Age groups	NT-proBNP threshold (pg/mL)	True positive	False positive	True negative	False negative	Prevalence of acute heart failure (%)	NPV (95% CI)	PPV (95% CI)	Sensitivity (95% CI)	Specificity (95% CI)
<50 years	450	203	130	1025	19	16.1	98.4 (96.2 to 99.3)	61.0 (55.3 to 66.4)	91.4 (87.0 to 94.5)	87.8 (79.5 to 93.0)
50-75 years	900	1407	575	2121	267	38.3	88.3 (82.9 to 92.2)	73.5 (62.3 to 82.3)	83.2 (76.0 to 88.6)	81.1 (72.6 to 87.5)
>75 years	1800	2135	621	1348	518	57.4	72.2 (63.4 to 79.7)	80.2 (70.9 to 87.1)	79.3 (74.2 to 83.5)	73.1 (65.2 to 79.8)
All	300	4388	2833	2987	161	43.9	94.6 (91.9 to 96.4)	62.9 (51.3 to 73.3)	96.8 (94.6 to 98.1)	49.3 (35.4 to 63.4)

CI=confidence interval; NPV=negative predictive value; PPV=positive predictive value.

Overall, we identified seven studies as having a high risk of bias (supplementary table A). In sensitivity analyses restricted to studies in which the adjudication of acute heart failure was blinded to NT-proBNP concentrations and those with a low risk of bias, diagnostic performance of the guideline recommended and age specific NT-proBNP thresholds remained unchanged (supplementary tables E and F).

### Optimised NT-proBNP thresholds

An NT-proBNP threshold of 100 pg/mL achieved our optimal rule-out criteria with a pooled negative predictive value of 97.8% (95.8% to 98.8%) and sensitivity of 99.3% (98.5% to 99.7%) (supplementary table D). However, only 17.9% (1851/10 369) of patients had NT-proBNP concentrations below 100 pg/mL and the negative predictive value remained lower in older patients and those with previous history of heart failure, ischaemic heart disease, and impaired renal function (supplementary figure J). Similarly, a NT-proBNP threshold of 1000 pg/mL achieved our optimal rule-in criteria with a positive predictive value of 74.9% (64.4% to 83.2%) and specificity of 76.1% (65.6% to 84.2%), although performance was also lower within patient subgroups, particularly those without previous heart failure (positive predictive value 62%, 41% to 79%) (supplementary table D; supplementary figure K).

### CoDE-HF score

The extreme gradient boosting (XGBoost) model and generalised linear mixed model were the best performing models (area under curve in the overall training cohort of 0.925 (95% confidence interval 0.919 to 0.932) and 0.931 (0.925 to 0.937), respectively) (supplementary text 2). Although the performance of XGBoost was similar to the generalised linear mixed model, a key advantage of XGBoost is its ability to compute a score despite missing values. This is an important functionality that we wished to build into the CoDE-HF decision support tool to facilitate its implementation in clinical practice, so we selected the XGBoost model as the final model for CoDE-HF.

**Fig 4:**
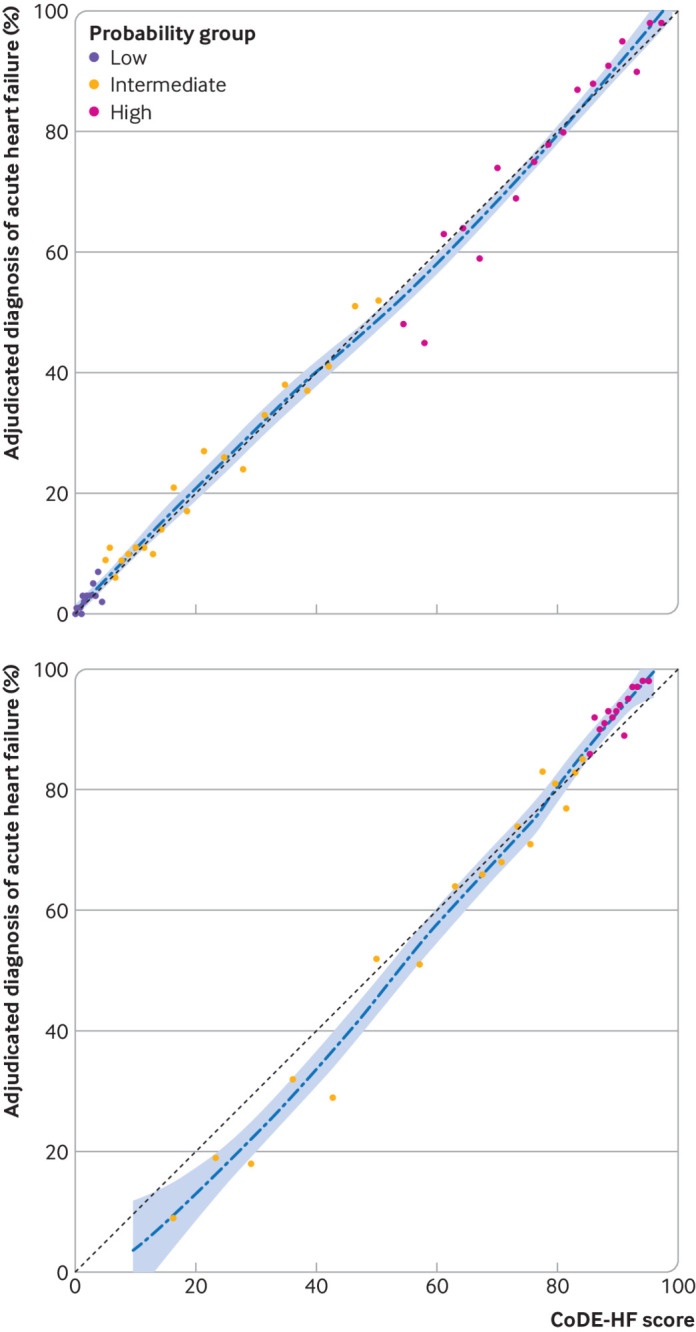
Calibration of Collaboration for the Diagnosis and Evaluation of Heart Failure (CoDE-HF) score with observed proportion of patients with acute heart failure. Dashed line represents perfect calibration. Each point represents 100 patients. Top: calibration of CoDE-HF in patients with no previous heart failure. Bottom: calibration of CoDE-HF in patients with previous heart failure

**Fig 5:**
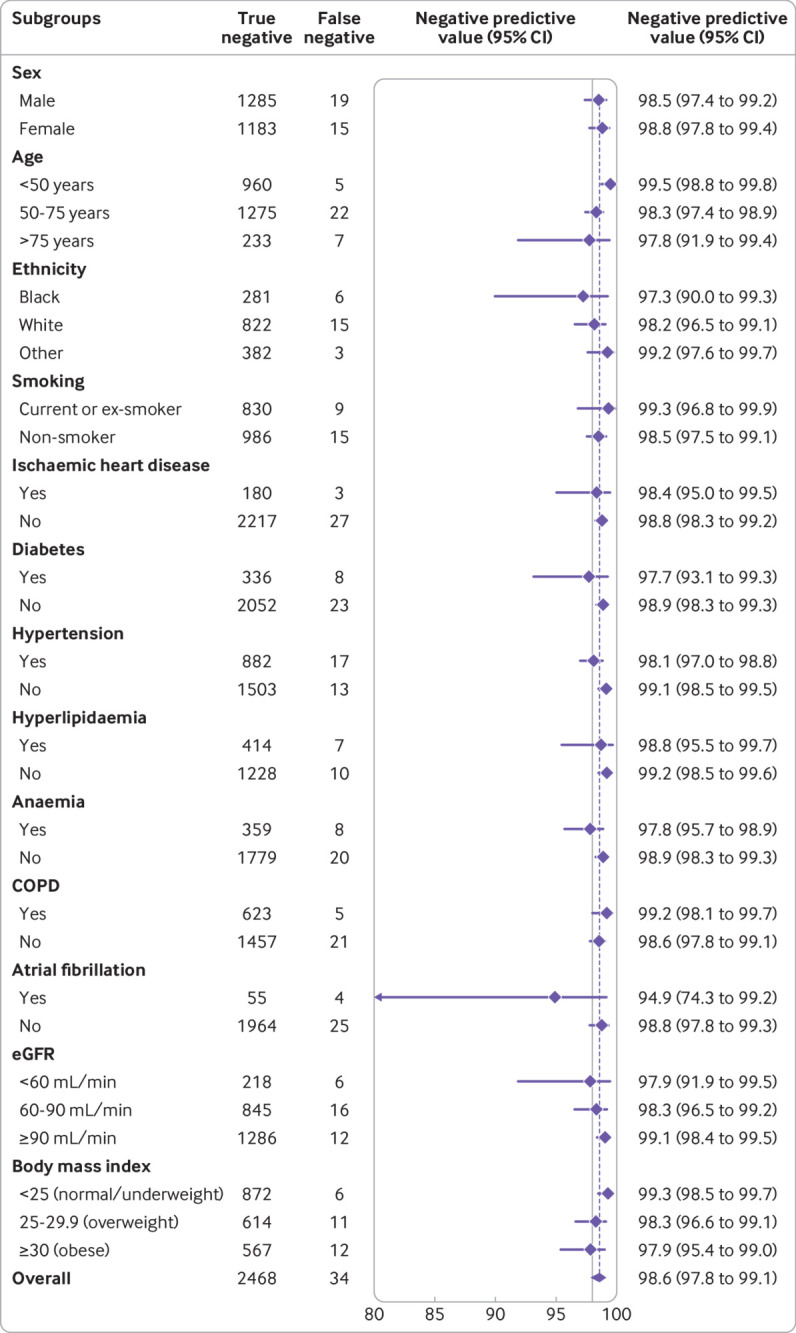
Diagnostic performance of Collaboration for the Diagnosis and Evaluation of Heart Failure (CoDE-HF) score across patient subgroups: negative predictive value of CoDE-HF rule-out score of 4.7 in patients without previous heart failure across patient subgroups. CoDE-HF incorporates N-terminal pro-B-type natriuretic peptide concentrations as continuous measure and predefined simple objective clinical variables (age, estimated glomerular filtration rate (eGFR), haemoglobin, body mass index, heart rate, blood pressure, peripheral oedema, chronic obstructive pulmonary disease (COPD), and ischaemic heart disease) to provide individualised assessment of likelihood of diagnosis of acute heart failure

**Fig 6:**
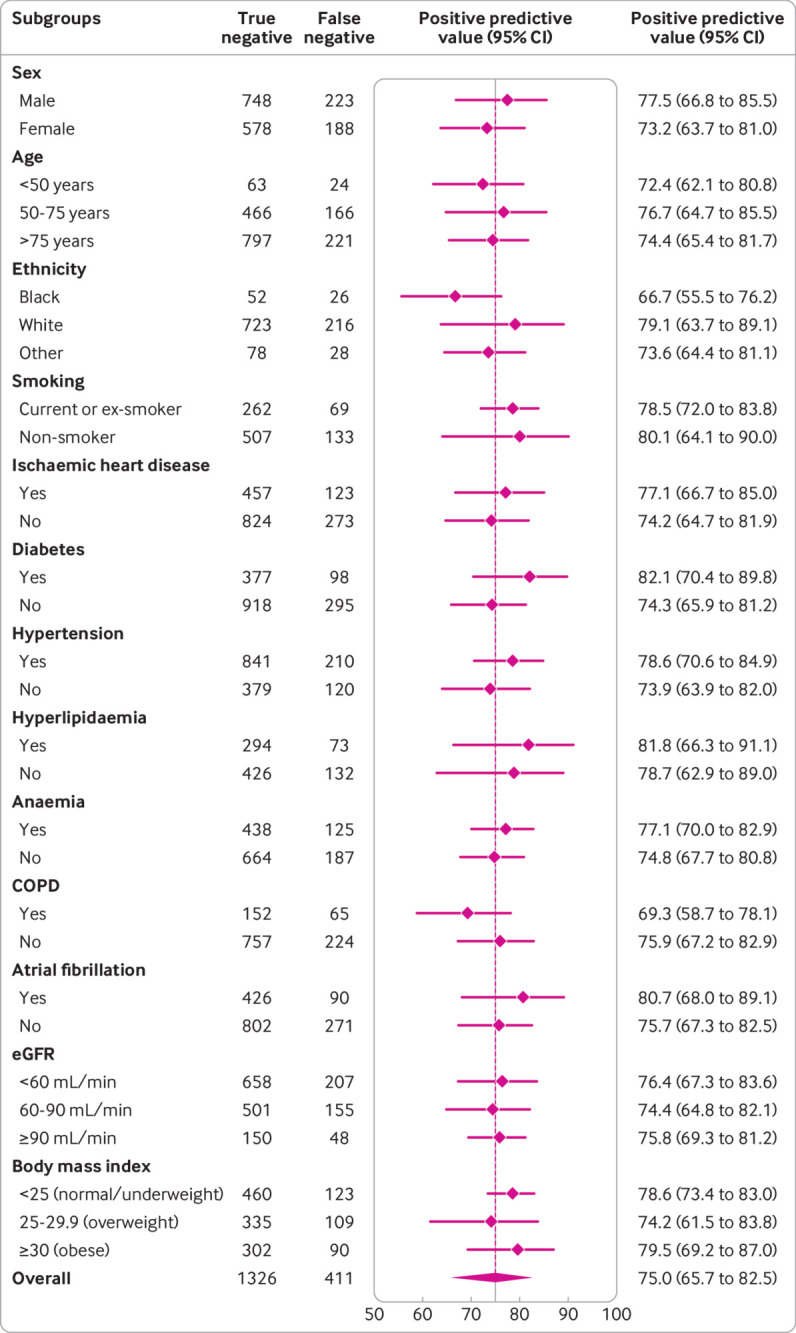
Diagnostic performance of Collaboration for the Diagnosis and Evaluation of Heart Failure (CoDE-HF) score across patient subgroups: positive predictive value of CoDE-HF rule-in score of 51.2 in patients without previous heart failure across patient subgroups. CoDE-HF incorporates NT-proBNP concentrations as continuous measure and predefined simple objective clinical variables (age, estimated glomerular filtration rate (eGFR), haemoglobin, body mass index, heart rate, blood pressure, peripheral oedema, chronic obstructive pulmonary disease (COPD), and ischaemic heart disease) to provide individualised assessment of likelihood of diagnosis of acute heart failure

**Fig 7:**
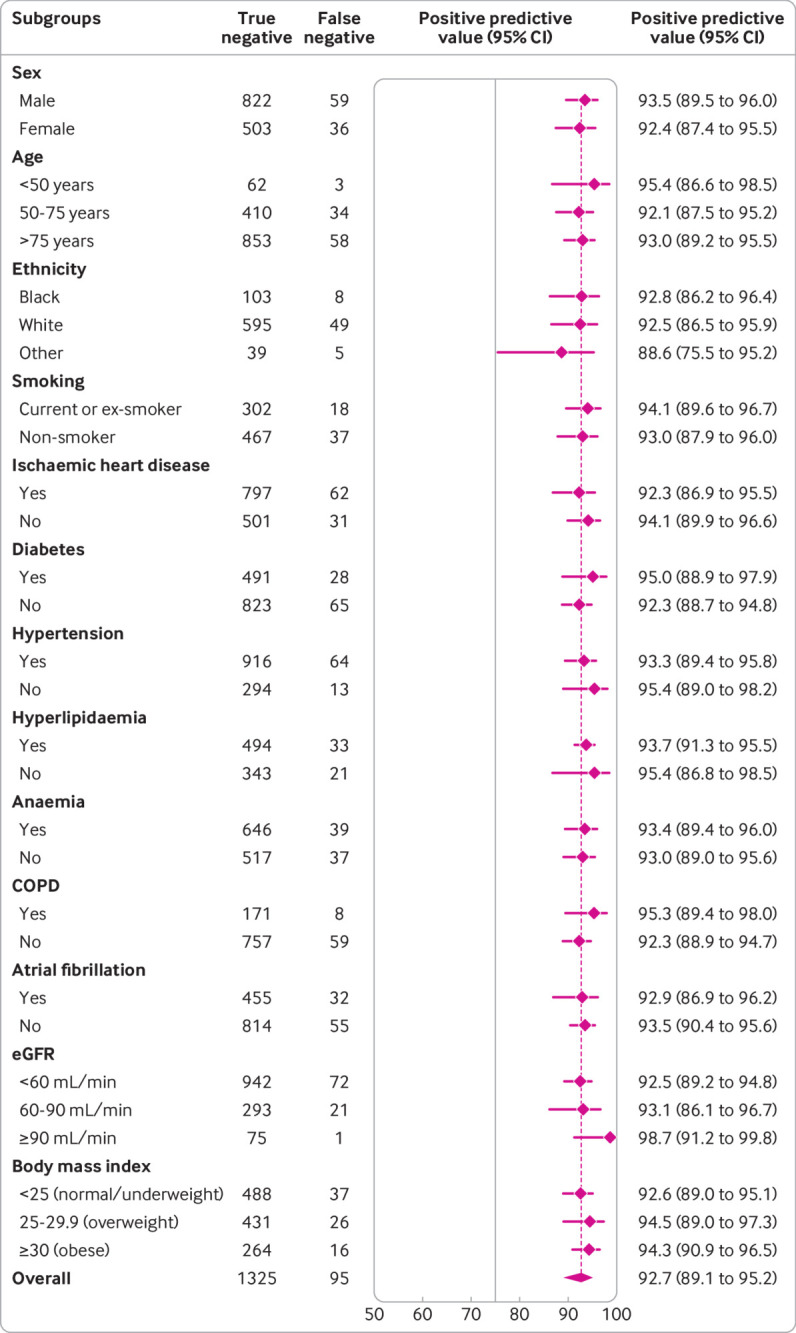
Diagnostic performance of Collaboration for the Diagnosis and Evaluation of Heart Failure (CoDE-HF) score across patient subgroups: positive predictive value of CoDE-HF rule-in score of 84.5 in patients with previous heart failure across patient subgroups. CoDE-HF incorporates NT-proBNP concentrations as continuous measure and predefined simple objective clinical variables (age, estimated glomerular filtration rate (eGFR), haemoglobin, body mass index, heart rate, blood pressure, peripheral oedema, chronic obstructive pulmonary disease (COPD), and ischaemic heart disease) to provide individualised assessment of likelihood of diagnosis of acute heart failure

**Fig 8:**
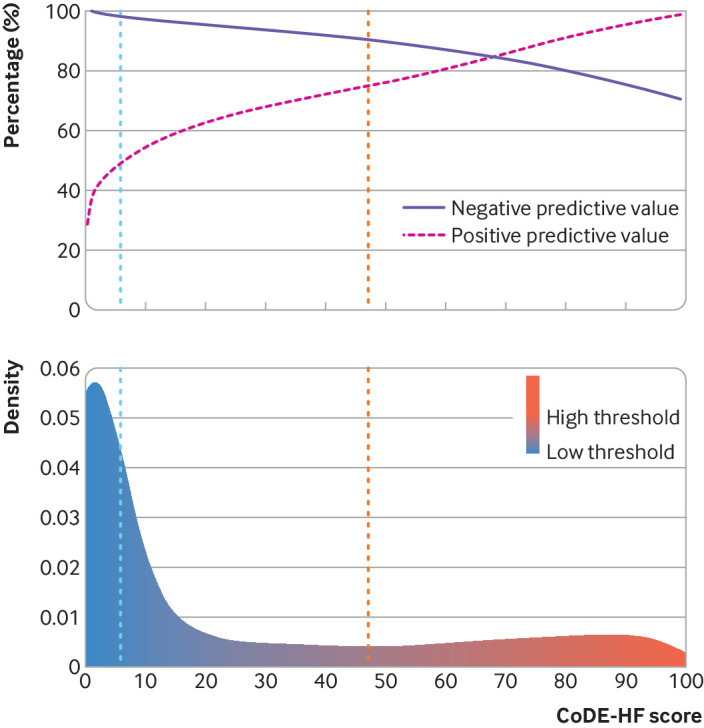
Diagnostic performance of Collaboration for the Diagnosis and Evaluation of Heart Failure (CoDE-HF) score in patients without previous heart failure. Top: negative and positive predictive values of CoDE-HF scores. Blue vertical dashed line represents target rule-out score of 4.7. Red vertical dashed line represents target rule-in score of 51.2. Bottom: density plot of CoDE-HF score in patients without previous heart failure. Target rule-out and rule-in scores identify 40.3% of patients as low probability and 28.0% as high probability respectively

CoDE-HF was well calibrated with excellent discrimination in patients with and without previous heart failure (area under the receiver operator curve 0.846 (0.830 to 0.862) and 0.925 (0.919 to 0.932) and Brier scores of 0.130 and 0.099, respectively) (fig 4; supplementary figure L). A CoDE-HF score of 4.7 achieved a negative predictive value of 98.6% (97.8% to 99.1%) and sensitivity of 98.1% (96.9% to 98.9%) (supplementary table G), and a score of 51.2 achieved a positive predictive value of 75.0% (65.7% to 82.5%) and a specificity of 92.2% (87.5% to 95.2%) in patients without previous heart failure. These rule-in and rule-out scores had similar diagnostic performance across all subgroups (fig 5; fig 6; fig 7). If these scores were applied in patients with suspected acute heart failure, CoDE-HF would identify 40.3% (2502/6208) at low probability (<4.7) and 28.0% (1737/6208) at high probability (≥51.2) of acute heart failure. In patients with previous heart failure, no score achieved our target rule-out criteria in the training cohort. A CoDE-HF score of 84.5 achieved a positive predictive value of 92.7% (89.1% to 95.2%) and specificity of 90.2% (84.0% to 94.1%). This score would identify 45.5% (1420/3119) of patients as having a high probability of acute heart failure (fig 8). In a decision curve analysis, CoDE-HF had superior net benefit compared with the NT-proBNP alone across all threshold probabilities (supplementary figure M). The performance of CoDE-HF was marginally attenuated when trained without past medical history (area under the receiver operator curve of 0.922 (0.916 to 0.929) and 0.841 (0.825 to 0.857) in patients without and with previous heart failure). Internal-external cross validation showed good performance across cohorts for both models (supplementary figure N).

**Fig 9:**
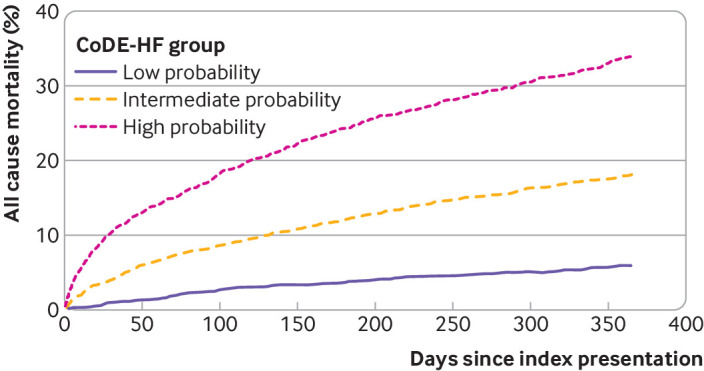
Cumulative incidence of all cause mortality stratified by Collaboration for the Diagnosis and Evaluation of Heart Failure (CoDE-HF) probability group

Patients who were identified as low probability by CoDE-HF had a substantially lower rate of all cause and cardiovascular mortality at 30 days and one year compared with those who were identified as intermediate and high probability (30 day all cause mortality: 1.0% versus 4.0% and 10.4%, respectively; one year all cause mortality: 5.9% versus 17.8% and 33.4%, respectively; 30 day cardiovascular mortality: 0.2% versus 0.8% and 4.1%; one year cardiovascular mortality: 1.4% versus 3.4% and 16.3%, respectively) (fig 9). In patients with NT-proBNP concentrations <300 pg/mL compared with those ≥300 pg/mL, the all cause mortality rates were 0.8% versus 7.6% at 30 days and 5.9% versus 26.6% at one year, respectively, and the cardiovascular mortality rates were 0.1% versus 2.6% at 30 days and 1.3% versus 10.2% at one year, respectively (supplementary table H; supplementary figure O).

## Discussion

We did a meta-analysis of individual patient level data to evaluate the diagnostic performance of NT-proBNP thresholds in more than 10 000 patients with suspected acute heart failure recruited in 14 prospective studies from 13 countries, and we developed and externally validated a decision support tool that uses NT-proBNP as a continuous measure. We report several important findings. Firstly, the guideline recommended threshold to rule out acute heart failure was heterogeneous across important patient subgroups.^3^ Whereas performance was good in the overall population and in several subgroups such as younger patients and women, the negative predictive value was substantially lower in older patients and in those with obesity or previous heart failure, for whom the false negative rates were between one in 10 and one in five. Secondly, age stratified thresholds performed well to rule in the diagnosis of acute heart failure. However, the positive predictive value was lower in younger patients. Thirdly, although our optimised NT-proBNP thresholds of 100 pg/mL to rule out and 1000 pg/mL to rule in acute heart failure had excellent negative and positive predictive values in the overall population, performance was lower in older patients and in those with previous heart failure and obesity. Finally, we developed and validated a decision support tool, the CoDE-HF score, which had excellent diagnostic performance across all patient subgroups. This decision support tool ruled in and ruled out acute heart failure more accurately than any approach using NT-proBNP thresholds alone.

### Strengths of study

To our knowledge, this is the largest study evaluating the diagnostic performance of NT-proBNP for acute heart failure to date. All included studies were prospective, and the final diagnosis was adjudicated by a panel of clinicians using all available information. Importantly, the availability of individual patient level data across a large study population allowed a robust evaluation of diagnostic performance across a full range of possible NT-proBNP thresholds within patient subgroups and the development and validation of a novel diagnostic score.

### Comparison with other studies

Most national and international guidelines recommend the use of an NT-proBNP threshold of 300 pg/mL to rule out acute heart failure,^5^ ^8^ on the basis of evidence from multiple previous studies,^3^ ^4^ ^41^ ^42^ which reported a negative predictive value of 98% at this threshold. However, these studies were unable to evaluate the diagnostic performance within important patient subgroups. Our study, which included three times as many patients as a previous study level meta-analysis,^3^ showed a lower overall negative predictive value at the 300 pg/mL threshold, with a pooled meta-estimate of 94.6%. More importantly, the negative predictive value was markedly lower in key subgroups, such as older patients and those with previous heart failure, ischaemic heart disease, and obesity. Moreover, nearly 70% of all patients had an NT-proBNP concentration above the 300 pg/mL threshold, highlighting the limitation of using a single threshold in practice. Although a lower threshold of 100 pg/mL achieved an overall negative predictive value of 98%, performance was poorer within important patient subgroups. Furthermore, the age specific and optimised thresholds to rule in acute heart failure had heterogeneous performance within patient subgroups, particularly in those without previous heart failure. This heterogeneity in diagnostic performance is particularly concerning as our patient populations are ageing and living with more comorbidities. This raises the question as to whether clinical guidelines should continue to recommend use of uniform thresholds when NT-proBNP is influenced by many risk factors and comorbidities.

To improve the clinical utility of NT-proBNP, we developed and externally validated a clinical decision support tool, the CoDE-HF score. This score incorporates NT-proBNP as a continuous measure together with simple, objective clinical variables to provide an individualised assessment of the likelihood of the diagnosis of acute heart failure. We showed that the diagnostic performance of the CoDE-HF score was robust across patient subgroups. CoDE-HF was able to rule out and rule in the diagnosis of acute heart failure in a larger proportion of patients compared with using optimised NT-proBNP thresholds alone. Furthermore, in our decision curve analysis, we found that CoDE-HF had a higher net benefit than NT-proBNP alone across the full range of threshold probabilities. We believe that this finding is intuitive given that NT-proBNP is a continuous marker of risk and concentrations are influenced by other patient related factors, such as body mass index, age, and renal function.^43^ ^44^ ^45^ Although these proportions were calculated on the basis of pre-specified performance criteria, we acknowledge that these targets may not be universally supported and different healthcare settings may have different tolerances for risk. The advantage of using a decision support tool, such as CoDE-HF, is that clinicians or institutions have the option to select the diagnostic performance criteria used to guide local decisions depending on their priorities and the availability of echocardiography or heart failure specialists.

### Implications for practice and future research

We anticipate that CoDE-HF, our novel decision support tool, has the potential to improve the triage of patients with suspected acute heart failure presenting to multiple medical specialties and to transform their care by facilitating more accurate diagnosis. Previous studies have showed that prompt and accurate delivery of evidence based treatments for patients with acute heart failure can lead to a substantial reduction in mortality and length of hospital stay, whereas delays are associated with worse outcomes.^46^ Furthermore, CoDE-HF uses routinely collected variables and therefore can be embedded within the clinical workflow as part of the triage pathway in the emergency department to facilitate more efficient assessment. Currently, the vast majority of patients with suspected acute heart failure undergo echocardiography during the course of their hospital admission to guide their care, but only a proportion of these patients ultimately have the diagnosis.^2^ Echocardiography is a relatively time consuming and resource intensive specialist investigation. We anticipate that use of CoDE-HF to guide more accurate and judicious use of specialist services such as echocardiography could lead to significant cost and efficiency savings for healthcare systems. Additionally, cost savings could also be made by triaging patients at low risk to outpatient care. A prospective study is now needed to evaluate the clinical and cost effectiveness of different CoDE-HF decision thresholds in clinical practice.

### Limitations of study

We acknowledge several limitations. Firstly, we were able to obtain individual patient level data for 14 of 30 studies that met our eligibility criteria, so the introduction of selection bias is a possibility. Nevertheless, the eligible studies that were not included had similar prevalence of acute heart failure, publication dates, and geographical coverage, and the population had similar demographic and clinical characteristics to those that were included. Secondly, in combining information from multiple studies, data were missing for some variables in several studies. To maximise the use of information, we have used a hierarchical multiple imputation method. Thirdly, we did not have consistently recorded data from the electrocardiogram and chest radiograph to enable inclusion of these data in our model. The interpretation of NT-proBNP in patients with suspected acute heart failure should be made in conjunction with these investigations,^47^ and future studies are needed to determine whether approaches that integrate these investigations can improve the performance of CoDE-HF. Fourthly, not all studies have adjudicated the diagnosis blinded to the results of NT-proBNP testing. In our sensitivity analysis, diagnostic performance was unchanged when we excluded the two studies in which adjudication was unblinded. Fifthly, the adjudicated diagnosis of acute heart failure did not differentiate between heart failure with reduced ejection fraction and heart failure with preserved ejection fraction.^48^ The increasing prevalence of heart failure with preserved ejection fraction in older patients may explain some of the heterogeneity observed across age groups, but current guidelines recommend the same NT-ProBNP threshold for both heart failure with reduced ejection fraction and heart failure with preserved ejection fraction.^5^ ^8^ Sixthly, although most of the studies included consecutive patients with acute dyspnoea, the prevalence of acute heart failure was high and some selection bias may have existed. However, the performance of guideline recommended and age specific NT-proBNP thresholds were unchanged in a sensitivity analysis excluding studies at high risk of bias. Finally, acute heart failure is a clinical syndrome and the diagnostic adjudication itself has inherent uncertainty and variability across studies. This uncertainty may be greater among older adults, which may, in part, explain some of the observed heterogeneity in diagnostic performance.

### Conclusions

We have shown that the diagnostic performance of guideline recommended NT-proBNP thresholds for acute heart failure varies across important patient subgroups. We developed and validated the CoDE-HF score, which combines NT-pro-BNP as a continuous measure with clinical variables by using statistical modelling to determine the probability of acute heart failure for individual patients. This decision support tool accurately ruled in and ruled out acute heart failure and performed consistently across all subgroups. Prospective studies are now needed to evaluate the effect of implementing this decision support tool on healthcare resource utilisation and patients’ outcomes.

## What is already known on this topic

The diagnosis of acute heart failure can be challenging because patients often present with non-specific symptoms
Most national and international guidelines recommend N-terminal pro-B-type natriuretic peptide (NT-proBNP) testing to aid in the diagnosis of acute heart failure
NT-proBNP testing has not been universally implemented owing to concerns about diagnostic performance in clinically important patient subgroups

## What this study adds

The guideline recommended NT-proBNP thresholds for acute heart failure had relatively poor diagnostic performance in important patient subgroups
A validated decision support tool that uses statistical modelling to combine NT-pro-BNP as a continuous measure with clinical variables has been developed
This tool ruled in and ruled out acute heart failure more accurately than did any approach using NT-proBNP thresholds alone and performed consistently across all subgroups

## Data Availability

The R code and anonymised data used to develop and validate the CoDE-HF score can be made available to researchers on request to the corresponding author.

## Web extra

Extra material supplied by authors

Web appendix: Supplementary materials

## Notes

Contributors: KKL, JJ, and NLM conceived the study and its design. KKL, MA, and NLM did the systematic review. KKL, MA, CCG, CD, GM, JHWR, LG, MM, MBe, MBo, PN, PB, TM, AMR, CM, and JJ acquired the data. KKL and DD did the analysis. KKL, DD, MA, AGJ, AMR, JJVM, CM, JJ, and NLM interpreted the data. KKL, DD, and NLM drafted the manuscript. All authors revised the manuscript critically for important intellectual content and approved the final version to be published. KKL, DD, and NLM are the guarantors. The corresponding author attests that all listed authors meet authorship criteria and that no others meeting the criteria have been omitted.

The lead author (KKL) affirms that the manuscript is an honest, accurate, and transparent account of the study being reported; that no important aspects of the study have been omitted; and that any discrepancies from the study as planned (and, if relevant, registered) have been explained.

Dissemination to participants and related patient and public communities: Findings of this study will be shared with clinicians and patients through national and international cardiology conferences and through social media platforms. A plain language summary will also be disseminated through a press release.

Provenance and peer review: Not commissioned; externally peer reviewed.
