# Supplementary material for: Development and validation of a decision support tool for the diagnosis of acute heart failure: systematic review, meta-analysis, and modelling study
Source: BMJ. 2022 Jun 13;377:e068424. doi: 10.1136/bmj-2021-068424 (PMC9189738; doi:10.1136/bmj-2021-068424)
Supplement: Supplementary file 1 — Web appendix: Supplementary materials [file leek068424.ww1.pdf]

SUPPLEMENT

Development and validation of a decision-support tool for the diagnosis of acute heart failure

Kuan Ken Lee, MD,[1]* Dimitrios Doudesis, MSc,[1, 2]* Mohamed Anwar, MD,[1]* Federica Astengo, BSc,[1] Camille Chenevier-Gobeaux, PharmD PhD,[3] Yann-Erick Claessens, MD,[4] Desiree Wussler, MD,[5, 6] Nikola Kozhuharov, MD,[5, 7] Ivo Strebel, PhD,[5] Zaid Sabti, MD,[5] Christopher deFilippi, MD,[8] Stephen Seliger, MD,[9] Gordon Moe, MD,[10] Carlos Fernando, MD,[10] Antoni Bayes-Genis, MD,[11] Roland RJ van Kimmenade, MD,[12] Yigal Pinto, MD,[13] Hanna K Gaggin, MD,[14, 15] Jan C Wiemer, PhD,[16] Martin Möckel, MD,[17] Joost HW Rutten, MD,[18] Anton H. van den Meiracker, MD,[19] Luna Gargani, MD,[20] Nicola R Pugliese, MD,[21] Christopher Pemberton, PhD,[22] Irwani Ibrahim, MD,[23] Alfons Gegenhuber, MD,[24] Thomas Mueller, MD,[25] Michael Neumaier, MD,[26] Michael Behnes, MD,[27] Ibrahim Akin, MD,[27] Michele Bombelli, MD,[28] Guido Grassi, MD,[29] Peiman Nazerian, MD,[30] Giovanni Albano, MD,[30] Philipp Bahrmann, MD,[31] David E Newby, MD,[1] Alan G Japp, MD,[1] Athanasios Tsanas, PhD,[2] Anoop SV Shah, MD,[1, 32] A Mark Richards, MD,[22, 33] John JV McMurray, MD,[34] Christian Mueller, MD,[5] James L Januzzi, MD,[14, 15] Nicholas L Mills, MD[1, 2]

on behalf of the CoDE-HF investigators[†]

*Contributed equally

[1] British Heart Foundation (BHF) Centre for Cardiovascular Science, University of Edinburgh, Edinburgh, UK.
[2] Usher Institute, University of Edinburgh, Edinburgh, UK.
[3] Department of Biochemistry, Cochin Hospital, Assistance Publique-Hopitaux de Paris, Paris, France.
[4] Department of Emergency Medicine, Princess Grace Hospital Center, Monaco, Principalty of Monaco.
[5] Cardiovascular Research Institute of Basel, Department of Cardiology, University Hospital Basel, Basel, Switzerland.
[6] Department of Internal Medicine, University Hospital Basel, University of Basel, Switzerland.
[7] Liverpool Heart and Chest Hospital, Liverpool, United Kingdom.
[8] Inova Heart and Vascular Institute, Falls Church, Virginia, USA
[9] Division of Nephrology, University of Maryland School of Medicine, Baltimore, USA.
[10] University of Toronto, St Michael's Hospital, Toronto, Ontario, Canada.
[11] Heart Institute, Hospital Universitari Germans Trias i Pujol, Badalona, CIBERCV, Spain.
[12] Department of Cardiology, Radboud University Medical Center, Nijmegen, The Netherlands
[13] University of Amsterdam, Amsterdam, The Netherlands.
[14] Harvard Medical School, Boston, Massachusetts, USA.
[15] Division of Cardiology, Massachusetts General Hospital, Boston, Massachusetts, USA.
[16] BRAHMS, Thermo Fisher Scientific, Hennigsdorf, Germany.
[17] Department of Emergency and Acute Medicine with Chest Pain Units, Charité – Universitätsmedizin Berlin, Campus Mitte and Virchow, Berlin, Germany.
[18] Department of Internal Medicine, Radboud University Medical Center, Nijmegen, The Netherlands.
[19] Department of Internal Medicine, Division of Pharmacology and Vascular Medicine, Erasmus Medical Center, Rotterdam, The Netherlands.
[20] Department of Surgical, Medical and Molecular Pathology and Critical Care Medicine, University of Pisa, Pisa, Italy
[21] Department of Clinical and Experimental Medicine, University of Pisa, Pisa, Italy.
[22] Christchurch Heart Institute, University of Otago, Christchurch, New Zealand.
[23] Emergency Medicine Department, National University Hospital, Singapore.
[24] Department of Internal Medicine, Krankenhaus Bad Ischl, Bad Ischl, Austria.
[25] Department of Laboratory Medicine, Hospital Voecklabruck, Austria.
[26] Institute for Clinical Chemistry, University Medical Centre Mannheim, Faculty of Medicine Mannheim, University of Heidelberg, Mannheim, Germany.
[27] First Department of Medicine, University Medical Centre Mannheim, Faculty of Medicine Mannheim, University of Heidelberg, Mannheim, Germany.
[28] University of Milan Bicocca, ASST-Brianza, Pio XI Hospital of Desio, Internal Medicine, Desio (MB), Italy.
[29] Clinica Medica, University Milan Bicocca, Milan, Italy
[30] Department of Emergency Medicine, Azienda Ospedaliero-Universitaria Careggi, Florence, Italy
[31] Department of Internal Medicine III, Division of Cardiology, University Hospital of Heidelberg, Ruprecht-Karls University Heidelberg, Heidelberg, Germany.
[32] London School of Hygiene and Tropical Medicine, London, UK
[33] Cardiovascular Research Institute, National University Heart Centre Singapore, Singapore
[34] British Heart Foundation Cardiovascular Research Centre, University of Glasgow, Glasgow, UK
[†] Listed in the Supplementary Appendix

Corresponding Author:
Professor Nicholas L Mills
BHF/University Centre for Cardiovascular Science
The University of Edinburgh
Edinburgh EH16 4SA
United Kingdom
Telephone: 0044 131 242 6515
E-mail: nick.mills@ed.ac.uk

Supplementary Texts: 2
Supplementary Tables: 8
Supplementary Figures: 15

Table of Contents

I. LIST OF INVESTIGATORS. 4

II. SUPPLEMENTARY TEXT 6

SUPPLEMENTARY TEXT 1. LITERATURE SEARCH STRATEGY. 6
SUPPLEMENTARY TEXT 2. STATISTICAL MODELS FOR THE DEVELOPMENT OF THE CODE-HF SCORE 9

III. SUPPLEMENTARY TABLES 18

SUPPLEMENTARY TABLE A. CHARACTERISTICS OF EACH INCLUDED STUDY. 18
SUPPLEMENTARY TABLE B. BASELINE CHARACTERISTICS OF PATIENTS WITHIN EACH STUDY... 23
SUPPLEMENTARY TABLE C. BASELINE CHARACTERISTICS OF STUDY PATIENTS STRATIFIED BY PRIOR HISTORY OF HEART FAILURE. 26
SUPPLEMENTARY TABLE D. DIAGNOSTIC PERFORMANCE OF NT-PROBNP FOR ACUTE HEART FAILURE. 27
SUPPLEMENTARY TABLE E. SENSITIVITY ANALYSIS OF THE DIAGNOSTIC PERFORMANCE OF GUIDELINE-RECOMMENDED AND AGE-SPECIFIC THRESHOLDS OF NT-PROBNP FOR ACUTE HEART FAILURE IN STUDIES WHERE THE REFERENCE STANDARD WAS BLINDED TO NT-PROBNP CONCENTRATION. 29
SUPPLEMENTARY TABLE F. SENSITIVITY ANALYSIS OF THE DIAGNOSTIC PERFORMANCE OF GUIDELINE-RECOMMENDED AND AGE-SPECIFIC THRESHOLDS OF NT-PROBNP FOR ACUTE HEART FAILURE IN STUDIES WITH LOW RISK OF BIAS IN PATIENT SELECTION. 30
SUPPLEMENTARY TABLE G. DIAGNOSTIC PERFORMANCE OF RULE-IN AND RULE-OUT THRESHOLDS AND CODE-HF SCORES FOR ACUTE HEART FAILURE. 31
SUPPLEMENTARY TABLE H. MORTALITY IN THE POOL COHORT STRATIFIED BY CODE-HF PROBABILITY GROUP AND NT-PROBNP THRESHOLD OF 300 PG/ML 33

IV. SUPPLEMENTARY FIGURES 34

SUPPLEMENTARY FIGURE A. PROPORTION OF MISSING DATA IN THE VARIABLES INCLUDED IN THE DIAGNOSTIC MODELS ACROSS STUDIES. 34
SUPPLEMENTARY FIGURE B. FLOW DIAGRAM OF STUDY PARTICIPANTS. 35
SUPPLEMENTARY FIGURE C. NEGATIVE PREDICTIVE VALUE OF NT-PROBNP AT THE 300 PG/ML THRESHOLD ACROSS COHORTS. 36
SUPPLEMENTARY FIGURE D. META-REGRESSION OF THE NEGATIVE PREDICTIVE VALUE OF NT-PROBNP AT THE THRESHOLD OF 300 PG/ML BY PREVALENCE OF ACUTE HEART FAILURE. 37
SUPPLEMENTARY FIGURE E. POSITIVE PREDICTIVE VALUE OF THE 300 PG/ML NT-PROBNP THRESHOLD ACROSS PATIENT SUBGROUPS. 38
SUPPLEMENTARY FIGURE F. POSITIVE PREDICTIVE VALUE OF THE NT-PROBNP THRESHOLD OF 300 PG/ML ACROSS COHORTS. 39
SUPPLEMENTARY FIGURE G. META-REGRESSION OF POSITIVE PREDICTIVE VALUE OF THE 300 PG/ML NT-PROBNP THRESHOLD BY PREVALENCE OF ACUTE HEART FAILURE. 40
SUPPLEMENTARY FIGURE |H. POSITIVE PREDICTIVE VALUE OF AGE-SPECIFIC THRESHOLDS OF NT-PROBNP ACROSS COHORTS. 41
SUPPLEMENTARY FIGURE I. META-REGRESSION OF POSITIVE PREDICTIVE VALUE OF AGE-SPECIFIC THRESHOLDS OF NT-PROBNP BY PREVALENCE OF ACUTE HEART FAILURE. 42

SUPPLEMENTARY FIGURE J. NEGATIVE PREDICTIVE VALUE OF THE NT-PROBNP THRESHOLD OF 100 PG/ML ACROSS PATIENT SUBGROUPS. 43
SUPPLEMENTARY FIGURE K. POSITIVE PREDICTIVE VALUE OF THE NT-PROBNP THRESHOLD OF 1000 PG/ML ACROSS PATIENT SUBGROUPS. 44
SUPPLEMENTARY FIGURE L. DISCRIMINATION OF THE GUIDELINE-RECOMMENDED NT-PROBNP THRESHOLDS AND CODE-HF SCORE 45
SUPPLEMENTARY FIGURE M. DECISION CURVE ANALYSIS FOR CODE-HF VERSUS NT-PROBNP ALONE 47
SUPPLEMENTARY FIGURE N. INTERNAL-EXTERNAL CROSS-VALIDATION OF CODE-HF 49
SUPPLEMENTARY FIGURE O. CUMULATIVE INCIDENCE OF ALL-CAUSE MORTALITY STRATIFIED BY NT-PROBNP CONCENTRATION 51

V. CHECKLISTS 52

PRISMA-IPD CHECKLIST OF ITEMS TO INCLUDE WHEN REPORTING A SYSTEMATIC REVIEW AND META-ANALYSIS OF INDIVIDUAL PARTICIPANT DATA (IPD) 52
TRIPOD CHECKLIST: PREDICTION MODEL DEVELOPMENT AND VALIDATION 56

REFERENCES 58

I. List of Investigators.

CoDE-HF Investigators

Chief Investigator: Prof Nicholas L Mills, MD[1,2]

Research team: Dr Kuan Ken Lee, MD,[1] Mr Dimitrios Doudesis, MSc,[1,2] Dr Mohammed Anwar, MD,[1] Ms Federica Astengo, MD BSc,[1] Prof David E Newby, MD,[1] Dr Alan Japp, MD,[1] Dr Athanasios Tsanas, PhD,[1] Dr Anoop SV Shah, MD.[1,3]

Collaborators: Prof Adam Singer, MD,[4] Prof Judd Hollander, MD,[5] Dr Camille Chenevier-Gobeaux, PharmD PhD,[6] Prof Yann-Erick Claessens, MD,[7] Prof Christian Mueller, MD,[8] Dr Desiree Wussler, MD,[8,9] Dr Nikola Kozhuharov, MD,[8,10] Dr Ivo Strebel, PhD,[8] Dr Zaid Sabti, MD,[8] Dr Christopher deFilippi, MD,[11] Dr Stephen Seliger, MD,[12] Prof Gordon Moe, MD,[13] Dr Carlos Fernando, MD,[13] Prof Humberto Villacorta, MD PhD,[14] Prof Evandro Tinoco Mesquita, MD PhD,[14] Prof Antoni Bayes-Genis, MD,[15] Dr Roland RJ van Kimmenade, MD,[16] Prof Yigal Pinto, MD,[17] Dr Hannah K Gaggin, MD MPH,[18,19] Dr Jan C Wiemer, PhD,[20] Prof Martin Möckel, MD,[21] Dr Joel Coste, MD PhD,[22] Prof Patrick Jourdain, MD,[23] Dr Joost HW Rutten, MD,[24] Dr Anton H. van den Meiracker, MD,[25] Dr Kimiaki Komukai, MD PhD,[26] Prof Michihiro Yoshimura, MD PhD,[26] Dr Luna Gargani, MD,[27] Dr Nicola R Pugliese, MD,[28] Dr Christopher Pemberton, PhD,[29] Dr Irwani Ibrahim, MD,[30] Dr Alfons Gegenhuber, MD,[31] Dr Thomas Mueller, MD,[32] Dr Michael Neumaier, MD,[33] Prof Michael Behnes, MD,[34] Prof Ibrahim Akin, MD,[34] Prof Michele Bombelli, MD,[35] Prof Guido Grassi, MD,[35] Prof Olivier Hanon, MD PhD,[36] Dr Jean-Sébastien Vidal, MD PhD,[36] Dr Peiman Nazerian, MD,[37] Dr Giovanni Albano, MD,[37] Prof Peter Cameron, MD,[38] Dr Louisa Lam, PhD,[39] Dr Philipp Bahrmann, MD MHBA,[40] Prof Ben Freedman, MB PhD,[41] Dr Tommy Chung, PhD,[42] Dr Sean P Collins, MD,[43] Prof Christopher John Lindsell, PhD.[44]

Writing group: Dr Kuan Ken Lee, MD,[1] Mr Dimitrios Doudesis, MSc,[1,2] Dr Mohammed Anwar, MD,[1] Dr Alan Japp, MD,[1] Prof A Mark Richards, MD,[29,45] Prof John JV McMurray, MD,[46] Prof Christian Mueller, MD,[8] Prof James L Januzzi, MD,[18,19] Prof Nicholas L Mills, MD.[1,2]

Affiliations:

[1] British Heart Foundation (BHF) Centre for Cardiovascular Science, University of Edinburgh, Edinburgh, UK.
[2] Usher Institute, University of Edinburgh, Edinburgh, UK.
[3] London School of Hygiene and Tropical Medicine, London, UK.
[4] Department of Emergency Medicine, Stony Brook University, New York, USA.

[5] Department of Emergency Medicine, Thomas Jefferson University, Philadelphia, USA.
[6] Department of Biochemistry, Cochin Hospital, Assistance Publique-Hopitaux de Paris, Paris, France.
[7] Department of Emergency Medicine, Princess Grace Hospital Center, Monaco, Principalty of Monaco.
[8] Cardiovascular Research Institute of Basel, Department of Cardiology, University Hospital Basel, Basel, Switzerland.
[9] Department of Internal Medicine, University Hospital Basel, University of Basel, Switzerland.
[10] Liverpool Heart and Chest Hospital, Liverpool, United Kingdom.
[11] Inova Heart and Vascular Institute, Falls Church, Virginia, USA.
[12] Division of Nephrology, University of Maryland School of Medicine, Baltimore, USA.
[13] University of Toronto, St Michael's Hospital, Toronto, Ontario, Canada.
[14] Fluminense Federal University, Niteroi, Rio de Janeiro State, Brazil.
[15] Heart Institute, Hospital Universitari Germans Trias i Pujol, Badalona, CIBERCV, Spain.
[16] Department of Cardiology, Radboud University Medical Center, Nijmegen, The Netherlands.
[17] University of Amsterdam, Amsterdam, The Netherlands.
[18] Harvard Medical School, Boston, Massachusetts, USA.
[19] Division of Cardiology, Massachusetts General Hospital, Boston, Massachusetts, USA.
[20] BRAHMS, Thermo Fisher Scientific, Hennigsdorf, Germany.
[21] Department of Emergency and Acute Medicine with Chest Pain Units, Charité – Universitätsmedizin Berlin, Campus Mitte and Virchow, Berlin, Germany.
[22] Cochin Hospital, Paris, France.
[23] Cardiology Department, AP-HP, Paris-Saclay University, Paris, France.
[24] Department of Internal Medicine, Radboud University Medical Center, Nijmegen, The Netherlands.
[25] Department of Internal Medicine, Division of Pharmacology and Vascular Medicine, Erasmus Medical Center, Rotterdam, The Netherlands.
[26] Division of Cardiology, The Jikei University Kashiwa Hospital, Kashiwa, Japan.
[27] Department of Surgical, Medical and Molecular Pathology and Critical Care Medicine, University of Pisa, Pisa, Italy
[28] Department of Clinical and Experimental Medicine, University of Pisa, Pisa, Italy.
[29] Christchurch Heart Institute, University of Otago, Christchurch, New Zealand.
[30] Emergency Medicine Department, National University Hospital, Singapore.
[31] Department of Internal Medicine, Krankenhaus Bad Ischl, Bad Ischl, Austria.
[32] Department of Laboratory Medicine, Hospital Voecklabruck, Austria.
[33] Institute for Clinical Chemistry, University Medical Centre Mannheim, Faculty of Medicine Mannheim, University of Heidelberg, Mannheim, Germany.
[34] First Department of Medicine, University Medical Centre Mannheim, Faculty of Medicine Mannheim, University of Heidelberg, Mannheim, Germany.
[35] University of Milano – Bicocca, Milan, Italy.
[36] Department of Geriatrics, Broca Hospital, Assistance Publique-Hôpitaux de Paris, Paris, France.
[37] Department of Emergency Medicine, Azienda Ospedaliero-Universitaria Careggi, Florence, Italy.
[38] Public Health and Preventive Medicine, Monash University, Australia.
[39] School of Health, Federation University, Australia.
[40] Department of Internal Medicine III, Division of Cardiology, University Hospital of Heidelberg, Ruprecht-Karls University Heidelberg, Heidelberg, Germany.
[41] Heart Research Institute, University of Sydney, Sydney, Australia.
[42] Concord Repatriation General Hospital, NSW, Australia.
[43] Department of Emergency Medicine, Vanderbilt University Medical Center, Nashville, Tennessee, USA.
[44] Department of Biostatistics, Vanderbilt University Medical Center, Nashville, Tennessee, USA.
[45] Cardiovascular Research Institute, National University Heart Centre Singapore, Singapore.
[46] BHF Cardiovascular Research Centre, University of Glasgow, Glasgow, UK.

II. Supplementary Text

Supplementary Text 1. Literature Search Strategy.

Embase

1 *heart failure/ or acute heart failure/ or *cardiogenic shock/ or *diastolic dysfunction/ or *forward heart failure/ or *high output heart failure/ or *systolic dysfunction/
2 *Congestive Cardiomyopathy/ or exp *Congestive Heart Failure/
3 exp *Heart Ventricle Failure/
4 ((heart or cardiac or myocardial) adj2 (failure or decompensation)).ti.
5 ((congestive or acute or decompensat$) adj2 " heart failure").ti,ab.
6 ((dilated or congestive) adj2 cardiomyopath$).ti.
7 "cardiogenic shock".ti.
8 ((ventricular or ventricle$) adj2 (failure or insufficien$ or dysfunction$)).ti.
9 (("left ventricular" or "left ventricle") adj2 (failure or insufficien$ or dysfunction$)).ti,ab.
10 lvsd.ti,ab.
11 or/1-10
12 letter.pt. or letter/
13 note.pt.
14 editorial.pt.
15 case report/ or case study/
16 (letter or comment*).ti.
17 or/12-16
18 randomized controlled trial/ or random*.ti,ab.
19 17 not 18
20 animal/ not human/
21 nonhuman/
22 exp Animal Experiment/
23 exp Experimental Animal/
24 animal model/
25 exp Rodent/
26 (rat or rats or mouse or mice).ti.
27 or/19-26
28 11 not 27
29 limit 28 to english language
30 exp *natriuretic factor/
31 (natriuretic adj2 peptide$).ti,ab.
32 (natriuretic adj2 factor*).ti,ab.
33 (BNP or ANP or pro-BNP or pro-ANP or pro BNP or pro ANP).ti,ab.
34 or/30-33
35 29 and 33

Medline

1 exp Heart Failure/
2 Cardiomyopathy, Dilated/
3 Shock, Cardiogenic/
4 exp Ventricular Dysfunction/
5 Cardiac Output, Low/
6 ((heart or cardiac or myocardial) adj2 (failure or decompensation)).ti.
7 ((congestive or acute or decompensat$) adj2 " heart failure").ti,ab.
8 ((dilated or congestive) adj2 cardiomyopath$).ti.
9 "cardiogenic shock".ti.
10 ((ventricular or ventricle$) adj2 (failure or insufficien$ or dysfunction$)).ti.
11 (("left ventricular" or "left ventricle") adj2 (failure or insufficien$ or dysfunction$)).ti,ab.
12 lvsd.ti,ab.
13 or/1-12
14 letter/
15 editorial/
16 news/
17 exp historical article/
18 Anecdotes as Topic/
19 comment/
20 case report/
21 (letter or comment*).ti.
22 or/14-21
23 randomized controlled trial/ or random*.ti,ab.
24 22 not 23
25 animals/ not humans/
26 exp Animals, Laboratory/
27 exp Animal Experimentation/
28 exp Models, Animal/
29 exp Rodentia/
30 (rat or rats or mouse or mice).ti.
31 or/24-30
32 13 not 31
33 limit 32 to english language
34 exp *Natriuretic Peptides/
35 (natriuretic adj2 peptide$).ti,ab.
36 (natriuretic adj2 factor*).ti,ab.
37 (BNP or ANP or pro-BNP or pro-ANP or pro BNP or pro ANP).ti,ab.
38 34 or 35 or 36 or 37
39 33 and 38

Cochrane central register of controlled trials

#1 MeSH descriptor: [Heart Failure] explode all trees

#2 MeSH descriptor: [Cardiomyopathy, Dilated] explode all trees

#3 MeSH descriptor: [Shock, Cardiogenic] explode all trees

#4 MeSH descriptor: [Ventricular Dysfunction] explode all trees

#5 MeSH descriptor: [Cardiac Output, Low] explode all trees

#6 (heart or cardiac or myocardial) near/2 (failure or decompensation):ti

#7 ((congestive or acute or decompensat*) near/2 "heart failure"):ti,ab

#8 (dilated or congestive) near/2 cardiomyopath*:ti

#9 cardiogenic shock:ti

#10 (ventricular or ventricle*) near/2 (failure or insufficien* or dysfunction*):ti

#11 (("left ventricle" or "left ventricular") near/2 (failure or insufficienc* or dysfunction*)):ti,ab

#12 lvsd:ti,ab

#13 MeSH descriptor: [Pulmonary Edema] this term only cardiogenic near/2 ("pulmonary edema" or "pulmonary oedema" or "lung edema" or "lung

#14 oedema"):ti,ab

#15 #1 or #2 or #3 or #4 or #5 or #6 or #7 or #8 or #9 or #10 or #11 or #12 or #13 or #14

#16 MeSH descriptor: [Natriuretic Peptides] explode all trees

#17 (natriuretic near/2 peptide*):ti,ab

#18 (natriuretic near/2 factor*):ti,ab

#19 (BNP or ANP or pro-BNP or pro-ANP or pro BNP or pro ANP):ti,ab

#20 #16 or #17 or #18 or #19

#21 #15 and #20

Supplementary Text 2. Statistical models for the development of the CoDE-HF score

S2.1 Extreme gradient boosting model

XGBoost is a supervised machine learning technique initially proposed by Chen and Guestrin.[1] In brief, gradient boosting employs an ensemble technique to iteratively improve model accuracy for regression and classification problems. This ensemble-based algorithm is achieved by creating sequential models, using decision trees as learners where subsequent models attempt to correct errors of the preceding models.[2 3] In the boosting method, individuals that were misclassified by the previous model are assigned a higher weight to increase their chance of being selected in subsequent models. Each model is subsequently fitted in a step-wise fashion to minimise loss function such as absolute error or squared error (the amount predicted values differ from the true values). XGBoost refers to the re-engineering of gradient boosting to significantly improve the speed of the algorithm by pushing the limits of computational resources. The output of the XGBoost model is a probability that is computed by performing an inverse-logit transformation of the sum of the weights of the terminal nodes of the trained model.

The mathematical formula for the gradient boosting model can be described as:

$$\hat{y}_i = \sum_{k=1}^{K} f_k\ (x_i), f_k \in F \tag{1}$$

where f is an function that map each variable vector x_i (x_i = { x_i, x_2, …, x_n }, i = 1, 2, N) to the outcome y_i, K is the number of Classification and Regression Trees (CART) and F is the space of function containing all CART.[5]

XGBoost optimises an objective function of the form:

$$Obj = \sum_{i=1}^{N} l(y_i, \hat{y}_i) + \sum_{k=1}^{K} \Omega(f_k) \tag{2}$$

Where the first term is a loss function, l, which evaluates how well the model fits the data by measuring the difference between the prediction $\hat{y}_i$ and the outcome y_i. The second term, the

regularization term, is used by XGBoost to avoid overfitting by penalizing the complexity of the model. Furthermore, to improve and fully leverage the advantages of XGBoost we tuned the hyper-parameters of the algorithm defined below through a grid search strategy using 10-fold cross-validation.

The hyper-parameter values for the model in patients without prior heart failure were: the number of iterations (trees) was set to 81, the learning rate (shrinkage parameter applied to each tree in the expansion) was set to 0.1, the interaction depth (maximum depth of each tree, expresses the highest level of variable interactions allowed) was set to 8, the minimum number of observations in the terminal nodes was set to 5.7, the fraction of the training set observations randomly selected for each subsequent tree was set to 0.55 and the fraction of variables randomly sampled for each tree was set to 0.84.

The hyper-parameter values for the model in patients with prior heart failure were: the number of iterations (trees) was set to 69, the learning rate (shrinkage parameter applied to each tree in the expansion) was set to 0.06, the interaction depth (maximum depth of each tree, expresses the highest level of variable interactions allowed) was set to 5, the minimum number of observations in the terminal nodes was set to 6.7, the fraction of the training set observations randomly selected for each subsequent tree was set to 0.73 and the fraction of variables randomly sampled for each tree was set to 0.62.

The algorithm was developed using the R package ‘xgboost’ (https://cran.r-project.org/web/packages/xgboost/).

S2.1.1 Relative feature importance plots

a) Relative feature importance plot for the model developed for patients without prior heart failure.

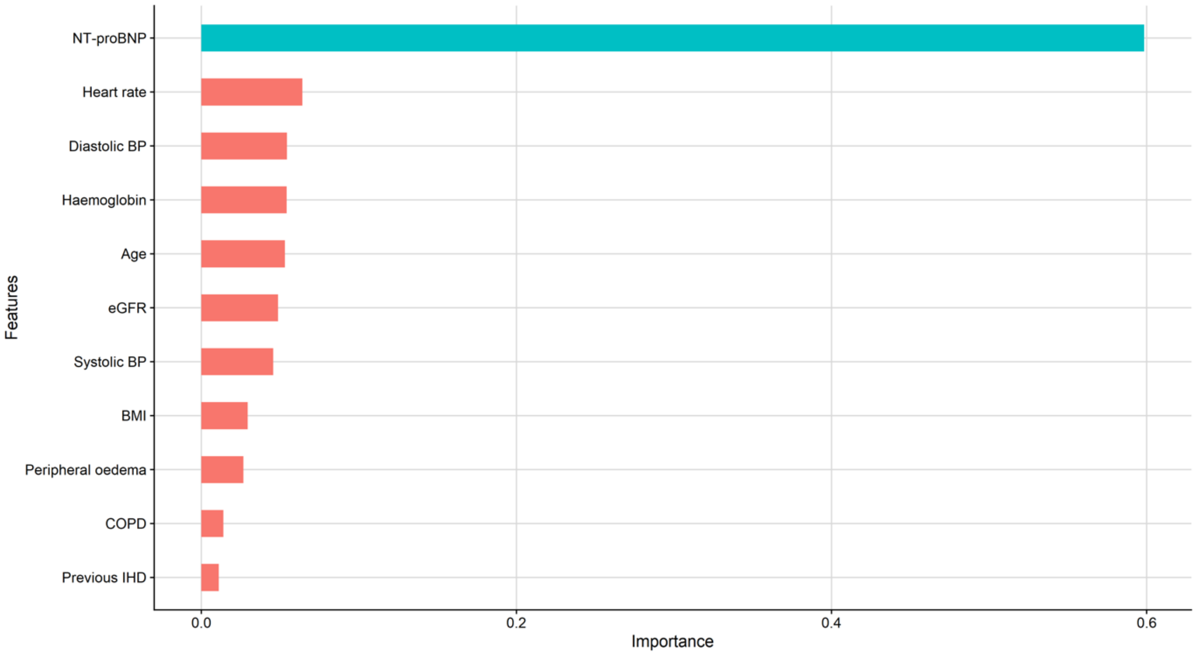

b) Relative feature importance plot for the model developed for patients with prior heart failure

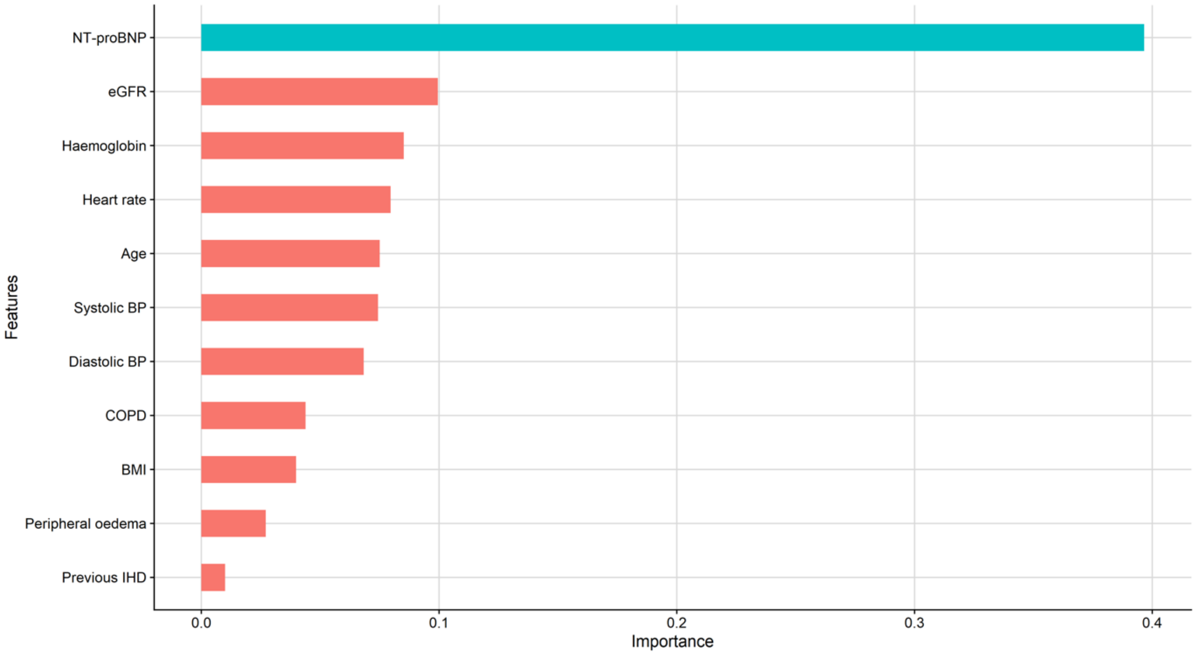

S2.2 Generalised linear mixed model

A study identifier was included as a random effects variable whilst all other variables (NT-proBNP, age, estimated glomerular filtration rate, hemoglobin, body mass index, heart rate, blood pressure, peripheral edema, prior history of heart failure, chronic obstructive pulmonary disease and ischemic heart disease) were fitted as fixed effects variables. Due to the positive-skew in NT-proBNP concentrations, we used a logarithmic transformation in the model. We further evaluated non-linear relationships between continuous variables and the diagnosis using multivariable fractional polynomial methods.[4] The model was developed using the R package 'lme4'(https://cran.r-project.org/web/packages/lme4).

.

S2.3 Naïve Bayes

Naive Bayes (NB)[5] , is a supervised machine learning algorithm based on Bayes' Theorem with an "naive" assumption of independence among features. In brief, a NB algorithm assumes no relationship between features. We used a kernel density estimation function to achieve higher accuracy levels. The algorithm was developed using the R package 'naivebayes'(https://cran.r-project.org/web/packages/naivebayes).

S2.4 Random forest

Random Forest (RF)[6], is a supervised machine learning algorithm. It is an ensemble technique that combines a large number of decision trees using a bagging approach to improve the overall performance. In brief, the bagging approach grows multiple classification trees in parallel where each tree gives a classification which are called votes. These votes are then aggregated to provide a more accurate and stable prediction.

We tuned the RF hyper-parameters during the development of this model through a grid search strategy using 10-fold cross-validation. The hyper-parameters tuned were the number of trees in the forest, the number of variables randomly sampled as candidates at each split, the maximum depth of the tree and the minimum number of samples required to split an internal node. The algorithm was developed using the R package 'randomForest'(https://cran.r-project.org/web/packages/randomForest).

A. Rule-out diagnostic performance

	NPV (95% CI)	Sensitivity (95% CI)	Proportion ruled out	AUC	Brier score
**Patients without prior heart failure**					
XGBoost (NT-proBNP only)	98.5 (97.5-99.1)	99.0 (97.8-99.5)	22.4 %	0.882 (0.873-0.891)	0.123
GLMM	98.6 (97.4-99.2)	98.1 (96.0-99.1)	41.1 %	0.931 (0.925-0.937)	0.094
NB	98.6 (97.2-99.3)	98.5 (96.9-99.3)	33.8 %	0.892 (0.884-0.900)	0.129
RF	98.6 (97.0-99.3)	98.2 (96.6-99.0)	38.4 %	0.914 (0.906-0.921)	0.108
XGBoost (all variables)	98.6 (97.8-99.1)	98.1 (96.9-98.9)	40.3 %	0.925 (0.919-0.932)	0.099
XGBoost (all variables except past medical history)	98.5 (97.7-99.0)	98.1 (96.8-98.9)	38.4%	0.922 (0.916-0.929)	0.100

B. Rule-in diagnostic performance

	PPV (95% CI)	Specificity (95% CI)	Proportion ruled in	AUC	Brier score
**Patients without prior heart failure**					
XGBoost (NT-proBNP only)	75.8 (53.9-89.3)	94.2 (88.9-97.0)	21.5 %	0.882 (0.873-0.891)	0.123
GLMM	75.1 (67.2-81.7)	91.0 (86.2-94.2)	30.1 %	0.931 (0.925-0.937)	0.094
NB	75.5 (54.6-88.7)	94.4 (90.5-96.8)	19.0 %	0.892 (0.884-0.900)	0.129
RF	75.2 (53.3-88.9)	92.4 (87.0-95.7)	26.7 %	0.914 (0.906-0.921)	0.108
XGBoost (all variables)	75.0 (65.7-82.5)	92.2 (87.5-95.2)	28.0 %	0.925 (0.919-0.932)	0.099
XGBoost (all variables except past medical history)	75.1 (65.4-82.7)	92.2 (87.5-95.3)	28.1 %	0.922 (0.916-0.929)	0.100
**Patients with prior heart failure**					
XGBoost (NT-proBNP only)	89.9 (82.7-94.3)	90.2 (86.0-93.3)	27.8 %	0.779 (0.759-0.798)	0.150
GLMM	93.2 (89.9-95.4)	90.1 (82.4-94.6)	47.5 %	0.860 (0.845-0.875)	0.123
NB	91.9 (86.7-95.2)	90.3 (87.2-92.7)	33.6 %	0.812 (0.794-0.829)	0.144
RF	93.5 (88.9-96.2)	90.4 (86.2-93.4)	41.3 %	0.830 (0.813-0.847)	0.137
XGBoost (all variables)	92.7 (89.1-95.2)	90.2 (84.0-94.1)	45.5 %	0.846 (0.830-0.862)	0.130
XGBoost (all variables except past medical history)	92.2 (86.8-95.5)	90.2 (83.7-94.3)	44.4 %	0.841 (0.825-0.857)	0.131

Abbreviations: GLMM=generalised linear mixed-model; NB=naïve bayes; RF=random forest; XGBoost=extreme gradient boosting.

A) Receiver operator curve for all statistical models in the patients without prior heart failure

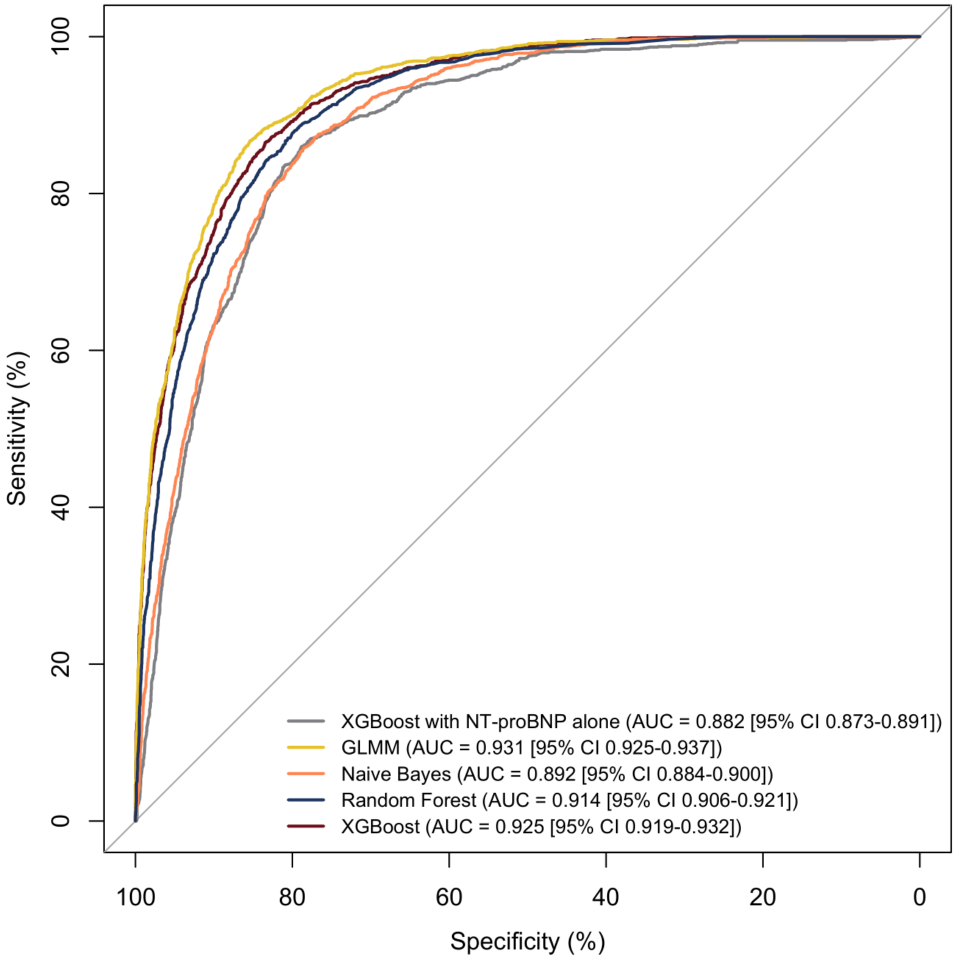

B) Receiver operator curve for all statistical models in the patients without prior heart failure

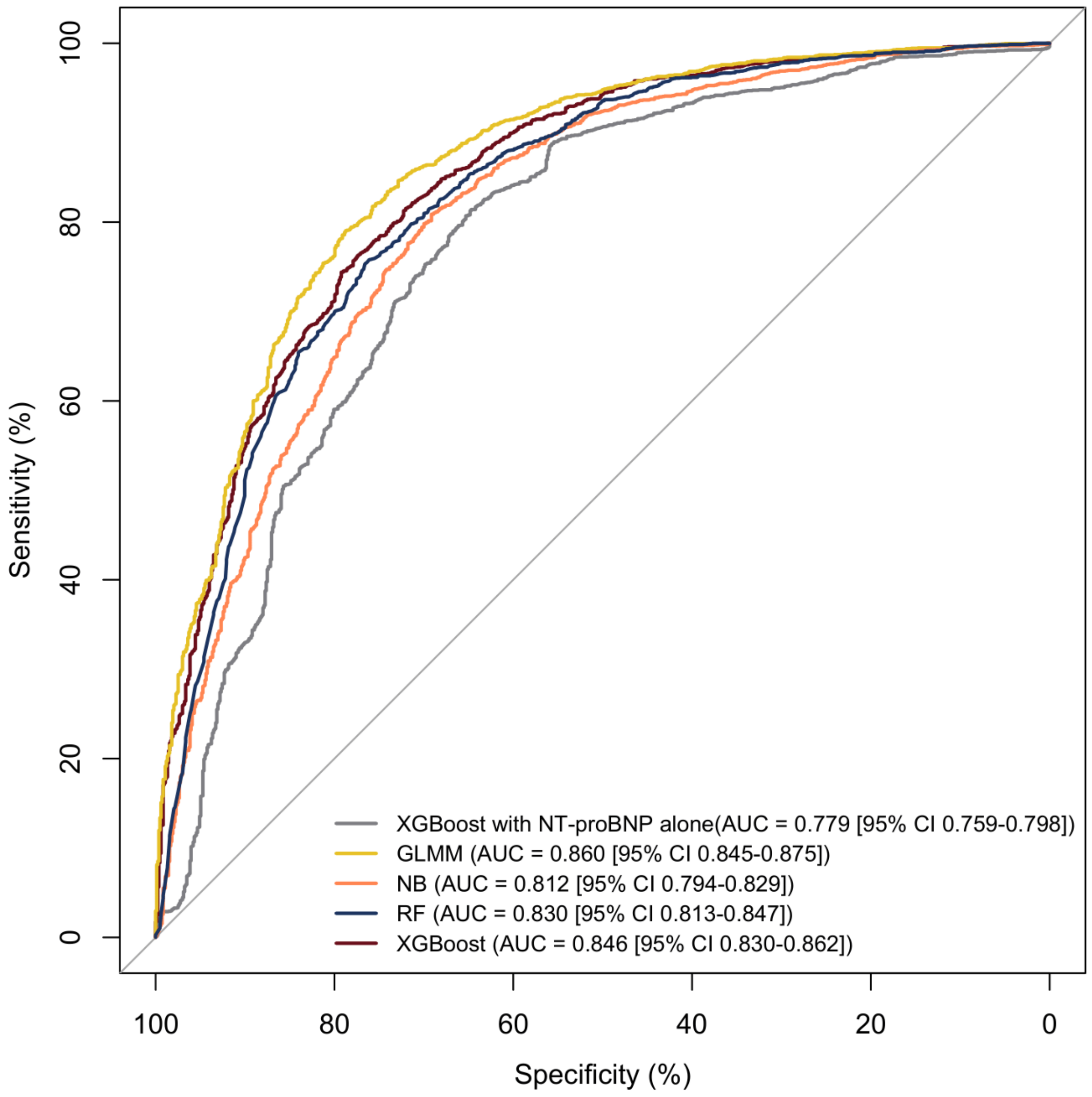

Whilst the performance of XGBoost was similar to the generalised linear mixed-model, a key advantage of XGBoost is its ability to compute a score despite missing values. This is a critical functionality for the application of the CoDE-HF decision-support tool in clinical practice because clinicians may not always have all information available to them during the initial clinical encounter in the Emergency Department.

III. Supplementary Tables

Supplementary Table A. Characteristics of each included study.

Author, year	NT-proBNP assay	Study design	Country	Cohort size	Setting
**Bahrmann et al, 2015**[7]	Roche	Prospective cohort	Germany	303	Emergency department
**Behnes et al, 2009**[8]	Dade Behring	Prospective cohort	Germany	401	Emergency department
**Bombelli et al, 2015**[9]	Roche	Prospective cohort	Italy	895	Emergency department
**Chenevier-Gobeaux et al, 2005**[10]	Roche	Prospective cohort	France	380	Emergency department
**deFilippi et al, 2007**[11]	Roche	Prospective cohort	USA	831	Emergency department
**Gargani et al, 2008**[12]	Roche	Prospective cohort	Italy	149	Cardiology and pulmonary hospital admissions
**Ibrahim et al, 2017**[13]	Roche	Prospective cohort	Singapore, New Zealand	1106	Emergency department
**Januzzi et al, 2006**[14]	Roche	Prospective cohort	New Zealand, Spain, USA	1256	Emergency department
**Maisel et al, 2010**[15]	Roche	Prospective cohort	USA, Germany, Switzerland, Italy, Greece, UK, Poland, New Zealand	1623	Emergency department
**Moe et al, 2007**[16]	Roche	Randomized controlled trial	Canada	500	Emergency department
**Mueller et al, 2005**[17]	Roche	Prospective cohort	Austria	251	Emergency department
**Nazerian et al, 2010**[18]	Roche	Prospective cohort	Italy	145	Emergency department
**Rutten et al, 2008**[19]	Roche	Randomized controlled trial	The Netherlands	476	Emergency department
**Wussler et al, 2019**[20]	Roche	Prospective cohort	Switzerland	2053	Emergency department

Author, year	Inclusion criteria	Exclusion criteria
**Bahrmann et al, 2015**[7]	All consecutive non-trauma patients aged ≥70 years who were admitted to the Emergency Department.	Patients with acute ST-elevation myocardial infarction, planned elective coronary revascularisation, hospitalization for unstable angina within the preceding 2 months, coronary-artery bypass grafting or percutaneous transluminal angioplasty within the preceding 3 months. Patients were also excluded if they had renal failure requiring dialysis, trauma with suspected myocardial contusion, life expectancy <6 months, or if they did not consent to providing a blood sample for use by the research team.
**Behnes et al, 2009**[8]	Consecutive patients presenting with symptoms of acute dyspnoea and/ or peripheral oedema in the Emergency Department.	Patients suffering from severe renal disease (defined as serum creatinine level greater than 2.8 mg/dl) or anemia (hemoglobin concentrations below 8.0 g/dl) were excluded. Further exclusion criteria were obvious traumatic causes of dyspnea, pregnancy, a status after immediate cardiopulmonary resuscitation, participation in another clinical trial and age under 18 years.
**Bombelli et al, 2015**[9]	Consecutive patients aged 80 years or more evaluated in the Emergency Department in whom NT-proBNP was measured.	
**Chenevier-Gobeaux et al, 2005**[10]	Consecutive patients presenting to the Emergency Department with dyspnoea.	
**deFilippi et al, 2007**[11]	Consecutive patients presenting to the Emergency Department with dyspnoea and who underwent measurement of a natriuretic peptide at presentation.	Patients younger than 18 years or in whom there was inadequate clinical information recorded to assess the aetiology of dyspnoea were excluded.
**Gargani et al, 2008**[12]	Patients with dyspnoea at admission as reported on the case history, had a venous blood sample taken for NT-proBNP analysis on the day of admission, underwent assessment for ultrasound lung comets performed within 4 h of the NT-proBNP measurement and did not receive diuretic therapy between the two measurements.	
**Ibrahim et al, 2017**[13]	Shortness of breath as the primary complaint triggering presentation to the Emergency Department.	Age under 21 years, shortness of breath related to trauma, and current renal haemodialysis were exclusion criteria.
**Januzzi et al, 2006**[14]	Dyspnoeic Emergency Department patients.	
**Maisel et al, 2010**[15]	Patients reporting shortness of breath as primary	Patients <18 years of age, unable to provide consent, had an acute

	complaint in the Emergency Department.	ST-segment elevation myocardial infarction, were receiving haemodialysis, or had renal failure.
**Moe et al, 2007**[16]	Consecutive patients >18 years of age presenting to the Emergency Department with dyspnoea of suspected cardiac origin.	Patients with advanced renal failure (serum creatinine >250 micromol/L), acute myocardial infarction, malignant disorders, and dyspnoea from clinically overt origins, including pneumothorax and chest wall trauma.
**Mueller et al, 2005**[17]	Consecutive patients presenting with dyspnoea as chief complaint to the Emergency Department.	Patients with ST elevation myocardial infarction, non-ST elevation myocardial infarction, or acute coronary syndrome troponin positive and trauma patients.
**Nazerian et al, 2010**[18]	Convenience sample of patients presenting to the Emergency Department with acute dyspnoea as the main symptom.	Patients with trauma, ST-elevation myocardial infarctions, or dyspnoea clearly caused by something other than heart failure, such as pneumothorax, were excluded. Patients were also excluded if they had received intravenous therapy in the Emergency Department before echocardiogram and NT-proBNP were performed. Patients who met the inclusion criteria were invited to participate in the study. Echocardiogram was performed in all patients who met the inclusion criteria. However, if the investigator judged that both left ventricular (LV) ejection fraction and pulsed Doppler analysis of mitral inflow were not interpretable due to a very poor acoustic window, the patient was excluded from the study because echocardiogram was not feasible.
**Rutten et al, 2008**[19]	Patients were eligible if they presented with acute dyspnoea as their most prominent complaint.	Patients with acute dyspnoea due to trauma or cardiogenic shock and patients with renal failure requiring haemodialysis or peritoneal dialysis were excluded.
**Wussler et al, 2019**[20]	Adult patients presenting with acute dyspnoea to the Emergency Department.	Patients with terminal kidney failure requiring haemodialysis were excluded.

Author, year	Diagnostic adjudication for acute heart failure	Risk of bias (QUADAS-2)
**Bahrmann et al, 2015**[7]	Independent adjudication by two cardiologists based on the definition of the ESC guideline. They reviewed all available medical records of the index hospital stay, including the clinical history findings from the physical examination, results of laboratory tests (excluding NT-proBNP), radiographic studies, ECG, and echocardiography.	Patient selection: high; index test: low; reference standard: low; flow and timing: low; Overall: high.
**Behnes et al, 2009**[8]	Retrospective review by a study physician who had unrestricted access to the records of the patients but was blinded to the results of NT-proBNP measurements.	Patient selection: low; index test: low; reference standard: low; flow and timing: low; Overall: low.
**Bombelli et al, 2015**[9]	Adjudication by clinician based on Framingham criteria.	Patient selection: high; index test: low; reference standard: low; flow and timing: low; Overall: high.
**Chenevier-Gobeaux et al, 2005**[10]	Independent adjudication by two ED clinicians on the basis of clinical examination, medical history, ECG, chest X-ray and blood analysis (including plasma creatinine).	Patient selection: low; index test: low; reference standard: low; flow and timing: low; Overall: low.
**deFilippi et al, 2007**[11]	Adjudication by cardiologist who reviewed case report forms blinded to natriuretic peptide results. Subset of 50 random cases adjudicated by second cardiologist, demonstrating good agreement.	Patient selection: high; index test: low; reference standard: low; flow and timing: low; Overall: high.
**Gargani et al, 2008**[12]	Independent adjudication by two cardiologists who were blinded to the NT- proBNP values, who reviewed all of the medical records pertaining to the patient.	Patient selection: high; index test: low; reference standard: low; flow and timing: high; Overall: high.
**Ibrahim et al, 2017**[13]	Independent adjudication by an ED specialist and a cardiologist. They were blinded to NT-proBNP measurements but could access medical records, case report forms, and other test results including cardiac imaging as available.	Patient selection: low; index test: low; reference standard: low; flow and timing: low; Overall: low.
**Januzzi et al, 2006**[14]	Adjudicated by a panel of physicians or cardiologists utilizing all available clinical data, blinded to NTproBNP results.	Patient selection: low; index test: low; reference standard: low; flow and timing: low; Overall: low.
**Maisel et al, 2010**[15]	Independent adjudication by two cardiologists who reviewed all medical records.	Patient selection: low; index test: low; reference standard: low; flow and timing: low; Overall: low.
**Moe et al, 2007**[16]	Independent adjudication by two cardiologists. They were provided with hospital records, including the discharge summary, results of laboratory and radiographic testing, echocardiograms if performed, clinical notes from the time of ED presentation to the 60-day follow-up, and outcome of a telephone interview. Using all available data, the cardiologists assigned a diagnosis without knowledge of the NT- proBNP results.	Patient selection: low; index test: low; reference standard: low; flow and timing: low; Overall: low.
**Mueller et al, 2005**[17]	Retrospective review of all medical records by a clinician based on Framingham criterion	Patient selection: low; index test: low; reference standard: low; flow and timing: low; Overall: low.
**Nazerian et al, 2010**[18]	Independent adjudication by two cardiologists and one respiratory physician, blinded to echocardiogram and NTproBNP results. The reviewers had access to ED records, clinical notes, components and summary of the Framingham Heart Study Criteria and any additional information that became available during hospital stay.	Patient selection: high; index test: low; reference standard: low; flow and timing: low; Overall: high.

**Rutten et al, 2008**[19]	Consensus between two clinicians in internal medicine, pulmonology or cardiology.	Patient selection: low; index test: low; reference standard: high; flow and timing: low; Overall: high.
**Wussler et al, 2019**[20]	Adjudicated by 2 independent cardiologist-internists who had access to all patients' medical records, including clinical history, physical examination, 12-lead electrocardiograms, laboratory findings, chest radiographs, echocardiograms, lung function test results, computed tomography scans, and response to therapy, as well as autopsy data for patients who died in the hospital.	Patient selection: high; index test: low; reference standard: high; flow and timing: low; Overall: high.

Abbreviations: ESC= European society of cardiology; ED= emergency department; ECG= electrocardiogram.

Supplementary Table B. Baseline characteristics of patients within each study.

	Overall	Bahrmann et al	Behnes et al	Bombelli et al	Chenevier-Gobeaux et al	De Filippi et al
**Number of participants**	10369	303	401	895	380	831
**Men**	5531 (53.3)	148 (48.8)	205 (51.1)	368 (41.1)	189 (49.7)	380 (45.7)
**Age, years**	69.3 (16.3)	80.4 (5.9)	67.4 (15.6)	85.7 (4.2)	78.5 (12.2)	66.3 (14.9)
< 50	1377 (13.3)	-	53 (13.2)	-	5 (1.3)	125 (15.0)
50-75	4370 (42.1)	79 (26.1)	211 (52.6)	-	121 (31.8)	443 (53.3)
>75	4622 (44.6)	224 (73.9)	137 (34.2)	895 (100.0)	254 (66.8)	263 (31.6)
**Ethnicity**						
Black	845 (14.8)	0 (0.0)	NR	NR	NR	318 (38.3)
Caucasian	4112 (72.1)	303 (100.0)	NR	NR	NR	499 (60.0)
Other	743 (13.0)	0 (0.0)	NR	NR	NR	14 (1.7)
**Past medical history**						
Prior Heart failure	3119 (33.4)	224 (73.9)	194 (48.4)	NR	128 (33.8)	287 (36.1)
Ischemic heart disease	2953 (32.3)	138 (45.5)	157 (39.2)	NR	124 (32.6)	263 (33.1)
Diabetes Mellitus	2398 (26.7)	117 (38.6)	120 (30.1)	NR	NR	305 (38.2)
Hypertension	5071 (59.3)	255 (84.4)	268 (67.0)	NR	153 (40.3)	NR
Hyperlipidemia	2269 (41.2)	206 (68.0)	122 (30.7)	NR	NR	NR
Current or ex-smoker	2458 (41.3)	149 (49.2)	206 (53.9)	NR	NR	NR
Asthma	770 (18.5)	NR	21 (5.2)	NR	NR	NR
COPD	2117 (29.2)	87 (28.7)	94 (23.4)	NR	127 (33.4)	NR
Atrial fibrillation	1701 (20.9)	89 (29.4)	70 (17.5)	NR	NR	175 (22.0)
Chronic Kidney Disease	1215 (18.9)	18 (5.9)	71 (17.7)	NR	NR	NR
**Body mass index, kg/m^2**	27.7 (7.2)	27.1 (5.0)	27.9 (6.2)	NR	NR	30.3 (9.7)
<25	3062 (39.0)	104 (34.3)	118 (32.6)	NR	NR	195 (31.5)
25-30	2473 (31.5)	121 (39.9)	146 (40.3)	NR	NR	172 (27.8)
≥30	2317 (29.5)	78 (25.7)	98 (27.1)	NR	NR	252 (40.7)
**Physiological parameters**						
Heart rate, beats per minute	91.7 (23.7)	85.2 (23.2)	91.7 (24.0)	NR	NR	NR
Systolic blood pressure, mmHg	140.0 (27.9)	146.5 (27.3)	139.2 (28.6)	NR	NR	NR
Diastolic blood pressure, mmHg	79.7 (17.0)	74.9 (15.7)	79.0 (13.7)	NR	NR	NR
**Clinical hematology and biochemistry**						
Hemoglobin, g/dL	13.1 (2.1)	12.4 (2.3)	13.2 (1.9)	NR	NR	NR
eGFR, mL/min/1.73m^2	68.2 (31.3)	48.0 (14.8)	66.8 (24.9)	52.9 (25.4)	54.7 (19.9)	63.3 (34.0)
NT proBNP, pg/mL	1182.2 [191.0, 4737.0]	1594.0 [433.5, 4976.5]	766.0 [150.6, 3155.1]	2937.0 [1014.0, 7598.5]	1678.0 [409.8, 5875.0]	1731.0 [472.0, 6032.5]
**Adjudicated diagnosis of heart failure**	4549 (43.9)	168 (55.4)	122 (30.4)	405 (45.3)	115 (30.3)	437 (52.6)

Presented as No. (%), mean (SD) or median [inter-quartile range]. Abbreviations: COPD= chronic obstructive pulmonary disease; eGFR= estimated glomerular filtration rate; NT-proBNP= N-terminal pro-B-type natriuretic peptide; CVD= cardiovascular disease; NR= not reported.

	Gargani et al	Ibrahim et al	Januzzi et al	Maisel et al	Moe et al	Mueller et al
**Number of participants**	149	1106	1256	1623	500	251
**Men**	98 (65.8)	683 (61.8)	643 (51.2)	848 (52.2)	258 (51.6)	234 (93.2)
**Age, years**	70.8 (11.0)	62.1 (16.2)	68.4 (15.9)	63.8 (16.9)	70.7 (14.3)	70.2 (14.0)
< 50	5 (3.4)	232 (21.0)	183 (14.6)	332 (20.5)	46 (9.2)	24 (9.6)
50-75	91 (61.1)	601 (54.3)	554 (44.1)	800 (49.3)	231 (46.2)	115 (45.8)
>75	53 (35.6)	273 (24.7)	519 (41.3)	491 (30.3)	223 (44.6)	112 (44.6)
**Ethnicity**						
Black	0 (0.0)	0 (0.0)	46 (3.7)	471 (29.3)	10 (2.0)	0 (0.0)
Caucasian	149 (100.0)	461 (41.7)	1210 (96.3)	1078 (67.0)	464 (93.0)	251 (100.0)
Other	0 (0.0)	645 (58.3)	0 (0.0)	59 (3.7)	25 (5.0)	0 (0.0)
**Past medical history**						
Prior Heart failure	46 (30.9)	235 (21.3)	429 (34.2)	563 (35.6)	171 (38.2)	75 (29.9)
Ischemic heart disease	60 (40.3)	110 (11.8)	526 (42.0)	498 (31.7)	135 (31.1)	117 (46.6)
Diabetes Mellitus	57 (38.3)	293 (26.5)	309 (24.6)	457 (28.5)	113 (26.2)	58 (23.1)
Hypertension	79 (53.0)	601 (54.3)	661 (52.6)	1069 (66.9)	262 (58.9)	141 (56.2)
Hyperlipidemia	57 (38.3)	472 (43.1)	NR	NR	NR	NR
Current or ex-smoker	13 (9.6)	245 (22.2)	721 (57.5)	463 (29.4)	311 (65.8)	46 (100.0)
Asthma	2 (1.3)	282 (25.6)	NR	313 (19.9)	88 (19.6)	NR
COPD	33 (22.1)	254 (23.0)	NR	465 (29.5)	126 (30.3)	72 (28.7)
Atrial fibrillation	30 (27.0)	164 (16.1)	280 (22.3)	240 (16.8)	NR	83 (33.1)
Chronic Kidney Disease	43 (29.1)	121 (11.0)	NR	244 (15.6)	NR	74 (29.5)
**Body mass index, kg/m^2**	27.7 (5.1)	27.4 (7.5)	27.4 (6.3)	29.2 (8.8)	28.5 (7.4)	26.8 (5.0)
<25	42 (30.7)	308 (43.0)	412 (37.4)	490 (35.4)	157 (33.0)	93 (37.1)
25-30	54 (39.4)	195 (27.2)	375 (34.0)	384 (27.7)	162 (34.0)	100 (39.8)
>=30	41 (29.9)	213 (29.7)	316 (28.6)	511 (36.9)	157 (33.0)	58 (23.1)
**Physiological parameters**						
Heart rate, beats per minute	78.1 (16.6)	93.2 (23.0)	91.5 (24.9)	91.4 (22.8)	86.5 (21.7)	93.8 (26.1)
Systolic blood pressure, mmHg	131.7 (23.4)	139.9 (26.9)	NR	140.9 (28.7)	136.1 (25.4)	140.1 (31.9)
Diastolic blood pressure, mmHg	73.1 (10.8)	79.9 (16.1)	NR	80.8 (17.4)	76.8 (17.0)	81.8 (16.3)
**Clinical hematology and biochemistry**						
Hemoglobin, g/dL	13.4 (0.4)	13.4 (2.0)	13.0 (2.0)	12.9 (2.2)	13.0 (1.8)	13.7 (2.0)
eGFR, mL/min/1.73m^2	59.9 (23.1)	72.9 (27.7)	72.5 (31.9)	70.4 (30.4)	76.4 (30.1)	94.0 (44.6)
NT proBNP, pg/mL	2303.0 [430.0, 5034.0]	531.8 [87.7, 3149.1]	1403.5 [150.1, 6284.5]	832.9 [111.5, 4114.5]	1293.5 [212.8, 4199.5]	1222.0 [269.4, 3981.5]
**Adjudicated diagnosis of heart failure**	122 (81.9)	327 (29.6)	720 (57.3)	562 (34.6)	230 (46.0)	137 (54.6)

	Nazerian et al	Rutten et al	Wussler et al
**Number of participants**	145	476	2053
**Men**	74 (51.0)	257 (54.0)	1146 (55.8)
**Age, years**	77.8 (11.2)	58.6 (17.8)	70.5 (15.5)
< 50	4 (2.8)	144 (30.3)	224 (10.9)
50-75	43 (29.7)	239 (50.2)	842 (41.0)
>75	98 (67.6)	93 (19.5)	987 (48.1)
**Ethnicity**			
Black	NR	NR	NR
Caucasian	NR	NR	NR
Other	NR	NR	NR
**Past medical history**			
Prior Heart failure	30 (20.7)	85 (17.9)	652 (32.0)
Ischemic heart disease	47 (32.4)	100 (21.0)	678 (33.2)
Diabetes Mellitus	25 (17.2)	77 (16.2)	467 (22.8)
Hypertension	84 (57.9)	120 (25.2)	1378 (67.5)
Hyperlipidemia	NR	NR	846 (41.9)
Current or ex-smoker	NR	304 (64.5)	NR
Asthma	NR	64 (13.4)	NR
COPD	49 (33.8)	126 (26.5)	684 (33.4)
Atrial fibrillation	50 (34.5)	70 (19.2)	450 (21.9)
Chronic Kidney Disease	15 (10.3)	41 (8.6)	588 (28.7)
**Body mass index, kg/m^2**	NR	25.6 (5.7)	26.6 (6.1)
<25	NR	247 (52.2)	896 (44.2)
25-30	NR	138 (29.2)	626 (30.9)
>=30	NR	88 (18.6)	505 (24.9)
**Physiological parameters**			
Heart rate, beats per minute	98.3 (21.2)	97.4 (23.9)	92.6 (23.9)
Systolic blood pressure, mmHg	137.6 (28.2)	147.9 (33.2)	138.5 (26.1)
Diastolic blood pressure, mmHg	76.0 (15.8)	81.5 (19.5)	80.2 (17.4)
**Clinical hematology and biochemistry**			
Hemoglobin, g/dL	13.5 (2.3)	13.6 (2.3)	13.1 (2.1)
eGFR, mL/min/1.73m^2	52.6 (22.1)	88.7 (40.2)	67.4 (29.2)
NT proBNP, pg/mL	2355.0 [764.0, 7806.0]	48.8 [8.3, 314.2]	1333.0 [230.0, 5178.0]
**Adjudicated diagnosis of heart failure**	64 (44.1)	97 (20.4)	1043 (50.8)

Supplementary Table C. Baseline characteristics of study patients stratified by prior history of heart failure.

	Overall	No prior history of heart failure	Prior history of heart failure
**Number of participants**	10369	6208	3119
**Men**	5531 (53.3)	3271 (52.7)	1818 (58.3)
**Age, years**	69.3 (16.3)	64.7 (16.9)	73.7 (12.7)
< 50	1377 (13.3)	1191 (19.2)	168 (5.4)
50-75	4370 (42.1)	3012 (48.5)	1298 (41.6)
>75	4622 (44.6)	2005 (32.3)	1653 (53.0)
**Ethnicity**			
Black	845 (14.8)	522 (13.9)	303 (16.8)
Caucasian	4112 (72.1)	2634 (70.0)	1373 (76.2)
Other	743 (13.0)	609 (16.2)	127 (7.0)
**Past medical history**			
Ischemic heart disease	2953 (32.3)	1228 (20.4)	1687 (55.6)
Diabetes Mellitus	2398 (26.7)	1270 (21.5)	1096 (37.1)
Hypertension	5071 (59.3)	2950 (52.2)	2057 (73.4)
Hyperlipidemia	2269 (41.2)	1156 (32.1)	1092 (58.5)
Current or ex-smoker	2458 (41.3)	1645 (42.3)	767 (38.9)
Asthma	770 (18.5)	621 (22.1)	131 (10.3)
COPD	2117 (29.2)	1361 (28.3)	718 (30.4)
Atrial fibrillation	1701 (20.9)	822 (15.5)	861 (31.1)
Chronic Kidney Disease	1215 (18.9)	493 (11.4)	712 (34.2)
**Body mass index, kg/m^2**	27.7 (7.2)	27.5 (7.2)	28.0 (7.2)
<25	3062 (39.0)	2053 (40.6)	963 (36.1)
25-30	2473 (31.5)	1532 (30.3)	900 (33.8)
>=30	2317 (29.5)	1477 (29.2)	801 (30.1)
**Physiological parameters**			
Heart rate, beats per minute	91.7 (23.7)	93.2 (23.5)	88.8 (23.9)
Systolic blood pressure, mmHg	140.0 (27.9)	141.9 (27.1)	136.2 (29.2)
Diastolic blood pressure, mmHg	79.7 (17.0)	81.0 (16.7)	77.1 (17.2)
**Clinical hematology and biochemistry**			
Hemoglobin, g/dL	13.1 (2.1)	13.4 (2.1)	12.6 (2.1)
eGFR, mL/min/1.73m^2	68.2 (31.3)	77.0 (31.5)	55.1 (25.8)
NT proBNP, pg/mL	1182.2 [191.0, 4737.0]	421.5 [83.8, 2270.2]	3484.0 [1162.5, 8905.5]
**Adjudicated diagnosis of heart failure**	4549 (43.9)	1802 (29.0)	2286 (73.3)

Presented as No. (%), mean (SD) or median [inter-quartile range]. Abbreviations: COPD= chronic obstructive pulmonary disease; eGFR= estimated glomerular filtration rate; NT-proBNP= N-terminal pro-B-type natriuretic peptide; CVD= cardiovascular disease.

Supplementary Table D. Diagnostic performance of NT-proBNP for acute heart failure.

NT-proBNP threshold (pg/mL)	True positive	False positive	True negative	False negative	Negative predictive value (NPV)	Positive predictive value (PPV)	Sensitivity	Specificity
**50**	4529	4676	1144	20	98.3 (97.2-99.0)	49.8 (41.9-57.7)	99.7 (99.2-99.9)	9.1 (3.6-21.3)
**100**	4507	4011	1809	42	97.8 (95.8-98.8)	53.8 (44.8-62.6)	99.3 (98.5-99.7)	22.8 (13.2-36.4)
**150**	4481	3557	2263	68	97.3 (95.1-98.6)	56.6 (47.3-65.5)	98.9 (97.8-99.5)	32.3 (21.3-45.7)
**200**	4451	3280	2540	98	96.6 (94.2-98.0)	58.5 (49.0-67.3)	98.4 (96.9-99.2)	38.1 (26.5-51.3)
**250**	4417	3045	2775	132	95.2 (92.5-97.0)	60.6 (50.3-70.1)	97.4 (95.5-98.5)	43.9 (31.1-57.6)
**300**	4388	2833	2987	161	94.6 (91.9-96.4)	62.9 (51.3-73.3)	96.8 (94.6-98.1)	49.3 (35.4-63.4)
**350**	4359	2648	3172	190	93.8 (90.7-96.0)	64.6 (52.6-75.1)	96.0 (93.4-97.6)	53.8 (39.8-67.3)
**400**	4331	2500	3320	218	93.3 (90.1-95.6)	65.7 (53.7-76.0)	95.3 (92.4-97.2)	56.5 (42.6-69.4)
**450**	4292	2364	3456	257	92.4 (88.6-95.0)	66.7 (54.9-76.8)	94.3 (90.7-96.6)	59.6 (46.2-71.7)
**500**	4262	2247	3573	287	91.7 (87.7-94.5)	67.8 (56.0-77.6)	93.5 (89.8-96.0)	61.8 (48.4-73.6)
**550**	4243	2143	3677	306	91.6 (87.6-94.4)	69.0 (57.3-78.7)	93.1 (89.1-95.7)	64.3 (51.1-75.6)
**600**	4219	2066	3754	330	91.1 (86.9-94.1)	69.5 (58.1-79.0)	92.5 (88.2-95.3)	65.7 (53.1-76.5)
**650**	4177	1967	3853	372	90.5 (86.1-93.7)	70.5 (59.3-79.6)	91.6 (87.1-94.7)	67.7 (55.4-78.0)
**700**	4155	1897	3923	394	90.2 (85.7-93.4)	71.0 (60.0-80.0)	91.1 (86.5-94.3)	68.9 (57.1-78.7)
**750**	4126	1829	3991	423	89.7 (85.0-93.1)	71.9 (61.0-80.7)	90.4 (85.7-93.7)	70.5 (58.7-80.1)
**800**	4096	1769	4051	453	89.3 (84.3-92.9)	72.6 (61.8-81.2)	89.8 (84.7-93.3)	71.8 (60.3-81.0)
**850**	4061	1695	4125	488	88.7 (83.5-92.3)	73.2 (62.4-81.7)	88.9 (83.7-92.6)	72.9 (61.7-81.8)
**900**	4025	1613	4207	524	88.2 (83.0-91.9)	74.0 (63.3-82.4)	88.1 (82.7-92.0)	74.3 (63.4-82.9)
**950**	3993	1553	4267	556	87.5 (82.2-91.4)	74.5 (63.8-82.9)	87.1 (81.4-91.3)	75.4 (64.5-83.7)
**1000**	3959	1511	4309	590	87.0 (81.6-91.0)	74.9 (64.4-83.2)	86.3 (80.6-90.6)	76.1 (65.6-84.2)
**1050**	3921	1459	4361	628	86.3 (80.8-90.5)	75.3 (64.9-83.4)	85.4 (79.5-89.8)	76.9 (66.8-84.6)
**1100**	3883	1412	4408	666	85.9 (80.3-90.1)	75.7 (65.4-83.6)	84.7 (78.7-89.2)	77.6 (67.7-85.2)
**1150**	3855	1382	4438	694	85.5 (79.7-89.8)	75.9 (65.7-83.8)	83.9 (77.7-88.7)	78.1 (68.5-85.5)
**1200**	3814	1336	4484	735	84.9 (79.1-89.3)	76.4 (66.1-84.3)	83.0 (76.5-88.0)	79.1 (69.5-86.2)
**1250**	3780	1300	4520	769	84.4 (78.5-88.9)	76.7 (66.6-84.5)	82.2 (75.6-87.2)	79.7 (70.4-86.6)
**1300**	3750	1273	4547	799	84.1 (78.1-88.6)	77.1 (67.0-84.9)	81.5 (74.8-86.7)	80.3 (71.2-87.1)
**1350**	3720	1231	4589	829	83.9 (77.9-88.5)	77.9 (67.9-85.4)	81.0 (74.2-86.3)	81.3 (72.4-87.9)
**1400**	3685	1211	4609	864	83.4 (77.3-88.1)	78.0 (68.0-85.5)	80.1 (73.1-85.7)	81.7 (72.9-88.1)
**1450**	3662	1178	4642	887	83.2 (77.0-88.0)	78.4 (68.5-85.8)	79.6 (72.5-85.3)	82.3 (73.7-88.5)
**1500**	3625	1148	4672	924	82.6 (76.3-87.6)	78.7 (68.9-86.0)	78.7 (71.6-84.4)	82.8 (74.4-88.8)
**1550**	3608	1125	4695	941	82.5 (76.1-87.4)	79.0 (69.3-86.2)	78.4 (71.1-84.2)	83.2 (74.9-89.1)
**1600**	3574	1104	4716	975	82.0 (75.7-86.9)	79.2 (69.5-86.4)	77.5 (70.4-83.3)	83.5 (75.4-89.3)
**1650**	3547	1085	4735	1002	81.6 (75.2-86.6)	79.2 (69.6-86.4)	76.8 (69.5-82.8)	83.8 (75.9-89.4)
**1700**	3516	1068	4752	1033	81.3 (74.8-86.4)	79.4 (69.8-86.5)	76.2 (69.0-82.2)	84.1 (76.3-89.6)
**1750**	3484	1039	4781	1065	80.8 (74.4-85.9)	79.7 (70.2-86.7)	75.3 (68.2-81.3)	84.5 (77.0-89.9)
**1800**	3458	1020	4800	1091	80.5 (74.1-85.6)	79.8 (70.4-86.8)	74.7 (67.6-80.7)	84.8 (77.5-90.0)
**1850**	3430	995	4825	1119	80.2 (73.8-85.3)	80.4 (70.9-87.4)	74.1 (66.9-80.2)	85.5 (78.1-90.6)
**1900**	3400	980	4840	1149	79.7 (73.3-84.8)	80.5 (70.9-87.6)	73.2 (66.0-79.3)	85.8 (78.5-90.9)
**1950**	3360	962	4858	1189	79.2 (72.9-84.4)	80.9 (71.3-87.8)	72.3 (65.2-78.4)	86.2 (79.1-91.2)

**2000**	3329	942	4878	1220	78.8 (72.4-84.1)	81.3 (71.7-88.1)	71.4 (64.4-77.6)	86.7 (79.8-91.5)

Supplementary Table E. Sensitivity analysis of the diagnostic performance of guideline-recommended and age-specific thresholds of NT-proBNP for acute heart failure in studies where the reference standard was blinded to NT-proBNP concentration.

Age groups	NT-proBNP threshold (pg/mL)	True positive	False positive	True negative	False negative	Negative predictive value (NPV)	Positive predictive value (PPV)	Sensitivity	Specificity
**<50 years**	450	170	107	719	12	98.7 (95.2-99.7)	61.7 (54.1-68.7)	93.4 (88.8-96.2)	85.4 (74.2-92.3)
**50-75 years**	900	1095	487	1514	193	88.4 (81.7-92.8)	73.6 (59.7-84.0)	85.2 (81.8-88.0)	77.7 (69.3-84.3)
**>75 years**	1800	1571	557	1046	368	72.7 (62.0-81.3)	79.2 (68.3-87.1)	80.9 (77.2-84.1)	69.8 (62.1-76.5)
**All**	300	3298	2336	2095	111	94.6 (91.4-96.7)	63.2 (49.4-75.1)	97.1 (95.8-98.0)	44.8 (31.1-59.4)

Supplementary Table F. Sensitivity analysis of the diagnostic performance of guideline-recommended and age-specific thresholds of NT-proBNP for acute heart failure in studies with low risk of bias in patient selection.

Age groups	NT-proBNP threshold (pg/mL)	True positive	False positive	True negative	False negative	Negative predictive value (NPV)	Positive predictive value (PPV)	Sensitivity	Specificity
**<50 years**	450	127	75	807	10	98.8 (97.7-99.3)	63.1 (54.3-71.1)	94.2 (83.3-98.2)	91.6 (89.1-93.6)
**50-75 years**	900	834	379	1503	156	90.5 (86.8-93.3)	68.8 (57.6-78.2)	82.6 (71.1-90.2)	81.7 (73.3-87.9)
**>75 years**	1800	942	278	641	241	75.5 (66.4-82.8)	78.0 (64.7-87.3)	78.2 (69.9-84.7)	75.0 (64.6-83.2)
**All**	300	2211	1525	2158	99	95.6 (93.0-97.2)	57.6 (47.1-67.5)	95.7 (91.5-97.9)	57.0 (44.1-69.1)

Supplementary Table G. Diagnostic performance of rule-in and rule-out thresholds and CoDE-HF scores for acute heart failure.

A. Rule-out thresholds and CoDE-HF scores

	Threshold or score	True positive	False positive	True negative	False negative	NPV (95% CI)	Sensitivity (95% CI)	Proportion ruled out	AUC	Brier score
**All patients**										
NT-proBNP	100 pg/mL	4507	4011	1809	42	97.8 (95.8-98.8)	99.3 (98.5-99.7)	17.9%	0.872 (0.865-0.879)	
**Patients without prior heart failure**										
CoDE-HF	4.7	1768	1938	2468	34	98.6 (97.8-99.1)	98.1 (96.9-98.9)	40.3%	0.925 (0.919-0.932)	0.099

B. Rule-in thresholds and CoDE-HF scores

	Threshold or score	True positive	False positive	True negative	False negative	PPV (95% CI)	Specificity (95% CI)	Proportion ruled in	AUC	Brier score
**All patients**										
NT-proBNP	1000 pg/mL	3959	1511	4309	590	74.9 (64.4-83.2)	76.1 (65.6-84.2)	52.8%	0.872 (0.865-0.879)	
**Patients without prior heart failure**										
CoDE-HF	51.2	1326	411	3995	476	75.0 (65.7-82.5)	92.2 (87.5-95.2)	28.0%	0.925 (0.919-0.932)	0.099
**Patients with prior**										

**heart failure**										
CoDE-HF	84.5	1325	95	738	961	92.7 (89.1-95.2)	90.2 (84.0-94.1)	45.5%	0.846 (0.830-0.862)	0.130

Supplementary Table H. Mortality in the pool cohort stratified by CoDE-HF probability group and NT-proBNP threshold of 300 pg/mL

	**CoDE-HF score**			**NT-proBNP concentration**	
	low probability	intermediate-probability	high-probability	<300 pg/mL	≥300 pg/mL
**Cardiovascular mortality**	n = 1239	n = 1721	n = 1593	n = 1448	n = 3105
30 days	2 (0.2%)	14 (0.8%)	66 (4.1%)	2 (0.1%)	80 (2.6%)
1 year	17 (1.4%)	58 (3.4%)	260 (16.3%)	19 (1.3%)	316 (10.2%)
**All-cause mortality**	n = 1251	n =1858	n =1769	n = 1460	n = 3418
30 days	12 (1.0%)	75 (4.0%)	184 (10.4%)	12 (0.8%)	259 (7.6%)
1 year	74 (5.9%)	330 (17.8%)	590 (33.4%)	86 (5.9%)	908 (26.6%)

IV. Supplementary Figures

Supplementary Figure A. Proportion of missing data in the variables included in the diagnostic models across studies.

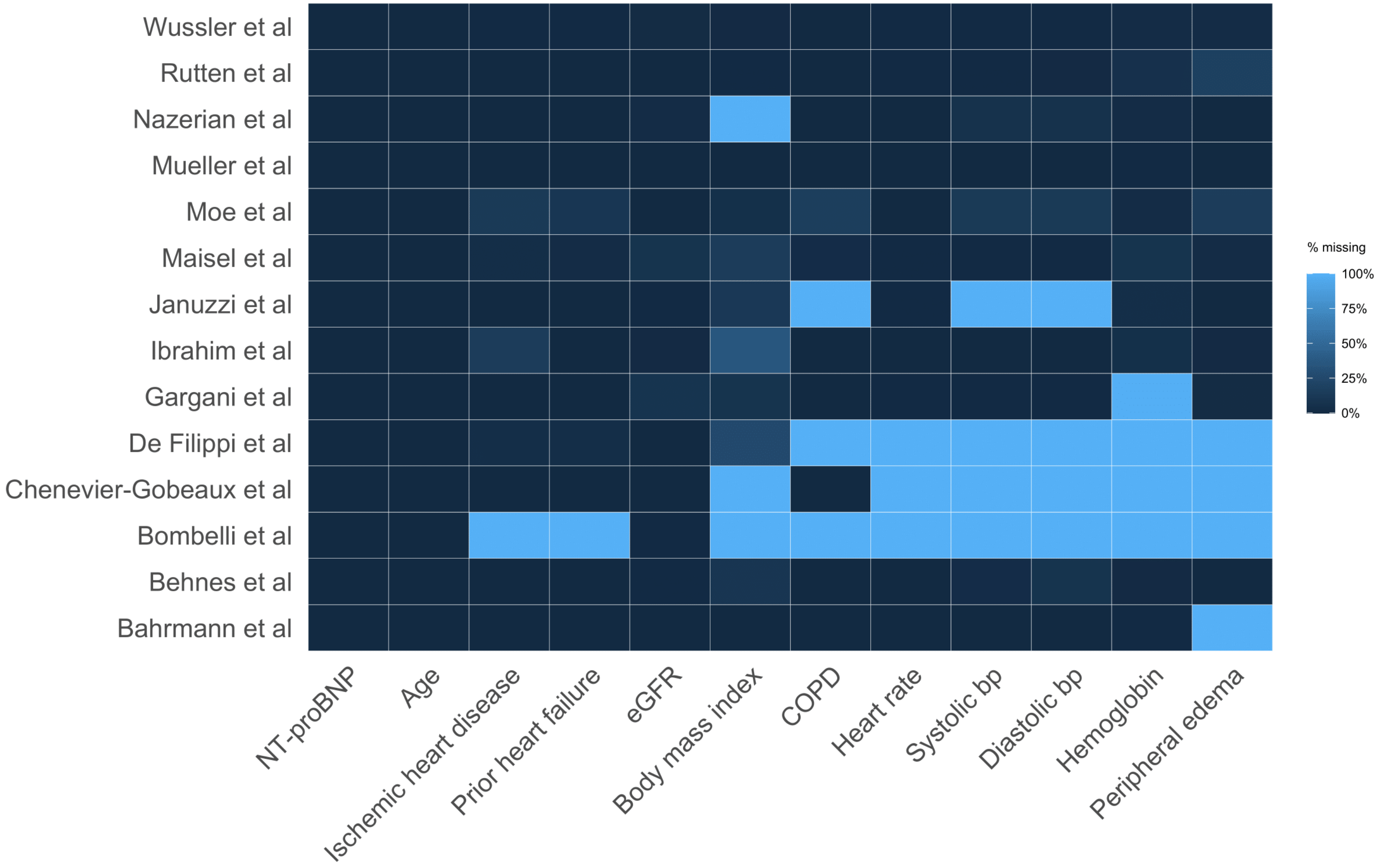

Supplementary Figure B. Flow diagram of study participants.

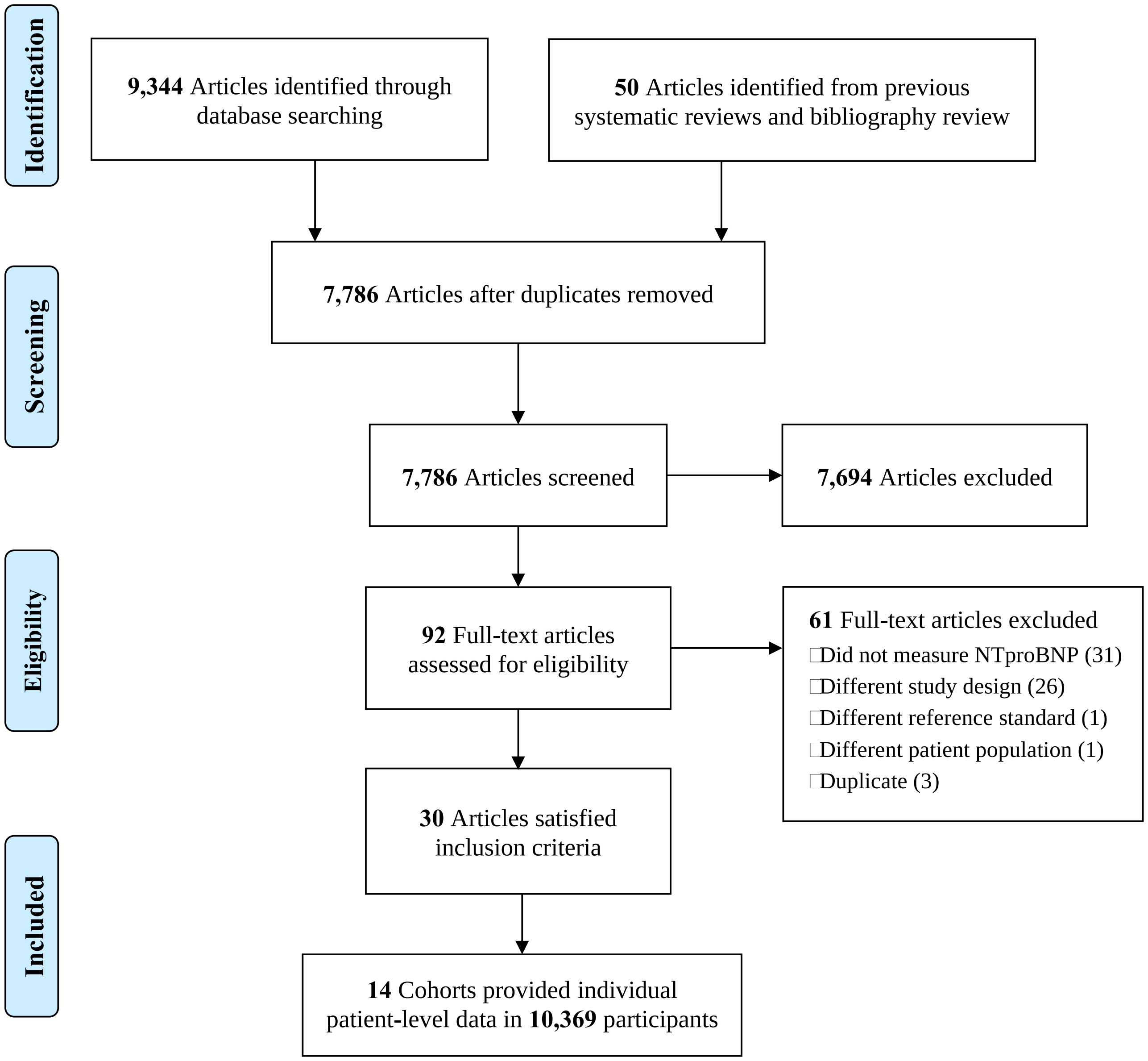

Supplementary Figure C. Negative predictive value of NT-proBNP at the 300 pg/mL threshold across cohorts.

Cohort	Prevalence, %	True negative	False negative	Negative predictive value (95% CI)
Rutten et al	20.4	323	30	91.5 (88.1−94.0)
Ibrahim et al	29.6	468	11	97.7 (95.9−98.7)
Chenevier−Gobeaux et al	30.3	69	1	98.6 (90.6−99.8)
Behnes et al	30.4	134	5	96.4 (91.6−98.5)
Maisel et al	34.6	591	13	97.8 (96.3−98.7)
Nazerian et al	44.1	18	1	94.7 (70.6−99.3)
Bombelli et al	45.3	52	4	92.9 (82.5−97.3)
Moe et al	46	132	14	90.4 (84.5−94.2)
Wussler et al	50.8	569	20	96.6 (94.8−97.8)
De Filippi et al	52.6	120	31	79.5 (72.3−85.2)
Mueller et al	54.6	61	7	89.7 (79.9−95.0)
Bahrmann et al	55.4	45	2	95.7 (84.5−98.9)
Januzzi et al	57.3	380	18	95.5 (92.9−97.1)
Gargani et al	81.9	25	4	86.2 (68.5−94.7)

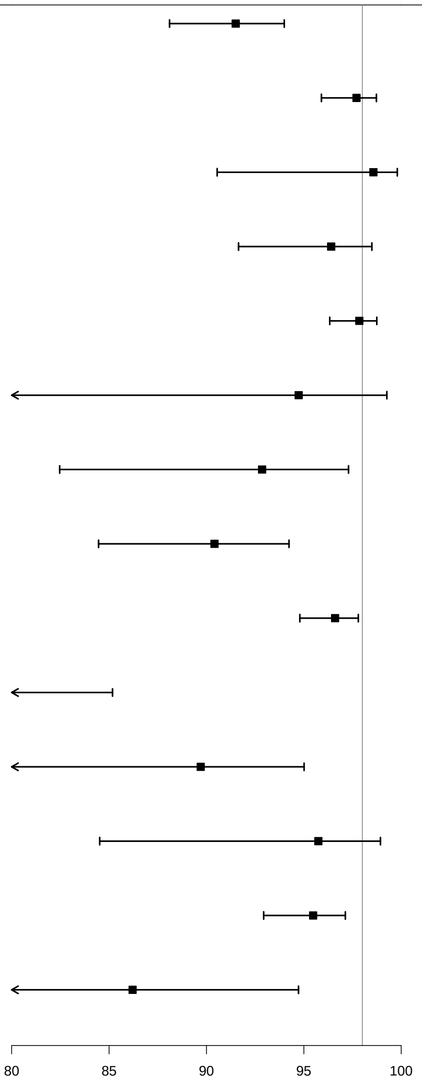

Supplementary Figure D. Meta-regression of the negative predictive value of NT-proBNP at the threshold of 300 pg/mL by prevalence of acute heart failure.

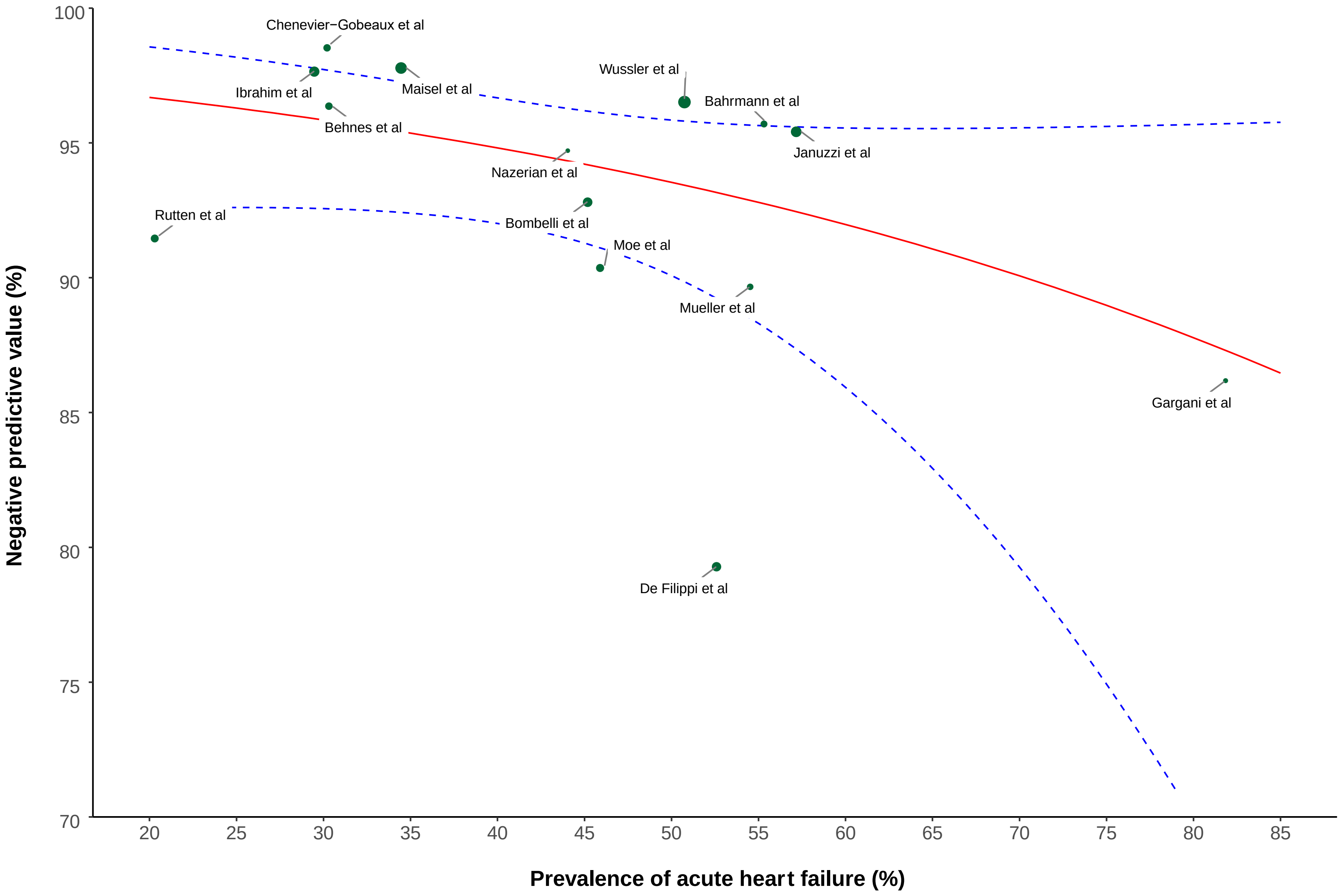

Supplementary Figure E. Positive predictive value of the 300 pg/mL NT-proBNP threshold across patient subgroups.

Subgroups	True positive	False positive	Positive predictive value (95% CI)
Sex			
Male	2481	1441	65.4 (53.3-75.8)
Female	1907	1392	58.2 (48.6-67.2)
Age			
<50 years	211	164	56.3 (51.2-61.2)
50-75 years	1479	1088	59.7 (48.1-70.4)
>75 years	2698	1581	64.5 (53.5-74.2)
Ethnicity			
Black	293	188	61.1 (54.8-67.1)
Caucasian	1961	1043	72.5 (52.3-86.4)
Other	180	168	51.7 (46.5-56.9)
Smoking			
Current or ex-smoker	869	544	60.2 (51.2-68.5)
Non-smoker	1457	845	70.7 (52.2-84.2)
Heart failure			
Yes	2224	630	78.2 (69.2-85.2)
No	1710	1712	45.5 (25.9-66.6)
Ischemic heart disease			
Yes	1819	698	71.2 (60.9-79.7)
No	2027	1600	57.8 (46.5-68.3)
Diabetes			
Yes	1332	545	73.2 (63.4-81.1)
No	2489	1627	61.8 (50.4-72.0)
Hypertension			
Yes	2519	1307	67.4 (55.7-77.2)
No	1021	792	57.1 (42.9-70.2)
Hyperlipidemia			
Yes	1136	567	73.2 (49.8-88.3)
No	1093	857	64.2 (40.8-82.3)
Anaemia			
Yes	1499	730	65.5 (58.4-71.9)
No	1762	1148	58.3 (50.0-66.2)
COPD			
Yes	634	741	48.1 (32.5-64.2)
No	2188	1193	66.5 (54.1-77.0)
Atrial fibrillation			
Yes	1222	376	75.1 (62.5-84.5)
No	2376	1615	62.4 (50.1-73.2)
eGFR			
<60 ml/min	2547	1297	68.5 (58.3-77.3)
60-90 ml/min	1369	1055	56.1 (45.4-66.2)
≥90 ml/min	432	456	46.7 (37.6-55.9)
Body mass index			
<25 (normal/underweight)	1317	771	64.5 (54.7-73.1)
25-29.9 (overweight)	1137	546	68.6 (57.6-77.8)
≥30 (obese)	935	533	65.1 (56.1-73.2)
**Overall**	**4388**	**2833**	**62.9 (51.3-73.3)**

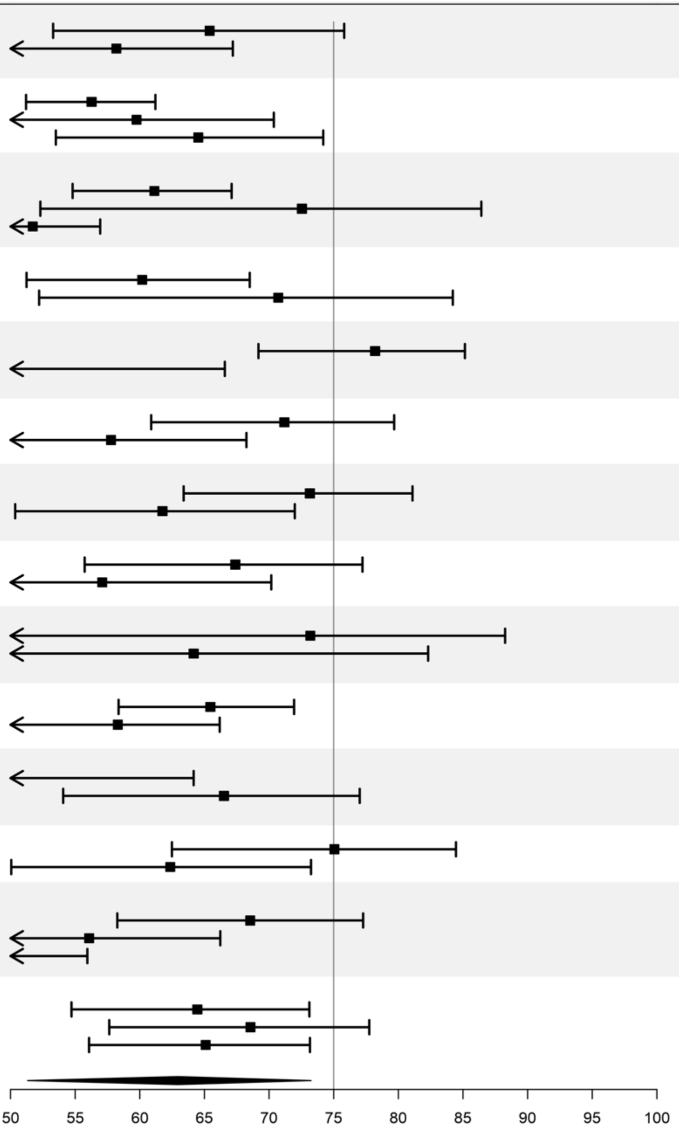

Supplementary Figure F. Positive predictive value of the NT-proBNP threshold of 300 pg/mL across cohorts.

Cohort	Prevalence, %	True positive	False positive	Positive predictive value (95% CI)
Rutten et al	20.4	67	56	54.5 (45.6-63.0)
Ibrahim et al	29.6	316	311	50.4 (46.5-54.3)
Chenevier-Gobeaux et al	30.3	114	196	36.8 (31.6-42.3)
Behnes et al	30.4	117	145	44.7 (38.7-50.7)
Maisel et al	34.6	549	470	53.9 (50.8-56.9)
Nazerian et al	44.1	63	63	50.0 (41.4-58.6)
Bombelli et al	45.3	401	438	47.8 (44.4-51.2)
Moe et al	46	216	138	61.0 (55.8-66.0)
Wussler et al	50.8	1023	441	69.9 (67.5-72.2)
De Filippi et al	52.6	406	274	59.7 (56.0-63.3)
Mueller et al	54.6	130	53	71.0 (64.1-77.1)
Bahrmann et al	55.4	166	90	64.8 (58.8-70.4)
Januzzi et al	57.3	702	156	81.8 (79.1-84.3)
Gargani et al	81.9	118	2	98.3 (93.6-99.6)

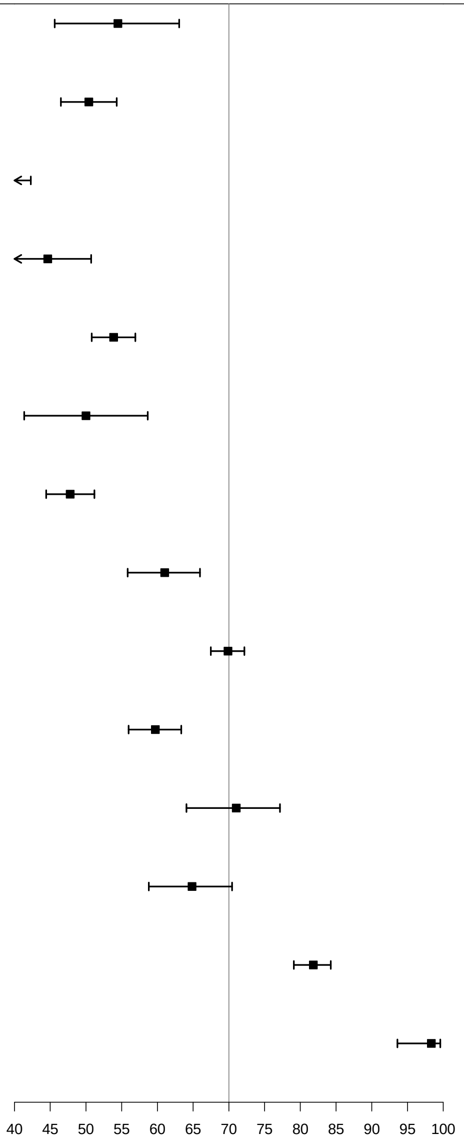

Supplementary Figure G. Meta-regression of positive predictive value of the 300 pg/mL NT-proBNP threshold by prevalence of acute heart failure.

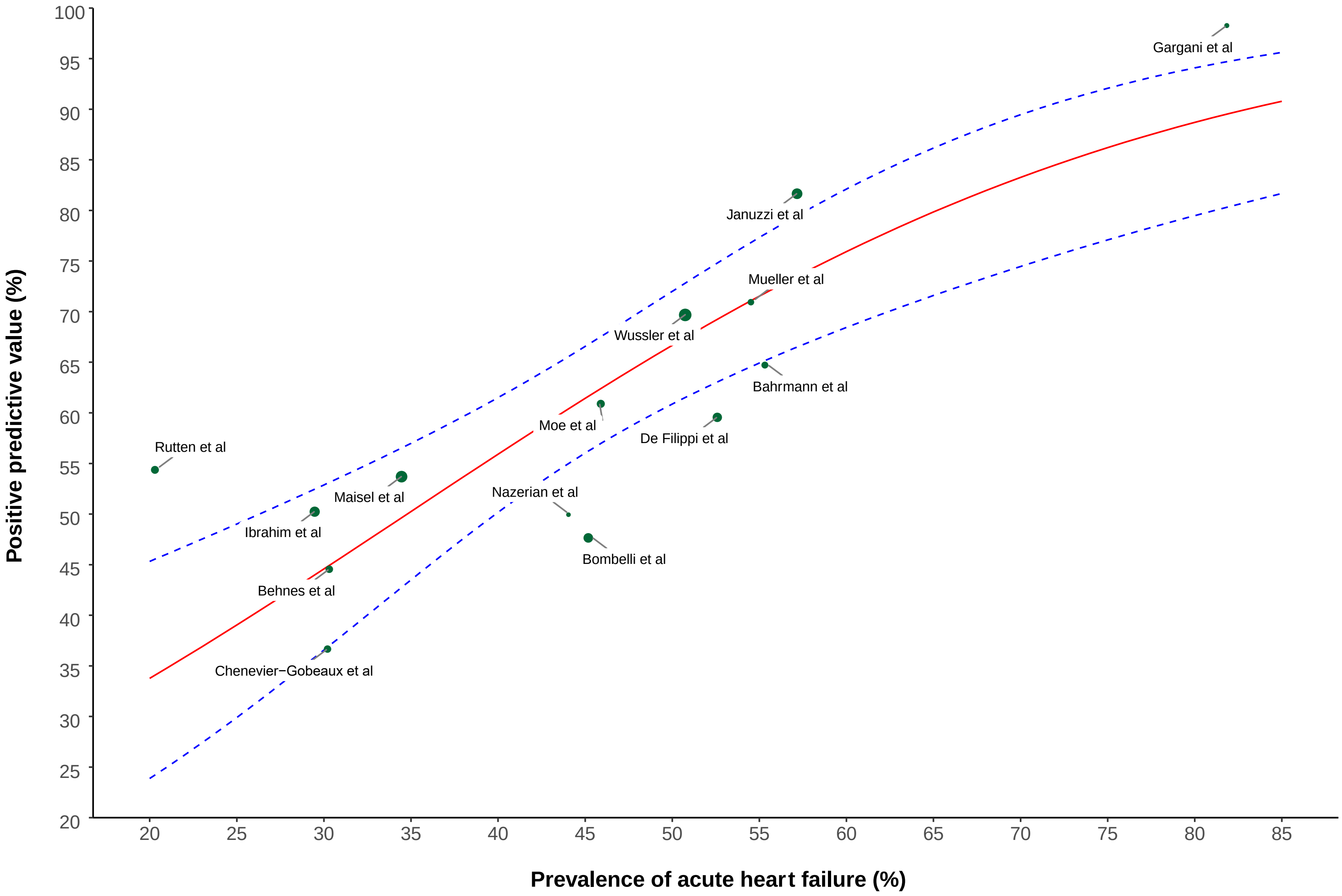

Supplementary Figure |H. Positive predictive value of age-specific thresholds of NT-proBNP across cohorts.

Cohort	Prevalence, %	True positive	False positive	Positive predictive value (95% CI)
Rutten et al	20.4	37	17	68.5 (55.1-79.4)
Ibrahim et al	29.6	291	159	64.7 (60.1-68.9)
Chenevier-Gobeaux et al	30.3	98	100	49.5 (42.6-56.4)
Behnes et al	30.4	102	68	60.0 (52.5-67.1)
Maisel et al	34.6	487	243	66.7 (63.2-70.0)
Nazerian et al	44.1	56	38	59.6 (49.4-69.0)
Bombelli et al	45.3	351	207	62.9 (58.8-66.8)
Moe et al	46	189	71	72.7 (67.0-77.8)
Wussler et al	50.8	872	158	84.7 (82.3-86.7)
De Filippi et al	52.6	333	174	65.7 (61.4-69.7)
Mueller et al	54.6	109	17	86.5 (79.4-91.4)
Bahrmann et al	55.4	140	16	89.7 (83.9-93.6)
Januzzi et al	57.3	590	57	91.2 (88.7-93.1)
Gargani et al	81.9	90	1	98.9 (92.6-99.8)

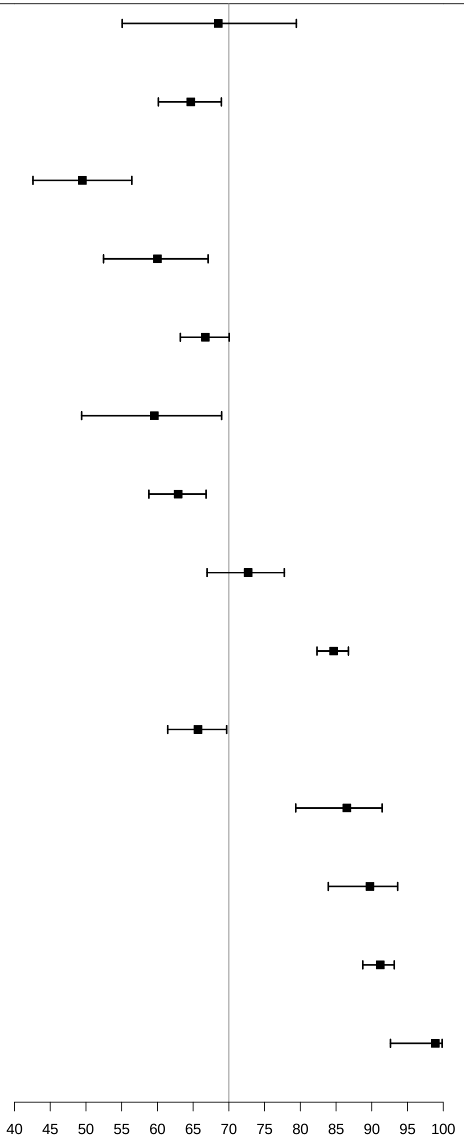

Supplementary Figure I. Meta-regression of positive predictive value of age-specific thresholds of NT-proBNP by prevalence of acute heart failure.

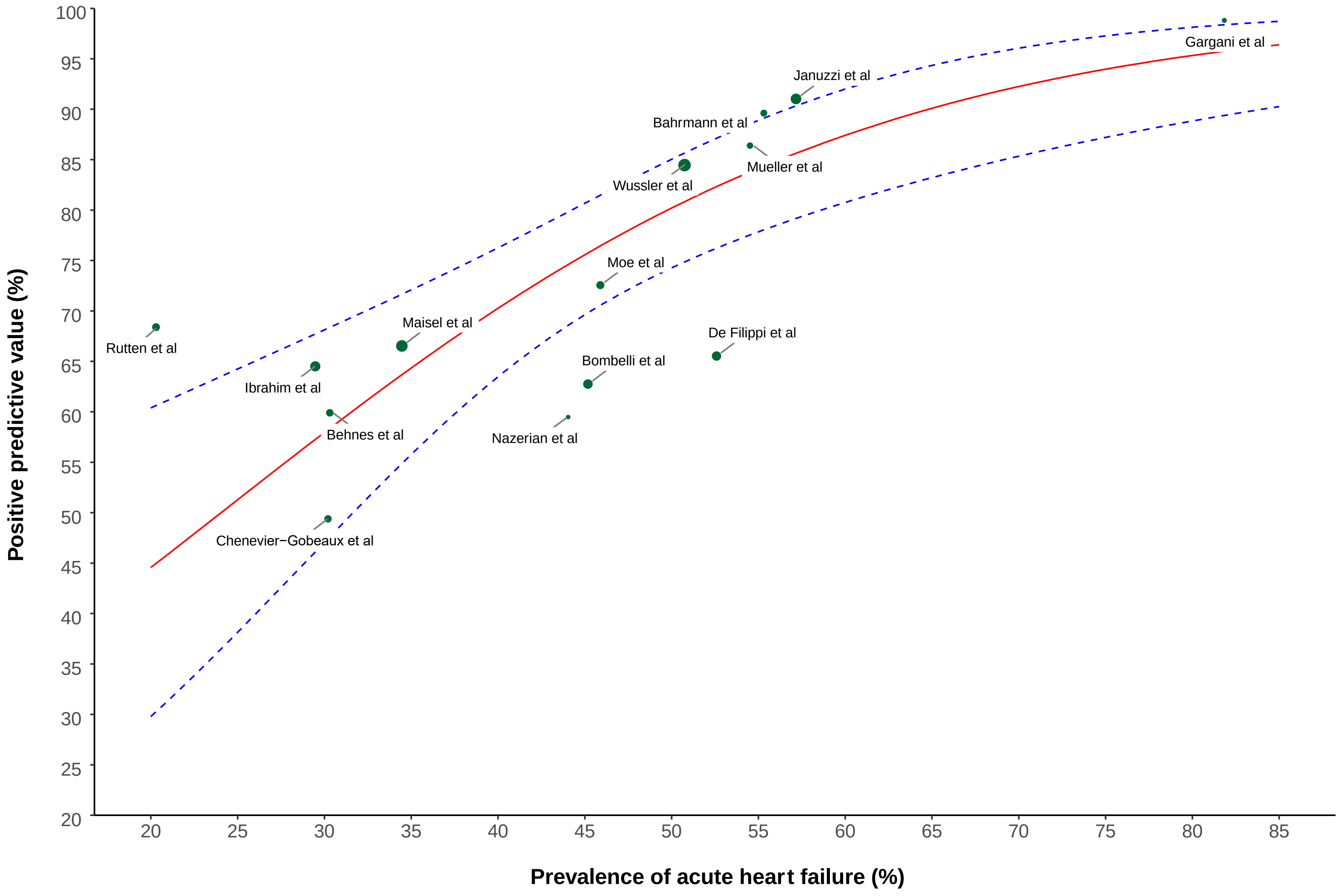

Supplementary Figure J. Negative predictive value of the NT-proBNP threshold of 100 pg/mL across patient subgroups.

Subgroups	True negative	False negative	Negative predictive value (95% CI)
Sex			
Male	957	27	97.2 (94.7-98.5)
Female	852	15	98.5 (96.4-99.4)
Age			
<50 years	805	4	99.6 (97.7-99.9)
50-75 years	877	25	97.9 (94.6-99.2)
>75 years	127	13	90.7 (84.7-94.5)
Ethnicity			
Black	236	9	97.6 (79.2-99.8)
Caucasian	599	15	97.6 (96.0-98.5)
Smoking			
Current or ex-smoker	657	16	98.1 (95.0-99.3)
Non-smoker	737	15	98.0 (95.5-99.1)
Heart failure			
Yes	75	18	81.4 (66.8-90.5)
No	1700	24	98.7 (97.5-99.3)
Ischemic heart disease			
Yes	167	16	92.1 (84.5-96.1)
No	1583	24	98.4 (96.7-99.3)
Diabetes			
Yes	253	12	95.4 (88.8-98.1)
No	1501	28	98.4 (96.6-99.2)
Hypertension			
Yes	592	19	97.0 (93.7-98.6)
No	1137	14	98.9 (97.8-99.4)
Hyperlipidemia			
Yes	270	4	98.5 (96.2-99.5)
No	796	6	99.3 (98.3-99.7)
Anaemia			
Yes	212	8	96.4 (92.9-98.2)
No	1340	24	98.3 (96.9-99.1)
COPD			
Yes	345	9	97.5 (95.0-98.7)
No	1115	18	98.5 (96.7-99.3)
Atrial fibrillation			
Yes	29	6	83.4 (57.3-95.0)
No	1410	31	98.0 (95.5-99.1)
eGFR			
<60 ml/min	119	10	92.9 (84.5-96.9)
60-90 ml/min	571	21	98.2 (93.1-99.6)
≥90 ml/min	1016	11	99.0 (97.9-99.5)
Body mass index			
<25 (normal/underweight)	553	9	99.3 (94.8-99.9)
25-29.9 (overweight)	467	12	97.5 (95.6-98.6)
≥30 (obese)	516	18	96.5 (93.2-98.3)
**Overall**	**1809**	**42**	**97.8 (95.8-98.8)**

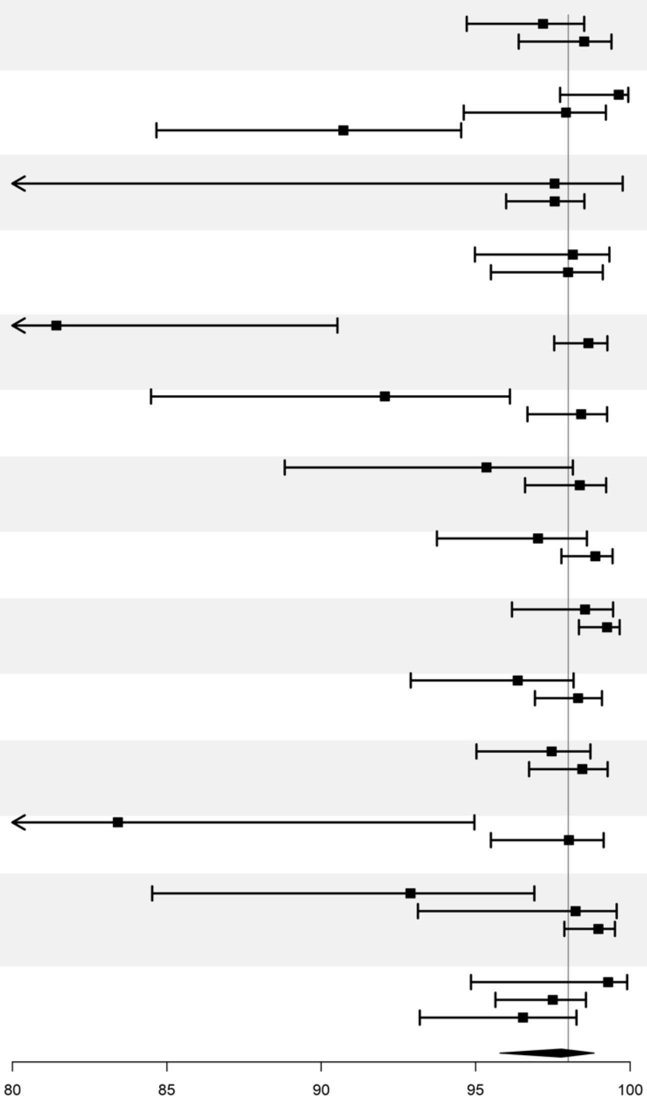

Supplementary Figure K. Positive predictive value of the NT-proBNP threshold of 1000 pg/mL across patient subgroups.

Subgroups	True positive	False positive	Positive predictive value (95% CI)
Sex			
Male	2248	780	77.0 (66.3-85.1)
Female	1711	731	70.8 (60.8-79.1)
Age			
<50 years	178	76	70.1 (64.2-75.4)
50-75 years	1277	512	73.8 (63.2-82.2)
>75 years	2504	923	75.3 (65.0-83.3)
Ethnicity			
Black	252	96	72.4 (67.5-76.9)
Caucasian	1757	568	81.4 (65.0-91.1)
Other	163	86	65.5 (59.3-71.1)
Smoking			
Current or ex-smoker	768	229	78.4 (68.1-86.0)
Non-smoker	1308	454	80.0 (64.2-90.0)
Heart failure			
Yes	2037	383	84.2 (76.8-89.5)
No	1492	808	62.0 (40.8-79.4)
Ischemic heart disease			
Yes	1638	409	79.4 (69.9-86.5)
No	1812	755	72.5 (61.9-81.1)
Diabetes			
Yes	1183	284	83.8 (75.1-89.8)
No	2238	802	74.7 (64.4-82.8)
Hypertension			
Yes	2284	646	79.5 (70.2-86.4)
No	918	391	71.7 (56.9-82.9)
Hyperlipidemia			
Yes	1028	274	82.7 (67.9-91.5)
No	1006	406	76.9 (58.3-88.8)
Anaemia			
Yes	1389	386	77.1 (69.0-83.6)
No	1572	518	74.4 (65.5-81.7)
COPD			
Yes	579	353	65.6 (47.9-79.9)
No	1975	619	77.0 (66.7-84.8)
Atrial fibrillation			
Yes	1139	259	81.1 (69.5-89.0)
No	2082	730	76.4 (66.2-84.3)
eGFR			
<60 ml/min	2395	830	76.6 (67.2-84.0)
60-90 ml/min	1191	502	71.4 (61.0-79.9)
≥90 ml/min	336	166	65.4 (53.7-75.5)
Body mass index			
<25 (normal/underweight)	1220	402	75.0 (66.8-81.8)
25-29.9 (overweight)	1030	268	81.3 (71.6-88.2)
≥30 (obese)	774	221	80.1 (72.9-85.7)
**Overall**	**3959**	**1511**	**74.9 (64.4-83.2)**

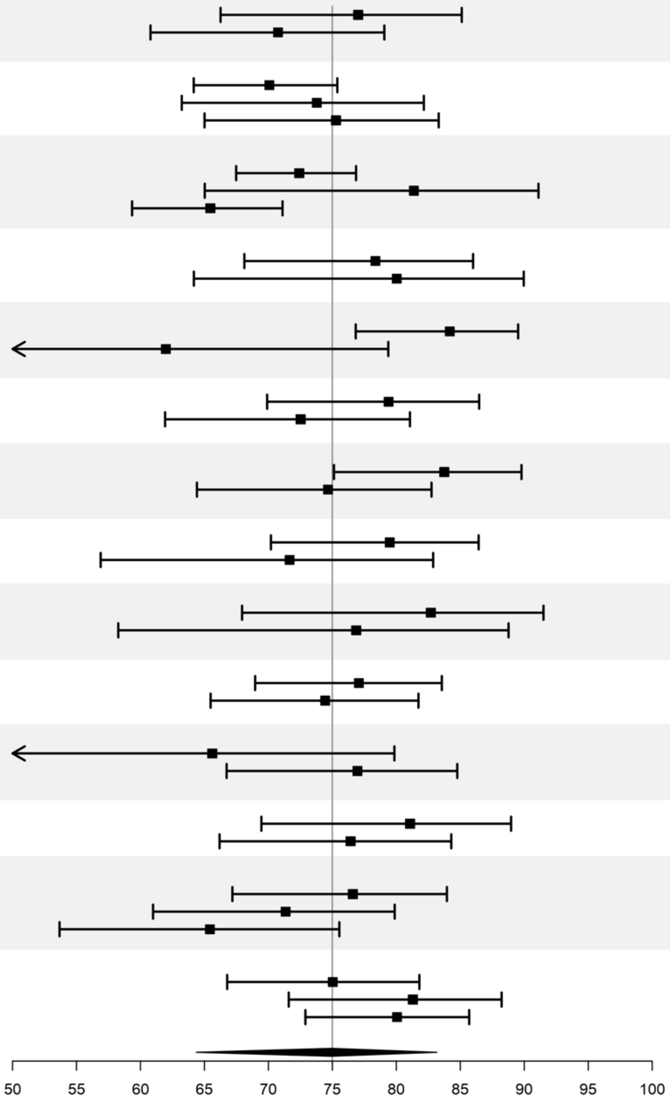

Supplementary Figure L. Discrimination of the guideline-recommended NT-proBNP thresholds and CoDE-HF score

A) **Receiver operator curve in patients without prior heart failure for the CoDE-HF score (area under curve 0.925 [0.919-0.932])**

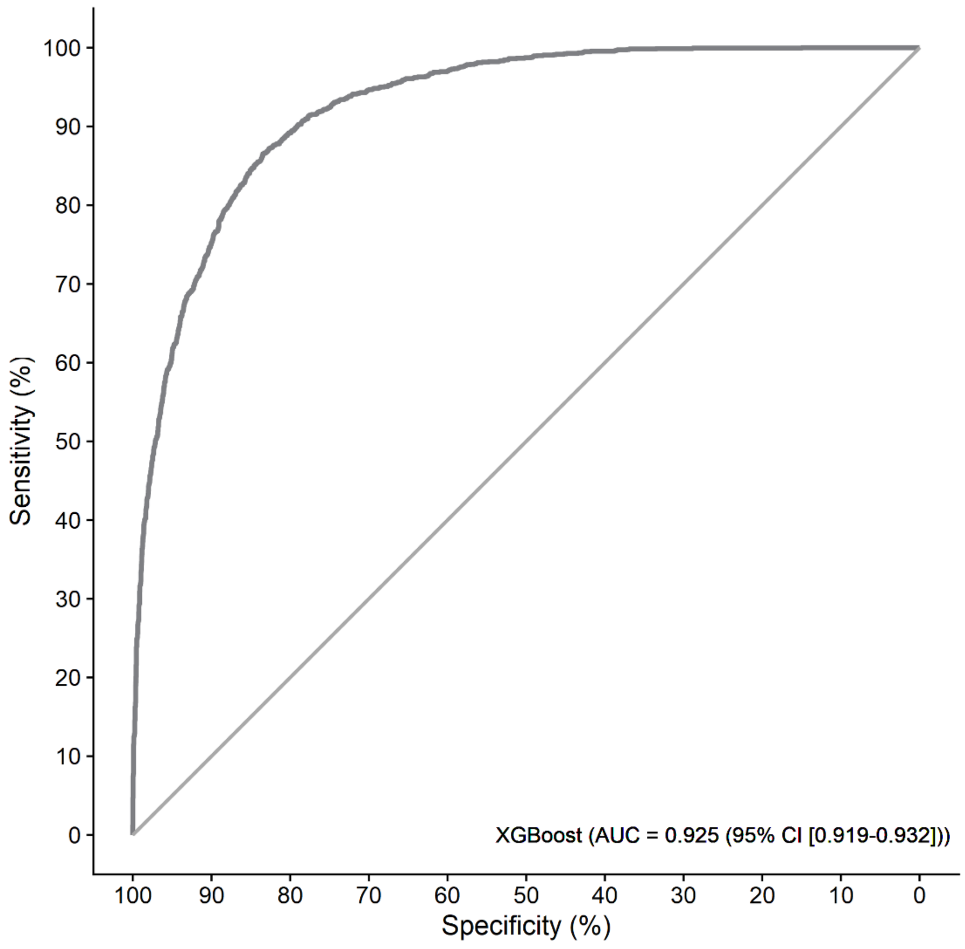

B) Receiver operator curve in patients with prior heart failure for the CoDE-HF score (area under curve 0.946 [0.830-0.862])

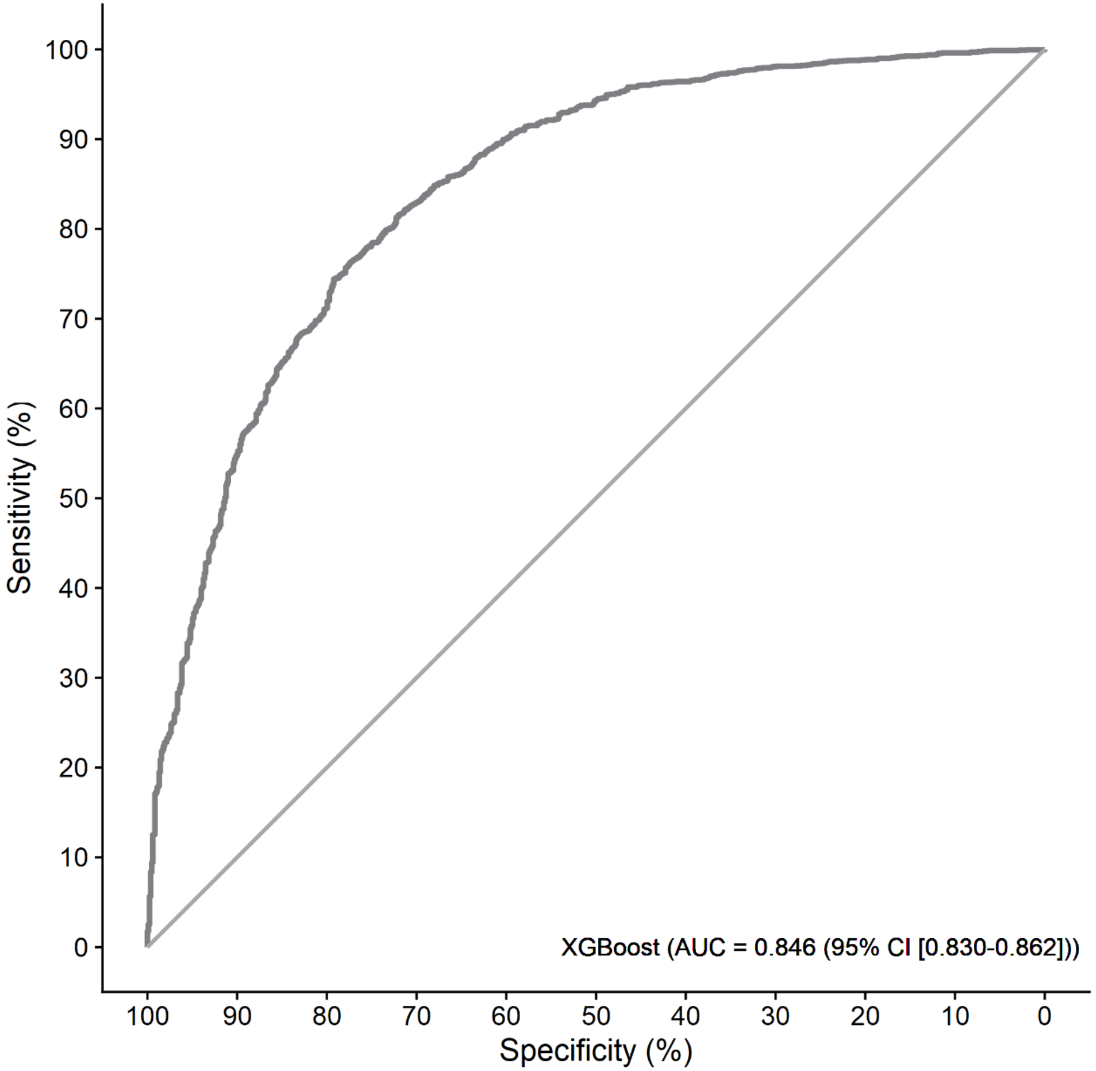

Supplementary Figure M. Decision curve analysis for CoDE-HF versus NT-proBNP alone

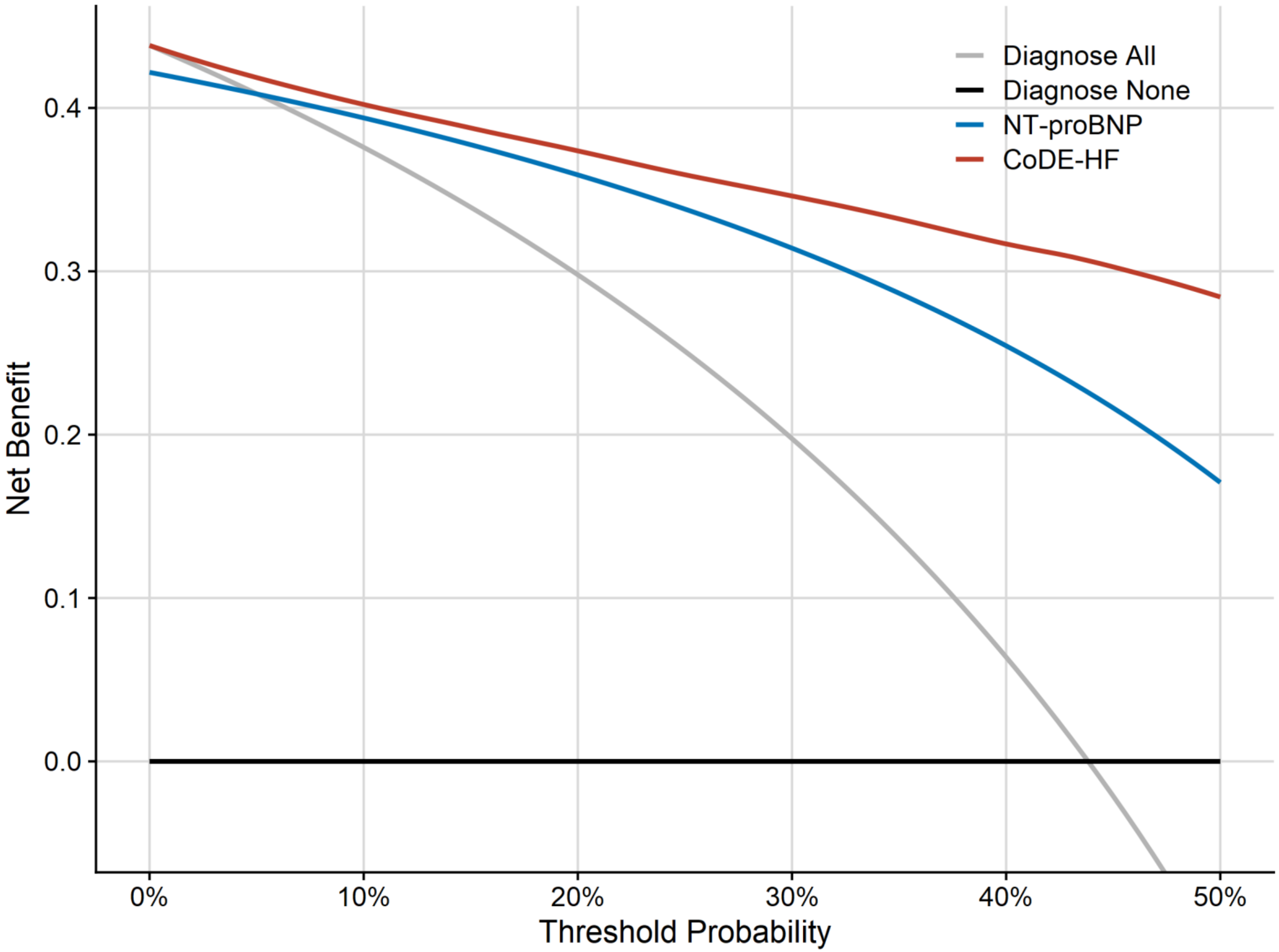

The decision curve analysis presents the net benefit of the CoDE-HF score and NT-proBNP alone in comparison to hypothetical default approaches to diagnose all patients or no patients with acute heart failure. Net benefit for each approach is calculated across a range of possible threshold probabilities.[21 22] Threshold probability is defined in this context as the minimum probability at which a diagnosis and treatment for acute heart failure is likely to be beneficial for patients. Net benefit is calculated using the following formula:

$$\text{Net benefit } = \text{sensitivity } \times \text{prevalence} - (1 - \text{specificity}) \times \ (1 - \text{prevalence}) \times \ w$$

w is the odds at the threshold probability.

Supplementary Figure N. Internal-external cross-validation of CoDE-HF

a) Patients without a prior history of heart failure across studies

Study	N	Prevalence (%)	AUC (95% CI)	Brier score
Rutten et al	391	15	0.893 (0.853-0.934)	0.094
Bahrmann et al	79	19	0.919 (0.844-0.993)	0.088
Maisel et al	1017	19	0.929 (0.912-0.946)	0.093
Ibrahim et al	870	20	0.929 (0.910-0.947)	0.086
Moe et al	277	31	0.863 (0.819-0.906)	0.164
Wussler et al	1387	35	0.931 (0.919-0.944)	0.115
Nazerian et al	115	39	0.838 (0.763-0.913)	0.221
Mueller et al	176	41	0.886 (0.838-0.933)	0.296
Januzzi et al	827	42	0.950 (0.937-0.964)	0.093
Gargani et al	103	75	0.969 (0.935-1.000)	0.235

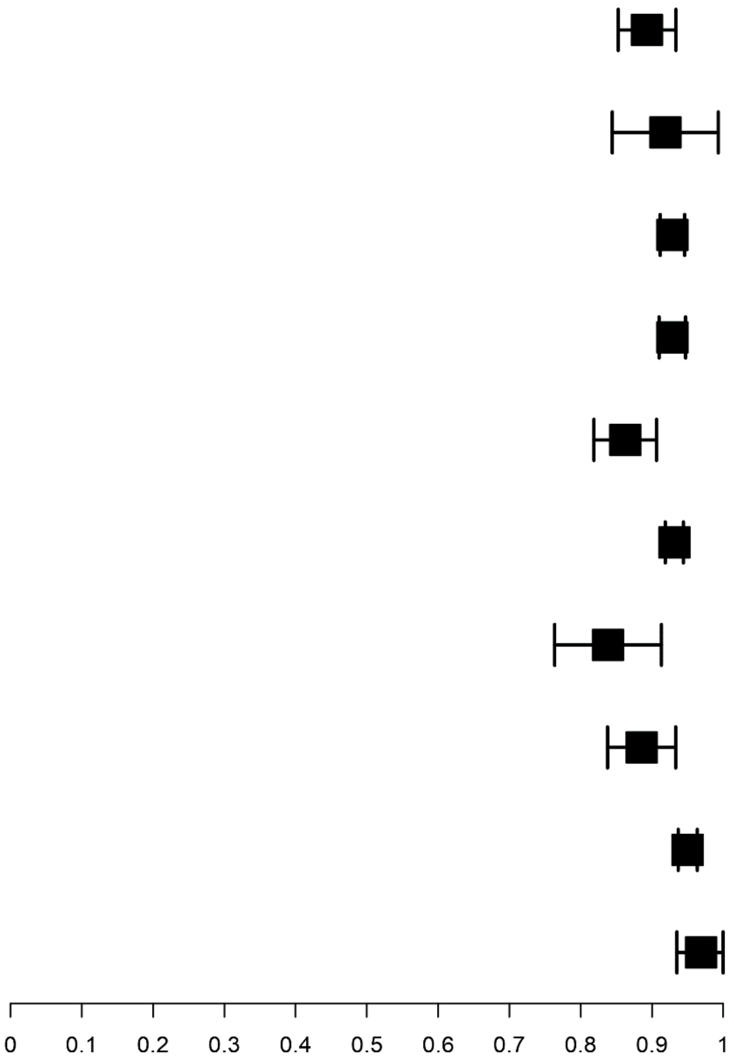

b) Patients with prior history of heart failure across studies

Study	N	Prevalence (%)	AUC (95% CI)	Brier score
Rutten et al	85	45	0.761 (0.655-0.866)	0.200
Behnes et al	194	63	0.753 (0.680-0.826)	0.196
Nazerian et al	30	63	0.842 (0.699-0.985)	0.171
Ibrahim et al	235	63	0.778 (0.715-0.842)	0.210
Maisel et al	563	64	0.849 (0.815-0.883)	0.164
Bahrmann et al	224	68	0.911 (0.873-0.949)	0.138
Moe et al	171	70	0.759 (0.677-0.841)	0.186
Wussler et al	652	84	0.878 (0.836-0.920)	0.098
Mueller et al	75	87	0.818 (0.664-0.973)	0.118
Januzzi et al	429	87	0.831 (0.770-0.893)	0.089
Gargani et al	46	98	1.000 (1.000-1.000)	0.159

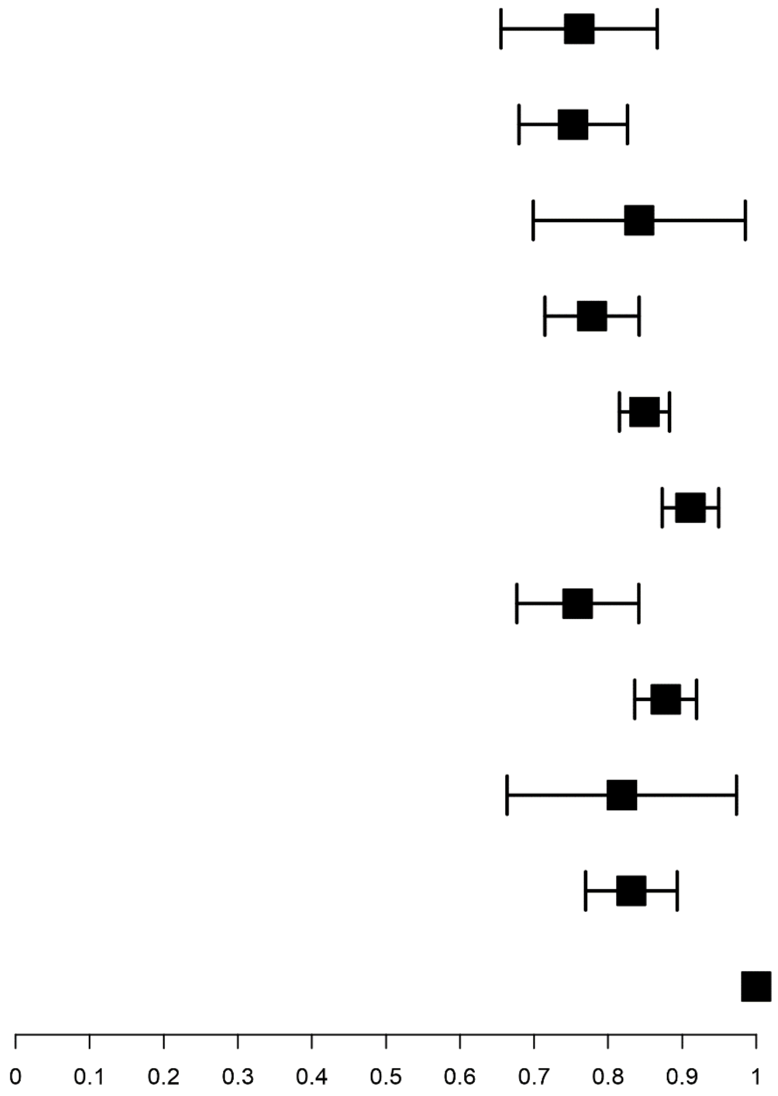

Supplementary Figure O. Cumulative incidence of all-cause mortality stratified by NT-proBNP concentration

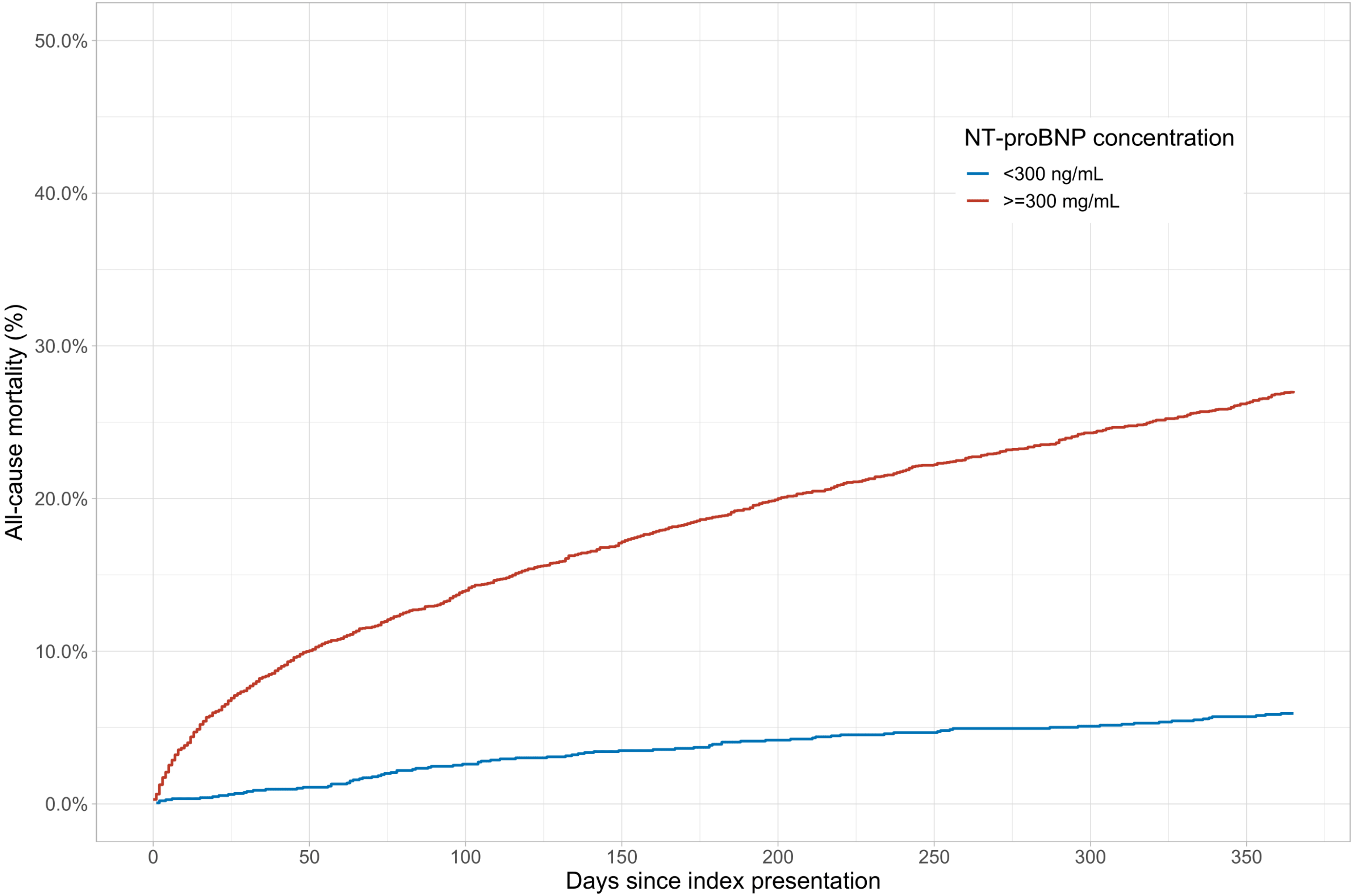

V. Checklists

PRISMA-IPD Checklist of items to include when reporting a systematic review and meta-analysis of individual participant data (IPD)

PRISMA-IPD Section/topic	Item No	Checklist item	Reported on page
**Title**			
Title	1	Identify the report as a systematic review and meta-analysis of individual participant data.	-
**Abstract**			
Structured summary	2	Provide a structured summary including as applicable:	2
		**Background**: state research question and main objectives, with information on participants, interventions, comparators and outcomes.	
		**Methods**: report eligibility criteria; data sources including dates of last bibliographic search or elicitation, noting that IPD were sought; methods of assessing risk of bias.	
		**Results**: provide number and type of studies and participants identified and number (%) obtained; summary effect estimates for main outcomes (benefits and harms) with confidence intervals and measures of statistical heterogeneity. Describe the direction and size of summary effects in terms meaningful to those who would put findings into practice.	
		**Discussion:** state main strengths and limitations of the evidence, general interpretation of the results and any important implications.	
		**Other:** report primary funding source, registration number and registry name for the systematic review and IPD meta-analysis.	
**Introduction**			
Rationale	3	Describe the rationale for the review in the context of what is already known.	4
Objectives	4	Provide an explicit statement of the questions being addressed with reference, as applicable, to participants, interventions, comparisons, outcomes and study design (PICOS). Include any hypotheses that relate to particular types of participant-level subgroups.	5
**Methods**			
Protocol and registration	5	Indicate if a protocol exists and where it can be accessed. If available, provide registration information including registration number and registry name. Provide publication details, if applicable.	6
Eligibility criteria	6	Specify inclusion and exclusion criteria including those relating to participants, interventions, comparisons, outcomes, study design and characteristics (e.g. years when conducted, required minimum follow-up). Note whether these were applied at the study or individual level i.e. whether eligible participants were included (and ineligible participants excluded) from a study that included a wider population than specified by the review inclusion criteria. The rationale for criteria should be stated.	6

Identifying studies - information sources	7	Describe all methods of identifying published and unpublished studies including, as applicable: which bibliographic databases were searched with dates of coverage; details of any hand searching including of conference proceedings; use of study registers and agency or company databases; contact with the original research team and experts in the field; open adverts and surveys. Give the date of last search or elicitation.	6
Identifying studies - search	8	Present the full electronic search strategy for at least one database, including any limits used, such that it could be repeated.	Supplement, page 6
Study selection processes	9	State the process for determining which studies were eligible for inclusion.	6
Data collection processes	10	Describe how IPD were requested, collected and managed, including any processes for querying and confirming data with investigators. If IPD were not sought from any eligible study, the reason for this should be stated (for each such study).	6
		If applicable, describe how any studies for which IPD were not available were dealt with. This should include whether, how and what aggregate data were sought or extracted from study reports and publications (such as extracting data independently in duplicate) and any processes for obtaining and confirming these data with investigators.	
Data items	11	Describe how the information and variables to be collected were chosen. List and define all study level and participant level data that were sought, including baseline and follow-up information. If applicable, describe methods of standardising or translating variables within the IPD datasets to ensure common scales or measurements across studies.	6
IPD integrity	A1	Describe what aspects of IPD were subject to data checking (such as sequence generation, data consistency and completeness, baseline imbalance) and how this was done.	6
Risk of bias assessment in individual studies.	12	Describe methods used to assess risk of bias in the individual studies and whether this was applied separately for each outcome. If applicable, describe how findings of IPD checking were used to inform the assessment. Report if and how risk of bias assessment was used in any data synthesis.	6-7
Specification of outcomes and effect measures	13	State all treatment comparisons of interests. State all outcomes addressed and define them in detail. State whether they were pre-specified for the review and, if applicable, whether they were primary/main or secondary/additional outcomes. Give the principal measures of effect (such as risk ratio, hazard ratio, difference in means) used for each outcome.	7
Synthesis methods	14	Describe the meta-analysis methods used to synthesise IPD. Specify any statistical methods and models used. Issues should include (but are not restricted to):<br>• Use of a one-stage or two-stage approach.<br>• How effect estimates were generated separately within each study and combined across studies (where applicable).<br>• Specification of one-stage models (where applicable) including how clustering of patients within studies was accounted for.<br>• Use of fixed or random effects models and any other model assumptions, such as proportional hazards.<br>• How (summary) survival curves were generated (where applicable).<br>• Methods for quantifying statistical heterogeneity (such as I^2 and τ^2).<br>• How studies providing IPD and not providing IPD were analysed together (where applicable).	7

		• How missing data within the IPD were dealt with (where applicable).	
Exploration of variation in effects	A2	If applicable, describe any methods used to explore variation in effects by study or participant level characteristics (such as estimation of interactions between effect and covariates). State all participant-level characteristics that were analysed as potential effect modifiers, and whether these were pre-specified.	7
Risk of bias across studies	15	Specify any assessment of risk of bias relating to the accumulated body of evidence, including any pertaining to not obtaining IPD for particular studies, outcomes or other variables.	6-7
Additional analyses	16	Describe methods of any additional analyses, including sensitivity analyses. State which of these were pre-specified.	7-9
**Results**			
Study selection and IPD obtained	17	Give numbers of studies screened, assessed for eligibility, and included in the systematic review with reasons for exclusions at each stage. Indicate the number of studies and participants for which IPD were sought and for which IPD were obtained. For those studies where IPD were not available, give the numbers of studies and participants for which aggregate data were available. Report reasons for non-availability of IPD. Include a flow diagram.	Supplement page 33
Study characteristics	18	For each study, present information on key study and participant characteristics (such as description of interventions, numbers of participants, demographic data, unavailability of outcomes, funding source, and if applicable duration of follow-up). Provide (main) citations for each study. Where applicable, also report similar study characteristics for any studies not providing IPD.	Supplement page 18-22
IPD integrity	A3	Report any important issues identified in checking IPD or state that there were none.	11 and Supplement, page 32
Risk of bias within studies	19	Present data on risk of bias assessments. If applicable, describe whether data checking led to the up-weighting or down-weighting of these assessments. Consider how any potential bias impacts on the robustness of meta-analysis conclusions.	11
Results of individual studies	20	For each comparison and for each main outcome (benefit or harm), for each individual study report the number of eligible participants for which data were obtained and show simple summary data for each intervention group (including, where applicable, the number of events), effect estimates and confidence intervals. These may be tabulated or included on a forest plot.	Supplement,page 34-35, 46-47
Results of syntheses	21	Present summary effects for each meta-analysis undertaken, including confidence intervals and measures of statistical heterogeneity. State whether the analysis was pre-specified, and report the numbers of studies and participants and, where applicable, the number of events on which it is based.	10-11
		When exploring variation in effects due to patient or study characteristics, present summary interaction estimates for each characteristic examined, including confidence intervals and measures of statistical heterogeneity. State whether the analysis was pre-specified. State whether any interaction is consistent across trials.	
		Provide a description of the direction and size of effect in terms meaningful to those who would put findings into practice.	

Risk of bias across studies	22	Present results of any assessment of risk of bias relating to the accumulated body of evidence, including any pertaining to the availability and representativeness of available studies, outcomes or other variables.	Supplement, page 21
Additional analyses	23	Give results of any additional analyses (e.g. sensitivity analyses). If applicable, this should also include any analyses that incorporate aggregate data for studies that do not have IPD. If applicable, summarise the main meta-analysis results following the inclusion or exclusion of studies for which IPD were not available.	11-13
**Discussion**			
Summary of evidence	24	Summarise the main findings, including the strength of evidence for each main outcome.	14
Strengths and limitations	25	Discuss any important strengths and limitations of the evidence including the benefits of access to IPD and any limitations arising from IPD that were not available.	14 & 17
Conclusions	26	Provide a general interpretation of the findings in the context of other evidence.	18
Implications	A4	Consider relevance to key groups (such as policy makers, service providers and service users). Consider implications for future research.	16
**Funding**			
Funding	27	Describe sources of funding and other support (such as supply of IPD), and the role in the systematic review of those providing such support.	20

A1 – A3 denote new items that are additional to standard PRISMA items. A4 has been created as a result of re-arranging content of the standard PRISMA statement to suit the way that systematic review IPD meta-analyses are reported.

© Reproduced with permission of the PRISMA IPD Group, which encourages sharing and reuse for non-commercial purpose

TRIPOD Checklist: Prediction Model Development and Validation

Section/Topic	Item		Checklist Item	Page
Title and abstract				
Title	1	D;V	Identify the study as developing and/or validating a multivariable prediction model, the target population, and the outcome to be predicted.	1
Abstract	2	D;V	Provide a summary of objectives, study design, setting, participants, sample size, predictors, outcome, statistical analysis, results, and conclusions.	2-3
Introduction				
Background and objectives	3a	D;V	Explain the medical context (including whether diagnostic or prognostic) and rationale for developing or validating the multivariable prediction model, including references to existing models.	4-5
	3b	D;V	Specify the objectives, including whether the study describes the development or validation of the model or both.	5
Methods				
Source of data	4a	D;V	Describe the study design or source of data (e.g., randomized trial, cohort, or registry data), separately for the development and validation data sets, if applicable.	6
	4b	D;V	Specify the key study dates, including start of accrual; end of accrual; and, if applicable, end of follow-up.	6
Participants	5a	D;V	Specify key elements of the study setting (e.g., primary care, secondary care, general population) including number and location of centres.	6
	5b	D;V	Describe eligibility criteria for participants.	6
	5c	D;V	Give details of treatments received, if relevant.	NA
Outcome	6a	D;V	Clearly define the outcome that is predicted by the prediction model, including how and when assessed.	6
	6b	D;V	Report any actions to blind assessment of the outcome to be predicted.	6
Predictors	7a	D;V	Clearly define all predictors used in developing or validating the multivariable prediction model, including how and when they were measured.	8
	7b	D;V	Report any actions to blind assessment of predictors for the outcome and other predictors.	NA
Sample size	8	D;V	Explain how the study size was arrived at.	7-8
Missing data	9	D;V	Describe how missing data were handled (e.g., complete-case analysis, single imputation, multiple imputation) with details of any imputation method.	8
Statistical analysis methods	10a	D	Describe how predictors were handled in the analyses.	8
	10b	D	Specify type of model, all model-building procedures (including any predictor selection), and method for internal validation.	8
	10c	V	For validation, describe how the predictions were calculated.	8
	10d	D;V	Specify all measures used to assess model performance and, if relevant, to compare multiple models.	9
	10e	V	Describe any model updating (e.g., recalibration) arising from the validation, if done.	NA
Risk groups	11	D;V	Provide details on how risk groups were created, if done.	9
Development vs. validation	12	V	For validation, identify any differences from the development data in setting, eligibility criteria, outcome, and predictors.	7-8
Results				
Participants	13a	D;V	Describe the flow of participants through the study, including the number of participants with and without the outcome and, if applicable, a summary of the follow-up time. A diagram may be helpful.	10
	13b	D;V	Describe the characteristics of the participants (basic demographics, clinical features, available predictors), including the number of participants with missing data for predictors and outcome.	10
	13c	V	For validation, show a comparison with the development data of the distribution of important variables (demographics, predictors and outcome).	10
Model development	14a	D	Specify the number of participants and outcome events in each analysis.	10
	14b	D	If done, report the unadjusted association between each candidate predictor and outcome.	10
Model specification	15a	D	Present the full prediction model to allow predictions for individuals (i.e., all regression coefficients, and model intercept or baseline survival at a given time point).	NA
	15b	D	Explain how to the use the prediction model.	11
Model performance	16	D;V	Report performance measures (with CIs) for the prediction model.	11-12
Model-updating	17	V	If done, report the results from any model updating (i.e., model specification, model performance).	NA
Discussion				
Limitations	18	D;V	Discuss any limitations of the study (such as nonrepresentative sample, few events per predictor, missing data).	17
Interpretation	19a	V	For validation, discuss the results with reference to performance in the development data, and any other validation data.	16
	19b	D;V	Give an overall interpretation of the results, considering objectives, limitations, results from similar studies, and other relevant evidence.	14
Implications	20	D;V	Discuss the potential clinical use of the model and implications for future research.	16
Other information				
Supplementary information	21	D;V	Provide information about the availability of supplementary resources, such as study protocol, Web calculator, and data sets.	7
Funding	22	D;V	Give the source of funding and the role of the funders for the present study.	20

*Items relevant only to the development of a prediction model are denoted by D, items relating solely to a validation of a prediction model are denoted by V, and items relating to both are denoted D;V. We recommend using the TRIPOD Checklist in conjunction with the TRIPOD Explanation and Elaboration document.

References

1. Chen T, Guestrin C. XGBoost: A Scalable Tree Boosting System. *ArXiv e-prints* 2016
2. Friedman JH. Greedy Function Approximation: A Gradient Boosting Machine. *The Annals of Statistics* 2001;29(5):1189-232.
3. Friedman J, Hastie T, Tibshirani R. Additive logistic regression: a statistical view of boosting. *Ann Statist* 2000;28(2):337-407.
4. Royston P, Ambler G, Sauerbrei W. The use of fractional polynomials to model continuous risk variables in epidemiology. *Int J Epidemiol* 1999;28(5):964-74.
5. George, Langley P. Estimating Continuous Distributions in Bayesian Classifiers. *arXiv pre-print server* 2013
6. Breiman L. *Machine Learning* 2001;45(1):5-32.
7. Bahrmann P, Bahrmann A, Hofner B, et al. Multiple biomarker strategy for improved diagnosis of acute heart failure in older patients presenting to the emergency department. *Eur Heart J Acute Cardiovasc Care* 2015;4(2):137-47.
8. Behnes M, Brueckmann M, Ahmad-Nejad P, et al. Diagnostic performance and cost effectiveness of measurements of plasma N-terminal pro brain natriuretic peptide in patients presenting with acute dyspnea or peripheral edema. *Int J Cardiol* 2009;135(2):165–74.
9. Bombelli M, Maloberti A, Rossi S, et al. Clinical value of NT-proBNP assay in the emergency department for the diagnosis of heart failure (HF) in very elderly people. *Arch Gerontol Geriatr* 2015;61(2):296-300.
10. Chenevier-Gobeaux C, Claessens YE, Voyer S, et al. Influence of renal function on N-terminal pro-brain natriuretic peptide (NT-proBNP) in patients admitted for dyspnoea in the Emergency Department: comparison with brain natriuretic peptide (BNP). *Clin Chim Acta* 2005;361(1-2):167–75.
11. deFilippi CR, Seliger SL, Maynard S, et al. Impact of renal disease on natriuretic peptide testing for diagnosing decompensated heart failure and predicting mortality. *Clin Chem* 2007;53(8):1511-9.
12. Gargani L, Frassi F, Soldati G, et al. Ultrasound lung comets for the differential diagnosis of acute cardiogenic dyspnoea: a comparison with natriuretic peptides. *European journal of heart failure* 2008;10(1):70–7.
13. Ibrahim I, Kuan WS, Frampton C, et al. Superior performance of N-terminal pro brain natriuretic peptide for diagnosis of acute decompensated heart failure in an Asian

compared with a Western setting. *European journal of heart failure* 2017;19(2):209-17.

14. Januzzi JL, van Kimmenade R, Lainchbury J, et al. NT-proBNP testing for diagnosis and short-term prognosis in acute destabilized heart failure: an international pooled analysis of 1256 patients: the International Collaborative of NT-proBNP Study. *Eur Heart J* 2006;27(3):330–7.
15. Maisel A, Mueller C, Nowak R, et al. Mid-region pro-hormone markers for diagnosis and prognosis in acute dyspnea: results from the BACH (Biomarkers in Acute Heart Failure) trial. *J Am Coll Cardiol* 2010;55(19):2062–76.
16. Moe GW, Howlett J, Januzzi JL, et al. N-terminal pro-B-type natriuretic peptide testing improves the management of patients with suspected acute heart failure: primary results of the Canadian prospective randomized multicenter IMPROVE-CHF study. *Circulation* 2007;115(24):3103-10.
17. Mueller T, Gegenhuber A, Poelz W, et al. Diagnostic accuracy of B type natriuretic peptide and amino terminal proBNP in the emergency diagnosis of heart failure. *Heart* 2005;91(5):606–12.
18. Nazerian P, Vanni S, Zanobetti M, et al. Diagnostic accuracy of emergency Doppler echocardiography for identification of acute left ventricular heart failure in patients with acute dyspnea: comparison with Boston criteria and N-terminal prohormone brain natriuretic peptide. *Acad Emerg Med* 2010;17(1):18–26.
19. Rutten JH, Steyerberg EW, Boomsma F, et al. N-terminal pro-brain natriuretic peptide testing in the emergency department: beneficial effects on hospitalization, costs, and outcome. *Am Heart J* 2008;156(1):71-7.
20. Wussler D, Kozhuharov N, Sabti Z, et al. External Validation of the MEESSI Acute Heart Failure Risk Score: A Cohort Study. *Ann Intern Med* 2019;170(4):248-56.
21. Vickers AJ, Van Calster B, Steyerberg EW. Net benefit approaches to the evaluation of prediction models, molecular markers, and diagnostic tests. *BMJ* 2016;352:i6.
22. Vickers AJ, van Calster B, Steyerberg EW. A simple, step-by-step guide to interpreting decision curve analysis. *Diagnostic and Prognostic Research* 2019;3(1):18.
